# Tension Stiffening and Cracking Behavior of Axially Loaded Alkali-Activated Concrete

**DOI:** 10.3390/ma16114120

**Published:** 2023-05-31

**Authors:** Hamdi Abdulrahman, Rahimah Muhamad, Ahmad Azim Shukri, Amin Al-Fakih, Gamal Alqaifi, Ayad Mutafi, Husam S. Al-Duais, Abdulnaser M. Al-Sabaeei

**Affiliations:** 1 Razak Faculty of Technology and Informatics, Universiti Teknologi Malaysia, Kuala Lumpur 54100, Malaysia; rahimah.kl@utm.my; 2 Faculty of Civil Engineering, Universiti Teknologi Malaysia, Johor Bahru 81310, Malaysia; ahmadazim@utm.my; 3 Interdisciplinary Research Center for Construction and Building Materials, King Fahd University of Petroleum and Minerals, Dhahran 31261, Saudi Arabia; 4 School of Built Environment, Faculty of Design, Architecture & Building, University of Technology Sydney, Ultimo, NSW 2007, Australia; gamal.al-qaifi@uts.edu.au; 5 Jamilus Research Centre for Sustainable Construction (JRC-SC), Faculty of Civil Engineering and Built Environment, Universiti Tun Hussein Onn Malaysia, Parit Raja 86400, Malaysia; ayad@uthm.edu.my; 6 Department of Architecture, Faculty of Built Environment, Universiti Malaya, Kuala Lumpur 50603, Malaysia; haldoais@yahoo.com; 7 Department of Civil Engineering, Faculty of Engineering, Thamar University, Dhamar 87246, Yemen; abdulnasseralsabie@gmail.com

**Keywords:** alkali-activated concrete, tension stiffening, concrete cracking, compressive strengths, OPC codes of practice

## Abstract

Alkali-activated concrete is an eco-friendly construction material that is used to preserve natural resources and promote sustainability in the construction industry. This emerging concrete consists of fine and coarse aggregates and fly ash that constitute the binder when mixed with alkaline activators, such as sodium hydroxide (NaOH) and sodium silicate (Na_2_SiO_3_). However, understanding its tension stiffening and crack spacing and width is of critical importance in fulfilling serviceability requirements. Therefore, this research aims to evaluate the tension stiffening and cracking performance of alkali-activated (AA) concrete. The variables considered in this study were compressive strength (f_c_) and concrete cover-to-bar diameter (C_c_/d_b_) ratios. After casting the specimen, they were cured before testing at ambient curing conditions for 180 days to reduce the effects of concrete shrinkage and obtain more realistic cracking results. The results showed that both AA and OPC concrete prisms develop slightly similar axial cracking force and corresponding cracking strain, but OPC concrete prisms exhibited a brittle behavior, resulting in a sudden drop in the load–strain curves at the crack location. In contrast, AA concrete prisms developed more than one crack simultaneously, suggesting a more uniform tensile strength compared to OPC specimens. The tension-stiffening factor (β) of AA concrete exhibited better ductile behavior than OPC concrete due to the strain compatibility between concrete and steel even after crack ignition. It was also observed that increasing the confinement (C_c_/d_b_ ratio) around the steel bar delays internal crack formation and enhances tension stiffening in AAC. Comparing the experimental crack spacing and width with the values predicted using OPC codes of practice, such as EC2 and ACI 224R, revealed that EC2 tends to underestimate the maximum crack width, while ACI 224R provided better predictions. Thus, models to predict crack spacing and width have been proposed accordingly.

## 1. Introduction

Concrete made with Ordinary Portland Cement (OPC) has been commonly used as a construction material for various types of structures for almost 200 years [1,2]. Nowadays, it is the second most required material after water on earth, with hundreds of millions of tons used annually [3,4]. To supply concrete on such a large scale, the production of OPC has increased enormously, reaching a maximum of about 4 billion tons annually, and the demand for OPC is expected to increase further in the next few years [5,6]. However, the production of OPC requires high temperatures, consumes large quantities of natural resources, and contributes substantially to the greenhouse gas footprints [2,7,8]. In addition, the growing amount of fly ash (FA) by-products due to coal combustion in power plants represents another serious threat that negatively impacts the environment. FA is the fifth largest raw substance globally, with a massive annual production of approximately 800 million tons [3,9,10,11,12]. It is classified into two types as described by ASTM C618 [13]; high calcium fly ash (HCFA) and low calcium fly ash (LCFA) based on CaO content. Furthermore, HCFA is generated due to burning sub-bituminous and lignite, while LCFA is generally produced from the combustion of anthracite and bituminous [9]. Despite the vast production and the cementitious properties of FA, its consumption does not exceed 30% of its production [14]. The limited applications of AA concrete are due to a lack of knowledge on its structural behavior of reinforced concrete beams, columns, and slabs because most of the literature focuses on micro-scale investigation, such as mix design. The remaining FA is often disposed to landfills, contaminating nearby soil, and making it unfavorable for most crops [14]. Therefore, seeking an eco-friendly alternative to OPC concrete is crucial to prevent the environment by utilizing FA by-products and significantly reducing OPC consumption, resulting in smaller greenhouse gas footprints [15,16].

Recently, there has been an increased motivation among concrete practitioners and researchers to explore the potential of using AA concrete as an eco-friendly alternative to OPC concrete. In addition to the fine and coarse aggregates, FA is a primary component in AA concrete that is produced through mixing all previous components with an alkaline activator, such as sodium hydroxide (NaOH) and sodium silicate (Na_2_SiO_3_), in a process called geopolymerization [14]. The partial replacement of OPC with FA in OPC concrete improves its structural performance. In addition to the better mechanical properties, the blended OPC concrete exhibited quasi-plastic failure rather than brittle due to the addition of FA [2,17,18,19,20]. Adding FA to OPC concrete members subjected to impact and dynamic loads is also recommended to enhance the microcrack resistance [21]. The complete replacement of OPC concrete with FA has been widely assessed [22,23,24,25,26,27,28,29,30,31]. However, most of the previous studies on AA concrete have focused mainly on the mix design and micro-scale investigation [22,23,24], which supports the potential of FA as a promising building material [24,25,26,27,28,29,30,31].

Before introducing AA concrete into actual engineering applications, it should be characterized by a comparable or better structural performance to OPC concrete at the serviceability and ultimate limit state conditions, such as cracking. Cracks are considered a complex concrete property that requires thorough understanding [32]. It depends mainly significantly on tension stiffening, which can be defined as the ability of the intact concrete between cracks to carry tensile stresses [33]. Both cracking and tension stiffening play a vital role in the service limit state and are governed by the interaction between the reinforcement and surrounding concrete. Furthermore, it is related to the tensile capacity of concrete and bar slipping [34,35,36]. Hence, understanding the properties of AA concrete before introducing it into actual engineering applications is critical to fulfilling the serviceability requirements.

**Figure 1:**
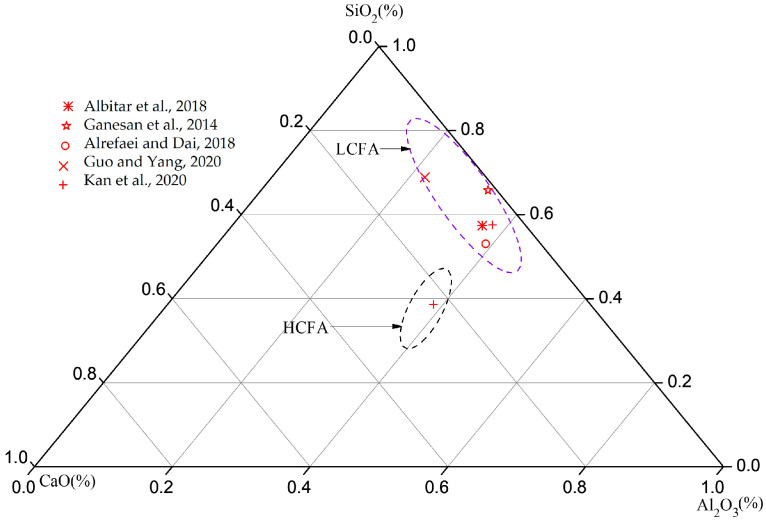
The ternary oxide system (SiO_2_, Al_2_O_3_, and CaO) for fly ash used previously for tensile and cracking investigation [27,28,40,41,42].

By referring to Figure 1, a few studies have initiated the evaluation of the structural performance of AA concrete, such as the tensile and cracking behavior [26,27,37,38,39,40,41]. The results indicate that FA exhibits high strain capacity, better tensile response, comparable cracking spacing, and width to OPC concrete. However, these studies were limited to dog bone shape specimens that might give irrational results due to the size effects. In addition, these studies employed heat curing for LCFA binder to accelerate the pozzolanic reactions that are responsible for the strength gain [42], but this approach is usually not applicable in cast–in situ construction. On the other hand, HCFA AA concrete has not received significant attention from researchers; its tension stiffening and crack spacing and width are still not well understood, and no crack spacing and width models that can be implemented in practice are currently available. This provided the impetus for the current research to focus on AA concrete based on a precursor derived from HCFA binder as an alternative to OPC concrete [43].

### Research Significance

The increasing amount of industrial waste produced due to coal combustion at power plants worldwide has dwindled the space for landfills. Moreover, these wastes are not biodegradable, and much money is spent on disposal operations. Meanwhile, the increasing demand for OPC concrete has contributed significantly to greenhouse gas emissions and the depletion of natural resources. Therefore, the present study is intended to examine the potential of AA concrete as an alternative to OPC concrete, thus encouraging innovation, preserving natural resources, and promoting environmental sustainability. Furthermore, it is also a step toward decreasing the overall construction cost as AA concrete is manufactured from industrial waste materials.

The outcomes of the present study on tension stiffening and cracking performance are expected to help better understand the serviceability limit state behavior of AA concrete. This is because the tension-stiffening behavior plays a significant role in the width and spacing of cracks, stiffness, and deformation of the structural member; thus, it is often the governing design criterion for longer-span reinforced concrete members. Understanding tension stiffening also enables engineers to perform accurate deflection calculations for serviceability limit state analysis. This is also significant when dealing with deformability analysis, particularly when cracking is involved, as it is a major source of nonlinearity. Furthermore, relying on the existing models of OPC concrete to estimate the crack spacing and crack width of AA concrete could lead to an unsafe or suboptimal design of structural members; the developed crack spacing and crack width models in this study could provide a safer design basis for structural engineers. Therefore, this study aims to investigate the effects of compressive strength and concrete cover-to-bar diameter (C_c_/d_b_) ratio on tension stiffening, crack spacing, and crack width and propose models for crack spacing and width based on the experimental results.

## 2. Experimental Program

### 2.1. Materials

This study used two types of concrete: FA-AA concrete and OPC concrete. The FA was collected from the Kapar power plant located in Selangor, Malaysia. Its chemical compositions were determined using X-ray fluorescence (XRF) and are presented in Table 1. The summation content of the SiO_2_, Al_2_O_3_, and Fe_2_O_3_ of FA is in the range of 50–70%, and the CaO content is greater than 10%. Thus, The FA is classified as HCFA according to ASTM C618 [12]. The OPC concrete was used for comparison purposes only.

The alkaline activator solution employed in this study was a combination of premixed sodium silicate (Na_2_SiO_3_) and 14M sodium hydroxide (NaOH). The percentage of Na_2_SiO_3_ solution to the 14M NaOH was maintained at 1.5 by weight for all mixes, as suggested by Abdulrahman et al. [44]. The proportions of the three components of the AA solution were 62.64% water, 24.64% Na_2_SiO_3_ and 12.72% NaOH by weight. It is worth mentioning that the three optimum concrete admixtures developed by Abdulrahman et al. [44] were also used in the current study, as the FA used in both studies is identical. These concrete admixtures are also presented in Table 2 and remarked as mix 5 (M5), mix 8 (M8), and mix 9 (M9). Borax was also used to prolong the initial setting time of AA concrete. The deformed steel bar used for casting the tension-stiffening prisms was either 10 mm, 12 mm, or 16 mm in diameter. The mechanical properties of these reinforcements were determined using a tensile test carried out on three bars of each diameter and are presented in Table 3.

### 2.2. Specimen Design and Instrumentation

The experimental program was designed to produce a comprehensive database of uniaxial tension tests of AA concrete prisms. The test matrix consisted of a total of 38 reinforced concrete prisms. Four specimens were manufactured from OPC concrete: two samples with a steel bar diameter of 12 mm, and the other two prisms with a 16 mm steel bar diameter. These prisms were used as control specimens. The remaining 34 prisms were produced from AA concrete and were divided into three groups based on the admixture proportions and steel bar size, as shown in the test matrix (Table 4). In addition to the admixture number (i.e., M5), the specimens were marked by the letters FA, which shows the fly ash as the binder material, followed by the steel bar diameter used (i.e., 10). The last alphabet, “A, B, C and D” corresponds to the specimen number (i.e., M5-FA-10-A). All specimens had a square cross-section of 75 mm × 75 mm and a length of 650 mm in which matrix cracking was allowed to occur. The length of the test specimen was limited to the capacity of the tensile machine. In the FA prisms, a single steel bar with deformed ribs of either 10 mm, 12 mm, or 16 mm was embedded longitudinally in the centroid of each prism, corresponding to a reinforcement ratio (ρ) varying from 1.42% to 3.71%. The compressive strength ($f_{cu}$) and splitting tensile strength ($f_{ct}$) of each group were conducted on 100 mm concrete cube specimens and 𝜙150 × 300 mm cylinders as recommended by BS EN 12390–3 [32].

**Figure 2:**
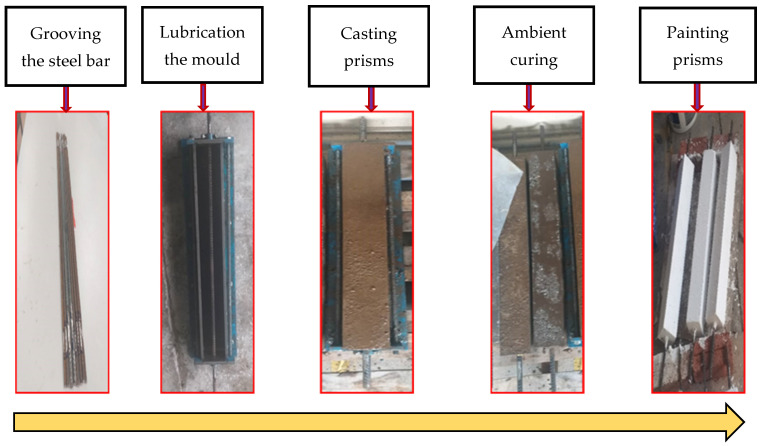
Procedures of preparing AA concrete.

**Figure 3:**
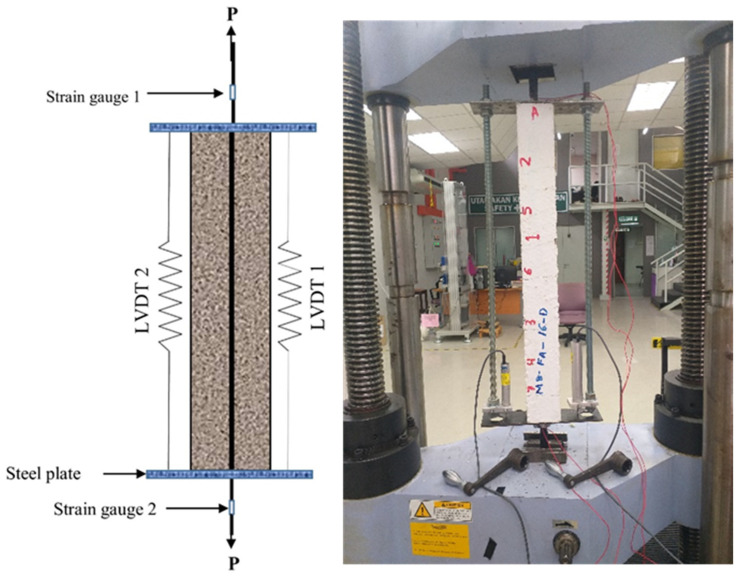
Uniaxial tensile specimen dimension and setup.

After casting the prisms, they were cured using an ambient curing regime and then demolded to be stored at ambient lab conditions for 180 days, as shown in Figure 2. The prisms were tested at this age (180 days) to reduce the effects of concrete shrinkage and obtain more realistic cracking results. It should be highlighted that painting the prisms white before conducting the tests was crucial to ease the visual tracing of cracks. Before the testing, the grooved steel bar was sanded and cleaned on both sides to facilitate the installation of two 5 mm strain gauges on each end of the steel bar to measure the average axial strain in the reinforcing steel bar. As shown in Figure 3, two linear variable displacement transformers (LVDTs) were fixed on opposite sides of the prism to measure the average axial elongation (deformation) of the reinforced concrete prism over a total length of 650 mm. The crack width was measured using a handheld digital microscope with a magnification of 220×.

### 2.3. Testing Procedures

A displacement control method was used to test all specimens. A direct progressive uniaxial load (p) was applied to the protruded part of the steel bar until its yielding limit. Although a standard test setup for concrete elements under tension does not exist, the direct tensile test is the most widely used experimental layout [44]. The load was applied at a slower rate of 0.1 mm/min in a 500 kN Shimadzu Universal Testing Machine (UTM) to monitor the deformation, the crack formation, and its corresponding force and strain readings with high accuracy, as shown in Figure 3. Whenever a new crack appeared or at about a 15 kN interval, the tests were stopped to measure the crack width, load, and strains to investigate their evolution. Then, the same procedure is followed for the next cracks until the steel bar begins yielding.

## 3. Results and Discussion

### 3.1. Characteristics of OPC and FA

**Figure 4:**
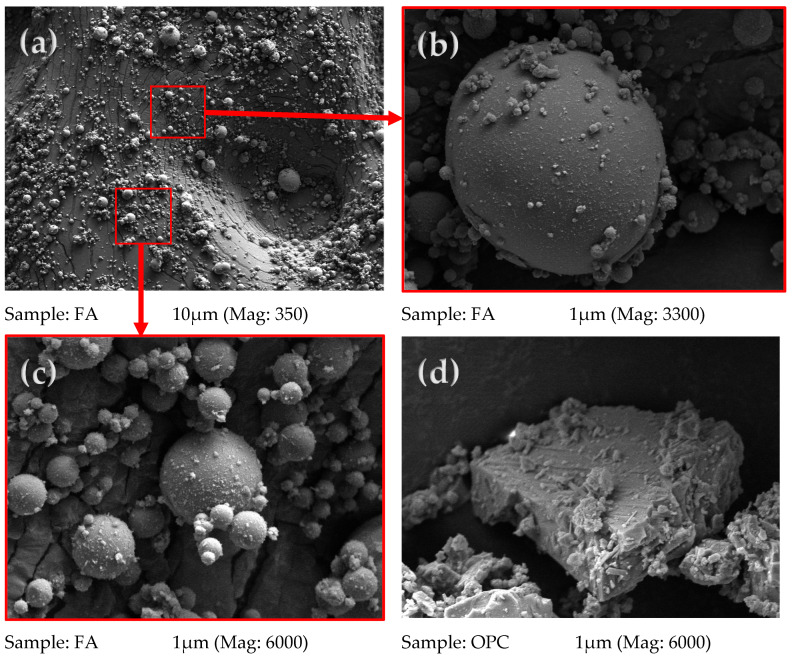
SEM images of FA and OPC; (**a**) FA grains (Mag: 350), (**b**) FA grains (Mag: 3300), (**c**) FA grains (Mag: 6000), (**d**) OPC grains (Mag: 6000).

The results of the scanning electron microscopic (SEM) analysis of the FA and OPC powder are given in Figure 4. The typical grain characteristics are shown at several magnifications. The SEM images of FA typically show spherical particles of varying sizes as well as irregularly shaped particles with rough surfaces, while the OPC image shows plate-like and irregular shaped particles. It is clear that the FA particles are distinct by their fine and spherical grains compared to the plate-like and irregular shape particles of OPC. These textures of grains explain the high tendency of FA to react with alkaline activators during the concrete mixing.

### 3.2. XRD Results

**Figure 5:**
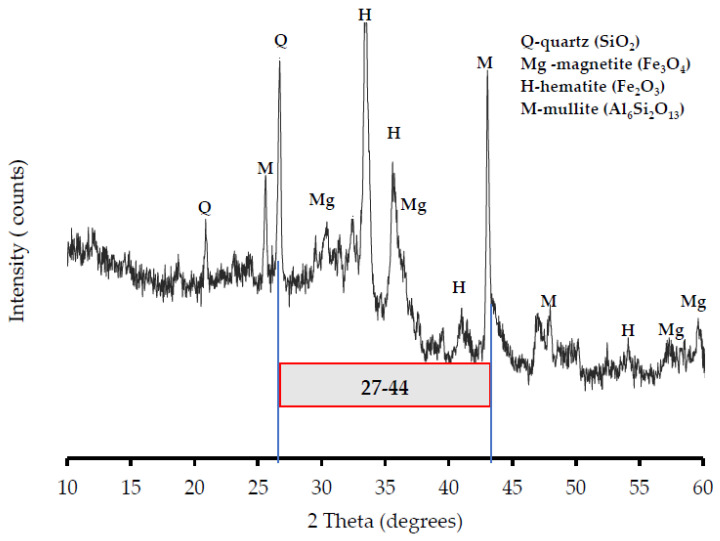
XRD pattern of FA.

The results of the X-ray diffraction (XRD) analysis of FA are given in Figure 5, indicating four significant crystalline components in the phase composition of FA. These components are quartz (SiO_2_), mullite (Al_6_Si_2_O_13_), magnetite (Fe_3_O_4_), and hematite (Fe_2_O_3_). The figure also shows that increasing the intensity occurs at angles between 27 and 44, where the components are high.

### 3.3. Tension-Stiffening Behavior of AA and OPC Prims

The experimental results of tension stiffening for AA prisms along with the OPC control prisms are summarized in Table 4. These results consist of the axial cracking load (N_cr_) and the corresponding cracking strain (ɛ_cr_) of all prisms, together with the tension force (N_sb,cr_) and strain (ɛ_sb,cr_) of reinforcement, as well as the cracking force (P_cr_) and stress (F_cr_) of concrete. The characteristics compressive strengths (f_c_) of AA concrete vary between 24 and 43 MPa, while the characteristics compressive strengths of OPC concrete were 28 MPa and 35 MPa, respectively. Despite the slight difference in concrete strength for both AA and OPC specimens, the obtained results were used to compare the mechanisms of tension stiffening and crack response for both concretes.

Although both AA and OPC prisms developed slightly similar axial cracking force (N_cr_), the corresponding cracking strain (ɛ_cr_) of OPC prisms was slightly smaller, as shown in Table 4. A possible explanation is that OPC concrete is characterized by a brittle behavior [2,18], which initially resisted the elongation, resulting in less cracking strain (ɛ_cr_). This enabled the OPC concrete to exhibit higher tensile stresses (P_cr_) before cracking, which suddenly deteriorated after the initial crack form. However, the tensile stress (P_cr_) in AA concrete was slightly lower at the initial crack, but it continued to carry more tensile stresses after cracking, resulting in a better tension stiffening than OPC concrete. These premature cracks observed in the current study are similar to those reported in the literature for AA concrete [4]. This was attributed to the residual stresses in the concrete due to shrinkage, which was pronounced because of the high restraint provided by the steel bar. In addition, the premature crack was to the high bond strength between concrete and steel that triggers strain localization at the steel bar [45,46,47]. Table 4 shows the standard deviation (S.D) of cracking force for AA concrete, which is lower than that of OPC concrete, indicating more homogeneous results obtained during the tension-stiffening tests.

#### 3.3.1. The Global Response of Prisms

**Figure 6:**
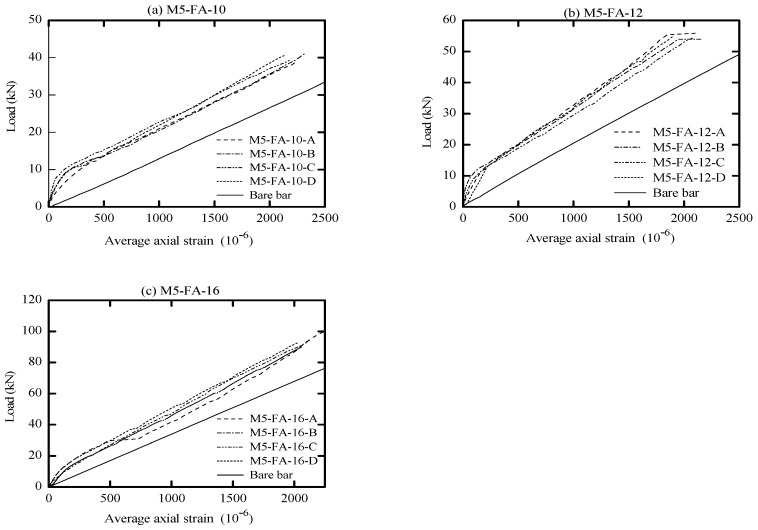
Load vs. average axial strain (10^−6^).

**Figure 7:**
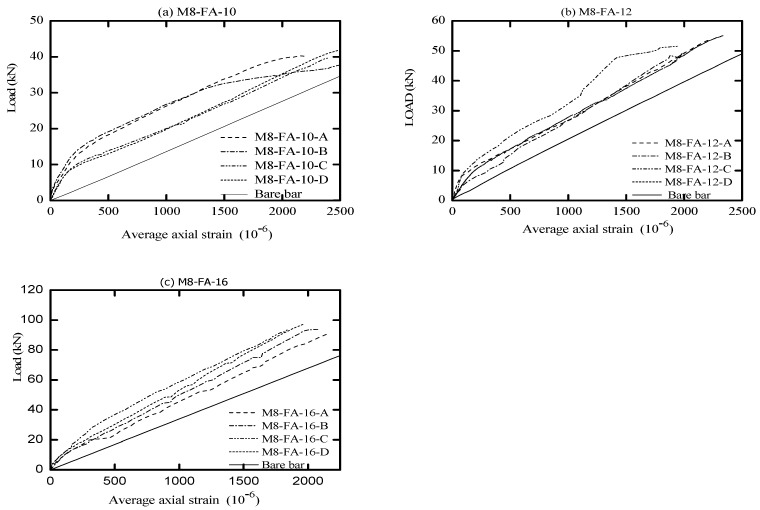
Load vs. average axial strain (10^−6^).

**Figure 8:**
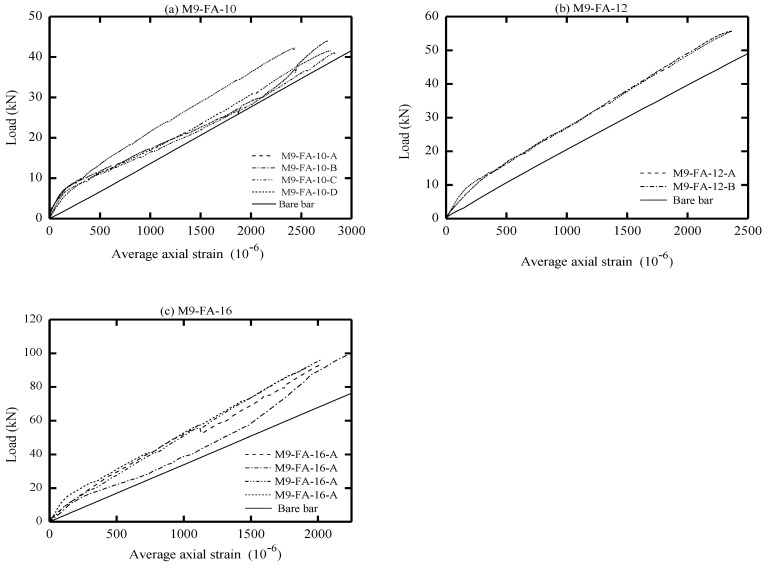
Load vs. average axial strain (10^−6^).

**Figure 9:**
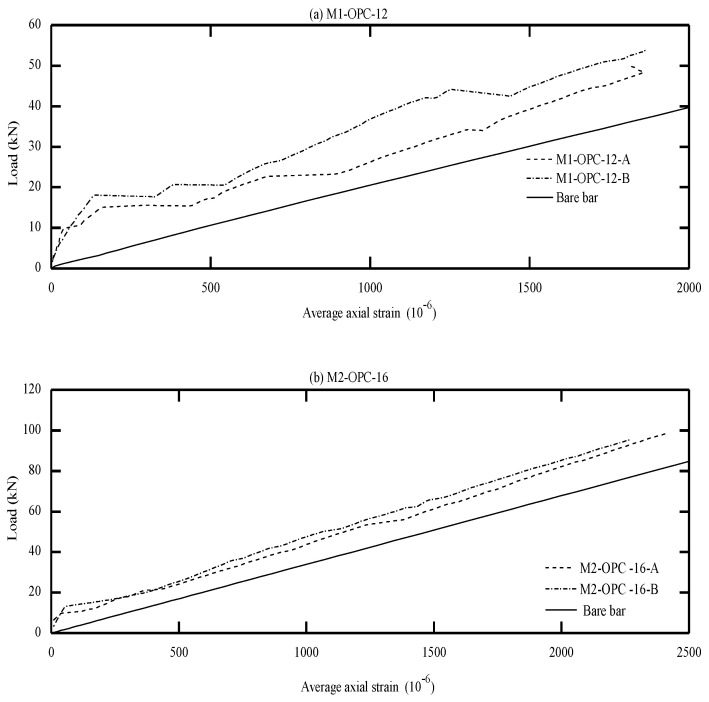
Load vs. average axial strain (10^−6^).

The global response of AA and OPC prisms and the bare bar response are given in Figure 6, Figure 7, Figure 8 and Figure 9 in terms of load-average axial strain curves (N-ɛ). The average axial strain (ɛ) represents the average value of the elongation (mm) measured by the two LVDTs fixed at the opposite sides of the tested specimens divided by the original length of the prism (650 mm), as suggested by Vilanova et al. [48]. The global response of the tested prisms was plotted up to a maximum average strain value of 2500 μm to adequately describe the tensile behavior of concrete at the service limit state, where crack and deformation control is of main importance.

As shown in Figure 6, Figure 7, Figure 8 and Figure 9, the applied tension load (N) on the tested prisms was shared between the concrete and steel bar at the initial elastic stage (before cracking) according to the material rigidity [27,49,50,51]. During this stage, the initial stiffness of specimens was higher than the stiffness of the bare bar. The concrete stresses increased due to increasing the external tension loads (N), and it started to crack when the concrete tensile capacity was exceeded. This stage is called the “cracking stage,” where cracks naturally form at the location where the tensile strength of concrete is lowest. At this stage, the tension-stiffening effects are initiated as the intact concrete between the primary cracks carries some of the tensile stresses, providing a higher overall stiffness for a cracked prism. At the cracking stage, the reinforcement is generally assumed to carry all the tension at the crack locations while the steel and concrete continue to share the tensile force between the cracks.

The formation of the new cracks continued with increasing the tension load until the crack spacing was not large enough for a new crack to form (crack-stabilized stage). This stage occurred when crack patterns in at least two consecutive load stages remained constant, and only the crack width increased. Thus, the response of the cracked prisms started to approach that of the bare bar, and the maximum applied load was limited by the yield strength of the reinforcement. This pattern agrees well with that in the literature for OPC and AA concrete [26,49,51]. The value of yield strength varies according to the employed reinforcement diameter (db); therefore, the prisms with a larger bar diameter (16 mm) show a higher loading capacity that approaches 100 kN. By referring to Figure 7b, one can see that the global response of specimen “M8-FA-12(C)” shows an early strain hardening compared to the other specimens. This is attributed to the early yielding of the reinforcement bar; its section was reduced initially by grooving to install the strain gauges.

One unusual observation can be made from Figure 9 due to the brittle fracture mechanism during the crack formation in OPC prisms. Once cracks formed in the OPC prism, the force curve experienced a sudden drop at the crack locations. This is because the OPC concrete has completely lost its capacity to carry stress at the crack locations, since it is not in contact with the steel reinforcement. Thus, the reinforcement carried the entire stress at the crack location [49,51]. From the literature, OPC concrete is characterized by high brittleness failure [19,52], and therefore, it is suggested that to avoid this brittle behavior, FA should be incorporated to produce a quasi-plastic failure [19].

Meanwhile, most of the AA prisms, observed in Figure 6, Figure 7 and Figure 8, developed more than one crack simultaneously. This rapid formation of cracks indicates that the concrete tensile strength was more uniform in AA specimens than in OPC specimens. Moreover, AA specimens exhibited a steady increase in load capacity after the concrete cracked. This demonstrates that the concrete contribution toward the overall stiffness of the prism was maintained even after the cracking. A possible reason for this could be the strain-hardening behavior of AA concrete [51].

#### 3.3.2. Tension-Stiffening Factor (β)

Tension stiffening is commonly analyzed using the normalized tensile stress in concrete that was obtained using the average strain–stress approach suggested in the literature [36,49,51,53,54,55]. This approach is characterized by realistic estimation, where the tension stiffening is obtained by subtracting the bare bar response from the total member response. This approach assumes that the steel bar and the surrounding concrete carry identical tensile strain upon the crack initiation. Correspondingly, the tensile capacity of concrete can be computed in accordance with equilibrium in Equations (1)–(3); then, it can normalized by the concrete cracking stress (p_cr_), as shown in Equation (4). This normalized tensile stress is known as the tension-stiffening factor (β), where the post-cracking phase of this factor represents the tension-stiffening contribution [36]. This factor is a material property for cracked concrete which is independent of concrete compressive strength and reinforcement ratio [26,49].

(1) $$N={\bar{N}}_{s}+{\bar{N}}_{c}$$

(2) $${\bar{N}}_{s}=A_{s}\times E_{s}\times \varepsilon_{m}$$

(3) $${\bar{N}}_{c}=N-{\bar{N}}_{s}=N-A_{s}\times E_{s}\times \varepsilon_{m}$$

(4) $$\beta =\frac{{\bar{N}}_{c}}{P_{\mathrm{cr}}}$$

where N is the axial tension load (N) applied on the member, ${\bar{N}}_{s}$ is the load in the reinforcement bar, ${\bar{N}}_{c}$ is the tensile stress in concrete, ɛ_m_ is the average member strain, P_cr_ is the concrete cracking stress, and β is the tension-stiffening bond factor.

**Figure 10:**
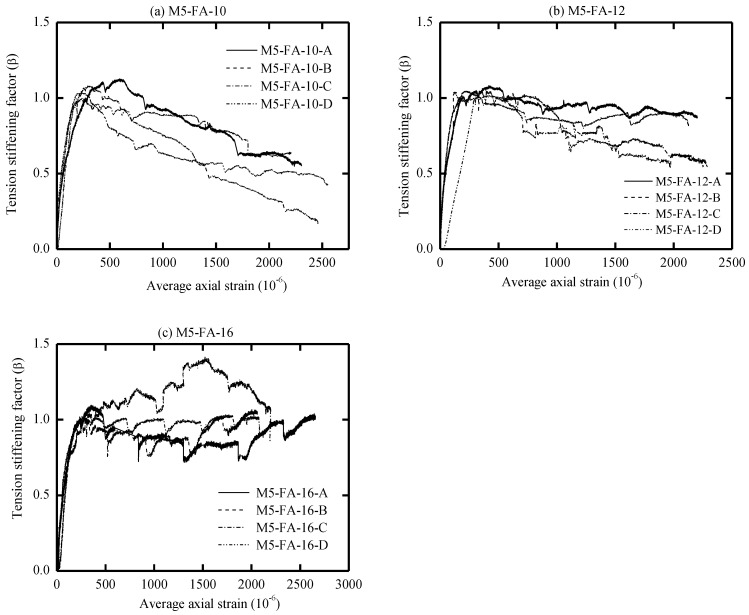
Tension-stiffening factor (β) vs. average axial strain for M5-FA.

**Figure 11:**
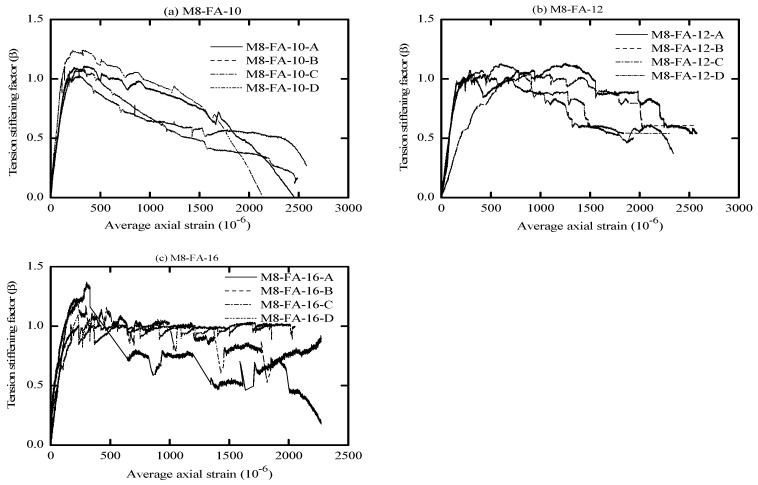
Tension-stiffening factor (β) vs. average axial strain for M8-FA.

**Figure 12:**
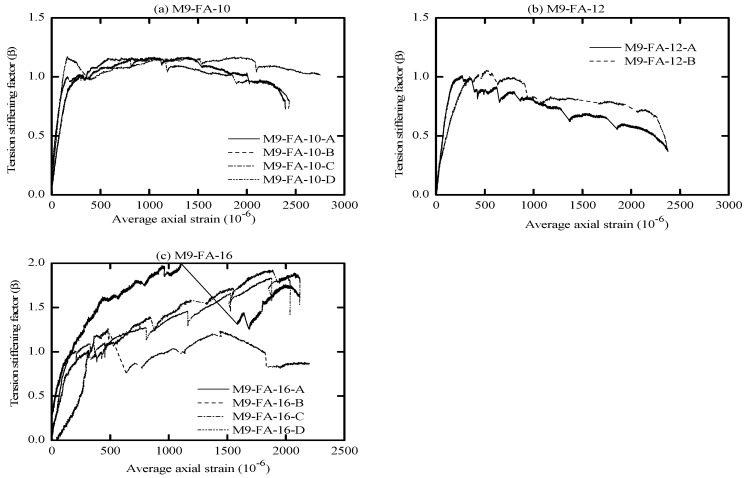
Tension-stiffening factor (β) vs. average axial strain for M9-FA.

**Figure 13:**
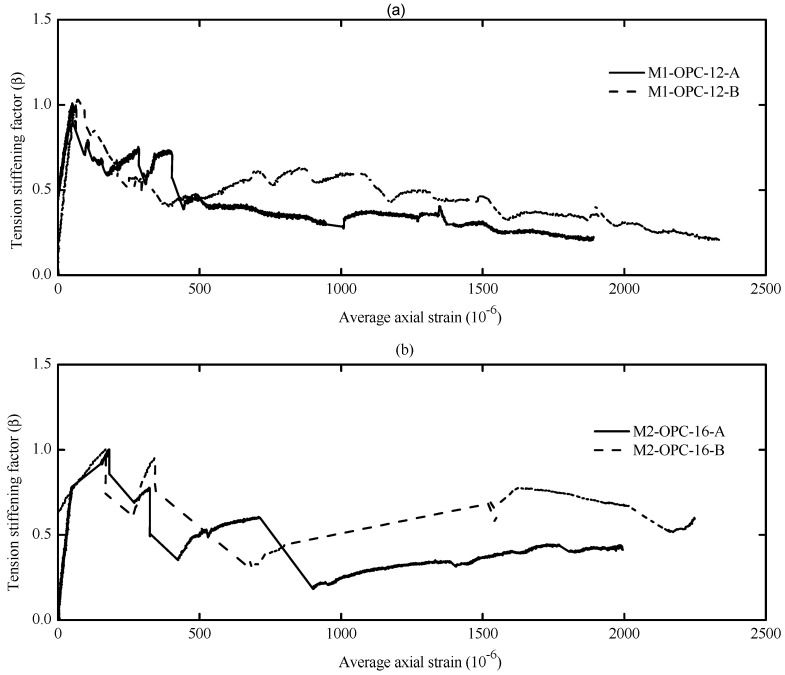
Tension-stiffening factor (β) vs. average axial strain (**a**) M1-OPC-12; (**b**) M2-OPC-16.

The diagrams reported in Figure 10, Figure 11, Figure 12 and Figure 13 illustrate the tension-stiffening factor (β) of the tested AA and OPC prisms. This factor represents the ability of the intact concrete between cracks to carry tensile stresses and contributes to the overall stiffness of the cracked prism. The amount of tensile stresses carried by concrete depends to a large extent on the interaction (bond) between the reinforcement. This factor is often used to describe the tensile behavior of concrete and consider the concrete stress variation in a cracked prism with respect to the cracking stress [53]. It is well known that this factor is a material property for cracked concrete that is independent of the concrete strength and steel bar ratio [27,56].

An important observation from Figure 10, Figure 11 and Figure 12 is that the initiation of the first crack did not reduce the tensile capacity of the AA concrete. However, the cracked AA prisms continued to carry a similar or slightly higher load than their initial cracking load (P_cr_). This trend agrees well with that reported for LCFA prisms in the literature [26,57]. Another significant observation that can be made from the tension-stiffening factor graphs is that AA concrete exhibited better ductile behavior than OPC concrete. This could be attributed to the strain compatibility between concrete and steel even after the crack ignition [51]. However, OPC concrete experienced a gradual reduction in the tension-stiffening factor (β) after the crack formation with less fluctuation than their AA counterparts, as shown in Figure 13. This can be explained as follows: the OPC concrete was initially able to sustain a significant tensile load before cracking, but the cracked OPC concrete lost its ability to carry more stress than the cracked AA concrete. Hence, it can be concluded that the tension-stiffening effect of AA concrete, in general, is more substantial than that of OPC concrete.

The tension-stiffening factor (β) starts to decay at the end of the stabilized cracking stage, as shown in Figure 11a. At the end of this stage, the tensile capacity of concrete is exhausted, especially near the yielding point of reinforcement. This is due to the limited ability of the yielded reinforcement to transfer tension force to the concrete across cracks (bond deterioration), as the steel had undergone a plastic strain when yielded [36,45,51].

By referring to Figure 10, Figure 11 and Figure 12, the maximum value of the tension-stiffening factor for AA concrete was observed to be higher than 1, which is a common value in OPC concrete. Several studies in the literature have also reported that the tension-stiffening factor for other types of AA concrete is higher than the unity value [27,58,59]. The higher value of the tension-stiffening factor is due to the ability of the concrete to carry larger tensile stresses even after cracking and due to the strain-hardening capacity of AA concrete compared to the brittle behavior of OPC concrete [51,60]. After the cracking, the factor degradation was less compared to OPC prisms, and the factor curves stayed constant until near the yielding of the steel. This could be attributed to the improved bond strength of concrete [58]. Thus, further study is required to modify the tension-stiffening factor limit in the code of practice to suit the obtained tension-stiffening factor of AA concrete.

In contrast, the tension-stiffening factor of OPC (plotted in Figure 13) has a maximum value of 1, and that is consistent with the results reported in the literature [26,56]. Therefore, for calculating the crack width by EC2 [61], the value of the tension-stiffening factor suggested is 0.6 for short-term loading, which seems reasonable. After the crack initiation (β = 1), the tension-stiffening factor curves are observed to vanish progressively as the load/strain increases. This is primarily because the OPC concrete sustains large tensile stress before cracking, and this capacity diminishes as soon as it cracks.

From visual observation of the global response diagrams, compressive strength was generally found to be an influencing parameter. This can be observed by comparing the response in Figure 6a and Figure 8a, which have identical reinforcement ratios of 1.42% but different compressive strengths. The specimens with high compressive strength (M5-FA-10) of 41.6 MPa exhibited a better deformation resistance than those with lower compressive strength (M9-FA-10) of 26.4 MPa. This can generally be attributed to the prism with higher compressive strength showing better cracking resistance and improved bond between the concrete and reinforcement. This better bond strength transfers stresses more effectively between the reinforcement and concrete, resulting in a higher stress contribution of concrete [62].

In addition to the compressive strength, the effect of concrete cover to bar diameter (C_c_/d_b_) ratio on the tension stiffening was evaluated by employing the reinforcement of either 10 mm, 12 mm, or 16 mm in diameter. This corresponds to a C_c_/d_b_ ratio of 3.25, 2.63, and 1.84, respectively. Referring to Figure 6, it can be observed that increasing the C_c_/d_b_ ratio slightly improves the elongation resistance of the prisms by adding more stiffness to the bare bar response. This can be explained by the fact that increasing the confinement (C_c_/d_b_ ratio) around the steel bar improves the tensile capacity of the surrounding concrete, which delays the formation of internal cracks [53,62]. Therefore, it can be said that increasing the C_c_/d_b_ ratio enhances the tension stiffening as increasing the confinement means increasing the effective concrete area that carries tensile stresses [56].

### 3.4. Cracking Behavior of AA Concrete

#### 3.4.1. Evolution of Crack Spacing

**Figure 14:**
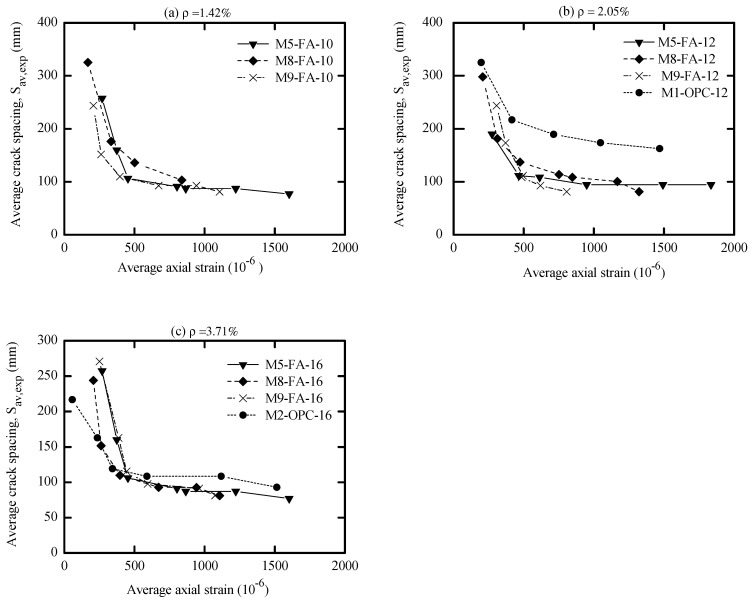
Experimental final crack spacing vs. average axial strain (**a**) 𝜙10; (**b**) 𝜙12; (**c**) 𝜙16.

A significant aspect of the investigation herein concerns crack spacing and its evolution to provide a clear understanding of concrete cracking. Thus, crack spacing results that include the maximum, average, and minimum crack spacing for all specimens were monitored during the test and reported in Supplementary S3 (Table S1). This was completed by measuring the distance between the visible cracks on the concrete surface with the corresponding average axial strain (ɛ_c_) of the prism. Since many building codes formulas evaluate the cracking in terms of average crack spacing (S_av,expr_), the evolution of cracks in the current study followed a similar approach. Thus, the average crack spacing was plotted as a function of the average axial strain, as shown in Figure 14. In addition, the diagrams are plotted to evidence the influence of compressive strength and the associated reinforcement ratio on the final average crack spacing.

Comparing the response of AA specimens with OPC counterparts in Figure 14, one can see that the stabilized cracking stage of former concrete was reached at a slightly similar axial strain to that of OPC specimens. In general, both types of concrete reached the crack-stabilized stage in an average axial strain that varies approximately between 1000 (10^−6^) and 1750 (10^−6^). However, Figure 14 and Supplementary S3 (Table S2) show that the final average crack spacing of AA specimens varies between 72 and 109 mm. Still, OPC control specimens exhibited a larger average crack spacing that varies between 93 and 164 mm. This indicates that the former concrete developed more cracks, resulting in a smaller crack width.

**Figure 15:**
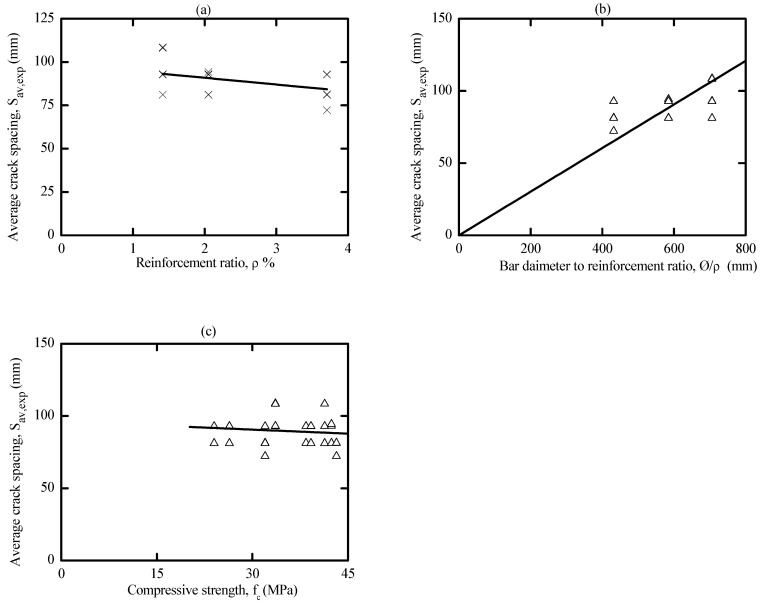
Experimental average crack spacing vs. (**a**) ρ% ratio; (**b**) 𝜙/ρ ratio; (**c**) f_c_.

To better capture the influence of reinforcement ratio (ρ%) on the average crack spacing, the average crack spacing was plotted with respect to the reinforcement ratio (p%) in Figure 15. It is worth mentioning that increasing the reinforcement ratio led to a higher crack number that consequently reduced the average crack spacing. This is because specimens with a high steel ratio had a smaller concrete cross-sectional area, which required a lower force to generate more cracks. This force needs a smaller reinforcement development length that produces a smaller average crack spacing, which agrees well with the conclusions drawn in the literature for OPC and AA concrete [51,53,56,63,64,65].

By referring to Figure 15c, the effects of compressive strength on the average crack spacing were marginal. This suggests that the minimal improvement in the crack spacing due to high-strength concrete only occurs by increasing the tensile strength of the concrete. This is in line with what is well known for OPC, where the estimation of crack spacing is independent of the concrete compressive strength. Therefore, the improvement of crack spacing and, consequently, the crack width with high-strength concrete occurs primarily due to the higher tension-stiffening contribution of concrete that mainly reduces the strain In the member. [56].

Many building codes included a 𝜙/p parameter in estimating the crack spacing of OPC concrete. Thus, it is worth mentioning that Figure 15b describes the influence of this parameter on the average crack spacing of cracked AA concrete, where a possible linear expression between the mean crack spacing and the 𝜙/p parameter could be regressed. It is evident that increasing 𝜙/p ratio produced a higher average crack spacing, which agrees with what is known about OPC concrete.

#### 3.4.2. Final Crack Patterns

**Figure 16:**
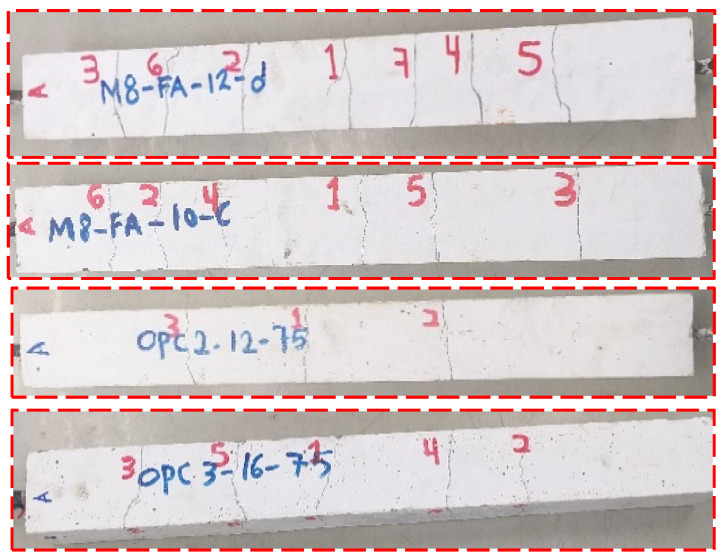
Final crack patterns of the tested specimens.

The crack patterns of all tested specimens are given in Supplementary S1 (Figure S1) together with the corresponding crack numbers and locations. As shown in Figure 16, crack development was not uniform on all sides of the tested specimens. The first traverse (primary) cracks appeared near the middle portion of the specimen for both AA concrete and OPC prisms. Increasing the load resulted in widening the first crack and forming additional cracks. In some AA prisms, more than one crack appeared simultaneously, each crack occurring near the edge on the opposite side. This indicates a uniform distribution of tensile capacity along with the member [50]. Splitting cracks, however, did not form in any specimens, unlike the LCFA alkali-activated concrete prism reported in the literature [26]. This could be due to the high bond strength of AA concrete and the adequate concrete cover around the steel bar [51]. In general, it is worth mentioning that the final crack spacing of AA specimens was substantially smaller than those of OPC counterparts. Therefore, it can be said that AA concrete gave a slightly better cracking performance compared to OPC concrete.

#### 3.4.3. Growth of Crack Width

**Figure 17:**
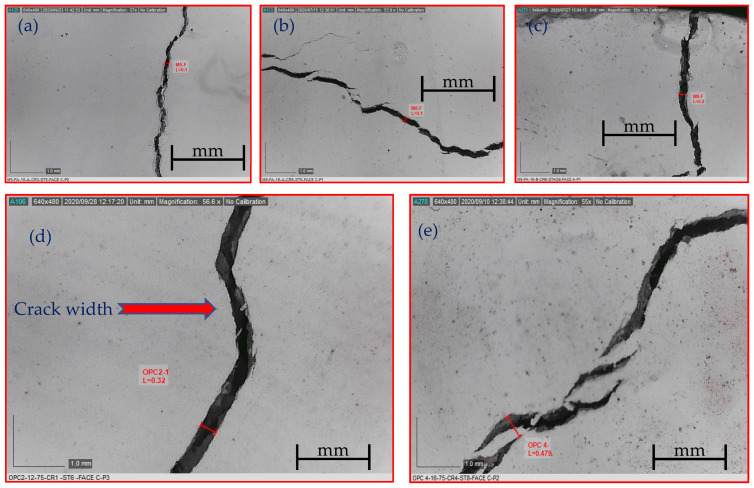
Sample of crack width measurement for the following specimens: (**a**) M5-FA-10 (A); (**b**) M8-FA-16 (A); (**c**) M9-FA-16 (B); (**d**) M1-OPC-12 (B); (**e**) M1-OPC-16 (B).

The crack width monitoring began upon crack initiation and continued whenever a new crack appeared on the concrete surface. After the crack pattern stabilized, the monitoring process was carried out at different loading intervals during the test with an increment that varied between 10 and 15 kN. An example of crack width monitoring is given in Figure 17 by taking a picture that was used to extract the crack width. The crack evolution for each specimen, including the maximum, average, and minimum crack widths, along with the corresponding normalized steel stress to yield stress (σ/Fy), are presented in Supplementary S3 (Table S4) and represented graphically as a function of normalized steel stress in Supplementary S2 (Figures S2–S11). It is worth mentioning that the evolution of the average crack width relies on the number of cracks formed at a specific load. In this regard, the formation of multi-cracks at a particular load resulted in a uniform distribution of steel strain on the newly formed cracks. Thus, the average crack width may not necessarily increase [26].

By referring to Supplementary S2 (Figures S2–S10), it can be seen that specimens with a reinforcement ratio of 1.42% developed cracks at a higher σ/Fy ratio of about 0.2 compared to the samples with a reinforcement ratio of 3.71%. This could be attributed to the significant tensile stresses carried by concrete before the initiation of cracks, as reported in the literature [26]. However, it can be observed that specimens with a higher reinforcement ratio (3.71%) provide a narrower crack width compared to the specimens with a lower reinforcement ratio. One possible explanation is that reducing the reinforcement ratio means a higher concrete cover that deters the internal cracks from propagating to the outer surface, resulting in a smaller number of cracks [50,65]. One more significant observation that can be made from the graphs is that for the same reinforcement ratio, specimens with higher compressive strength gave a narrower crack width, indicating the higher contribution of tension-stiffening effects that reduce member strain [56].

As given in Supplementary S2 (Figure S11), the OPC control sample (i.e., M1-OPC) developed fewer cracks with a bigger opening compared to its AA specimen’s counterpart. This is because the intact OPC concrete between cracks did not contribute significantly to the tensile capacity, resulting in a higher elongation of the steel bar at the crack location and hence a wider crack forming. This conclusion agrees well with the findings reported in the literature [26]. Therefore, it can be generally mentioned that AA specimens had a substantially smaller crack width than those OPC counterparts due to the higher tension-stiffening behavior of the former concrete.

**Figure 18:**
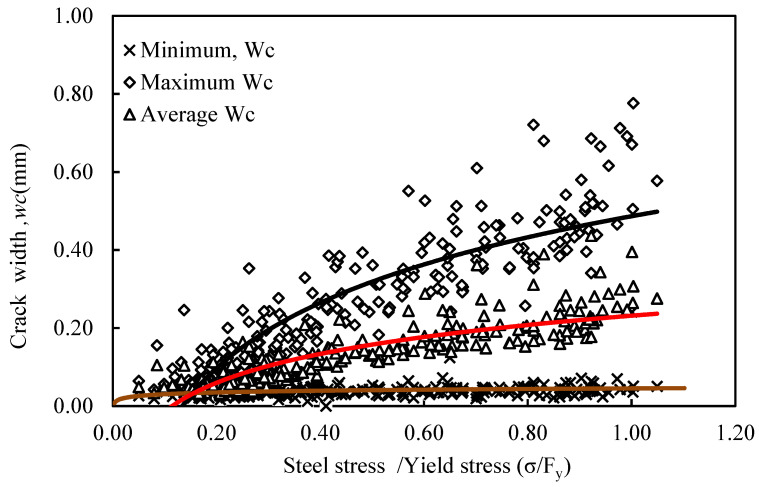
Crack width growth versus steel stress/yield stress (σ/Fy).

The crack width growth results shown in Supplementary S2 (Figures S2–S10) were described in a mathematical expression logarithmic form for each specimen as a function of normalized steel stress and reported in Supplementary S3 (Table S5). In addition, more general mathematical expressions were developed for the entire specimens tested in the present study (Figure 18) for the minimum, average, and maximum crack. These predictive models are presented in Equations (5)–(7).

(5) $$w_{c,Max}=0.24IN\left(\;\frac{\sigma_{s}}{y_{y}}\right)+0.49,\;R^{2}=\;0.76\;\;$$

(6) $$w_{c,Averg}=0.10IN\left(\;\frac{\sigma_{s}}{y_{y}}\right)+0.22,\;R^{2}=0.68\;\;$$

(7) $$w_{c,Min}=0.01IN\left(\;\frac{\sigma_{s}}{y_{y}}\right)+0.05,\;R^{2}=0.05\;$$

### 3.5. Comparison of Experimental Cracking Response with Code Provisions

This section thoroughly discussed the verification of the experimental crack spacing and width through comparisons with that computed using the OPC codes formula. Regressed models were also proposed, and their predictions were statically compared with OPC codes.

#### 3.5.1. Comparison of Experimental Crack Spacing with Code Provisions

**Figure 19:**
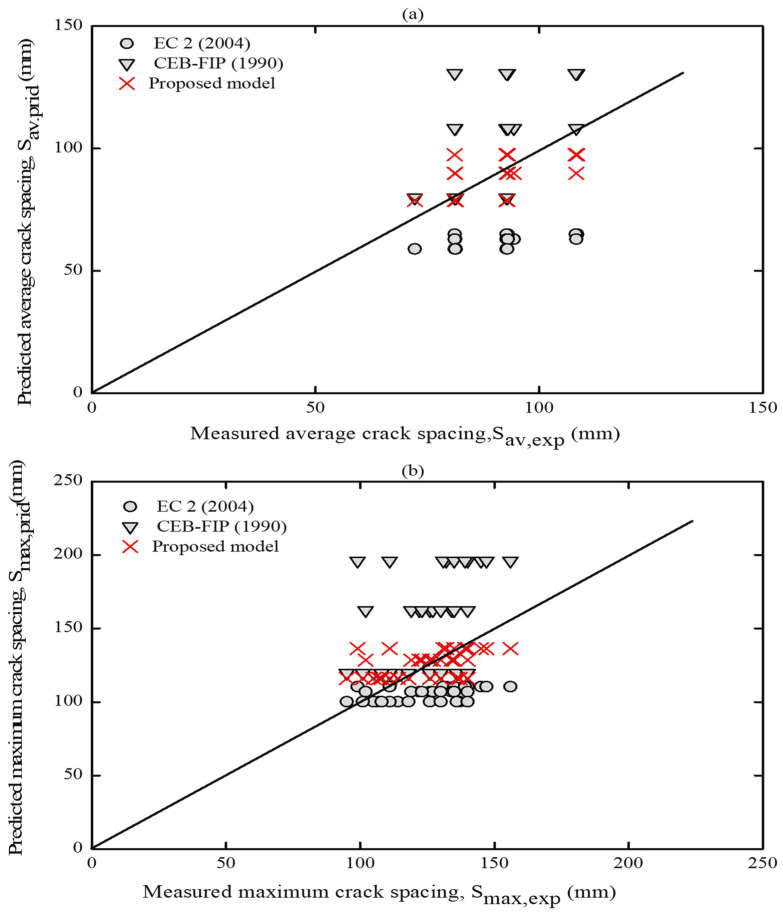
Predictions by codes and proposed model vs. experimental (**a**) average and (**b**) maximum crack spacing.

One of the main goals of the current work is to check the applicability of OPC concrete approaches in various codes’ provisions by comparing the estimated values of the crack spacing with the experimental results of the present study. The design codes considered were EC2 [61] and MC90 [66], and their formulations are provided in Table 5. The serviceability analyses described in these codes of practice are intended for the stabilized cracking stage [51]. Thus, a comparison was made between the experimental and predicted average and maximum crack spacing values at the crack stabilized stage, which are given in Supplementary S3 (Table S2) and graphically plotted in Figure 19. The predicted crack spacing is marked with different legends according to the code of practice employed to avoid overlapping in the data, which is more likely to occur.

By referring to Figure 19, it can be seen that the average crack spacing predictions by EC2 [61] formula fell slightly under the equality line; however, the predicted maximum crack spacing results lie close to the equality line. This indicates that the predicted values agree well with the experimental maximum crack spacing values. One possible cause of this agreement could be due to the actual concrete cover (c) and the high bond behavior considered in these provisions (assuming k_1_ = 0.8). In contrast, the predicted values of the average and maximum crack spacing by CEB-FIP [66] provisions are mostly on the top side of the equality line. This suggests that this code overestimated the maxim crack spacing, especially at a lower reinforcement ratio (ρ = 1.47%), as shown in Supplementary S3 (Table S2).

The discrepancy in the predicted values between both design codes can be attributed to considering the concrete cover, C_c_, bond factors, k_1_, and loading factor, k_2_, in the EC2 [61] formulation. As a result, it gave a better estimate of crack spacing. Meanwhile, an empirical model to estimate the average and maximum crack spacing as a function of 𝜙/ρ was derived based on the EC2 [61] formula and given in Table 5, respectively. The estimated average and maximum crack spacing by the proposed models are presented in Supplementary S3 (Table S2) and graphically compared to the experimental data as shown in Figure 19. This comparison revealed a better good agreement than the earlier two provisions, as statistically shown in Table 6.

#### 3.5.2. Comparison of the Experimental Crack width with Code Provisions

The expressions of EC2 [61] and CEB-FIP [66] provided in Table 7 directly predict the maximum crack width (w_max_) based on the maximum crack spacing. In contrast, the ACI 224R [67] approach can directly predict the crack width without estimating the cracking space, as shown in the equations in Table 7.

**Figure 20:**
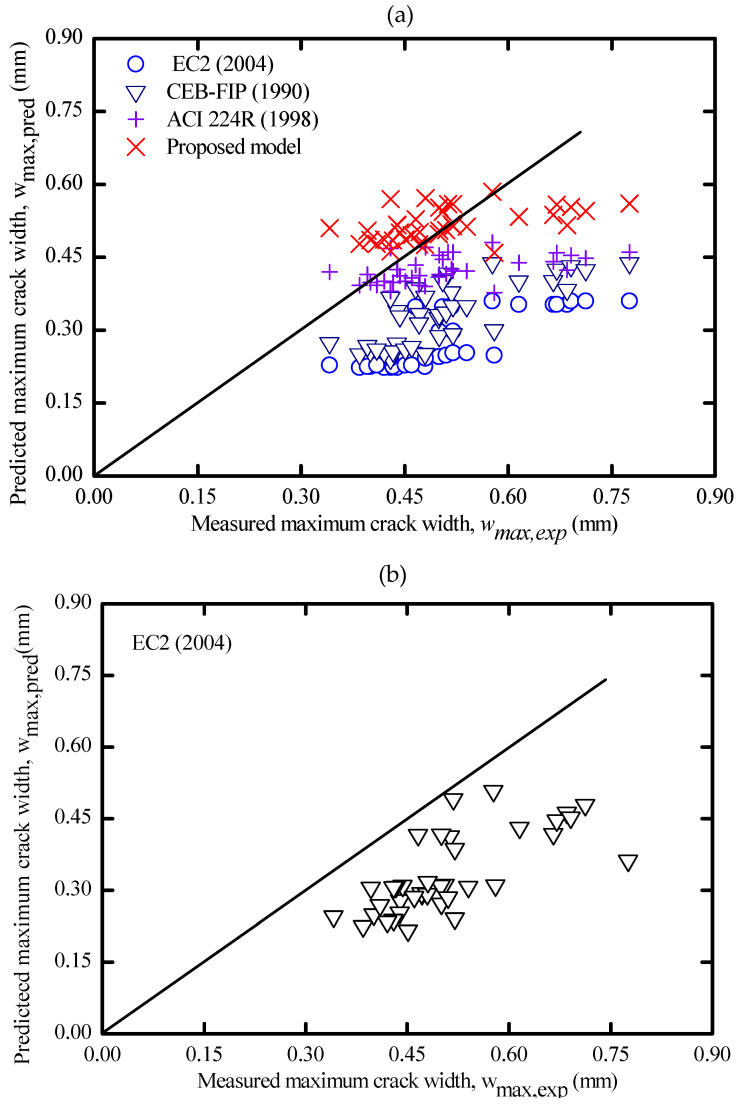
(**a**) Predictions by codes and proposed model vs. experimental maximum crack width and (**b**) Predictions by codes vs. experimental maximum crack width.

An overall comparison between the experimental and predicted maximum crack widths is summarized in Supplementary S3 (Table S3) and plotted in Figure 20a,b. Unlike the case of crack-spacing predictions, it can be observed that in Figure 20a, the predicted maximum crack width by CEB-FIP [66] formulation lies closely to the equality line. This indicates that the estimated values agree with the experimental results of maximum crack width with minimal variations. Considering the random nature of cracking, this slight variation may be acceptable, and thus, the predictions of maximum crack width by CEB-FIP [66] are generally proven to be applicable.

Figure 20a shows that the predicted results by the EC2 [61] formula were most of the time below the equity line even when the bond coefficient for high bond bars is considered (k1 = 0.8). This indicates that the EC2 [61] approach tends to underestimate the maximum crack width despite using the maximum predicted crack spacing to calculate crack width. In some cases, the EC [61] formula predicted half of the experimental maximum crack width. This might be because the EC2 formula was mainly developed to evaluate the crack width of the flexural member rather than the tensile member. Therefore, in Figure 20b, a second comparison was performed again between the experimental and new predicted maximum crack width, which was computed directly based on the experimental maximum crack spacing of each AA specimen (S_max,exp_) rather than using the theoretical maximum crack spacing (S_av,pred_). An improvement in code predictions has been obtained, and therefore, the crack width code formulas of EC2 [61] have been proven to be applicable despite the slight inconsistency in predictions.

Comparing the experimental and the prediction values of the maximum crack width by the ACI 224R [67] provision given in Figure 20a, it is clearly seen that the calculated maximum crack width is better than the previously mentioned provisions. The slight underestimation between the theoretical and experimental crack width is likely due to the crack width formula being intended for the flexural members with narrower crack widths than tensile members. Since ACI 224R [67] provided better predictions of crack width, a mathematical model to predict the maximum crack width was derived for AA concrete in Table 7. The prediction of this model is graphically plotted in Figure 20a and statistically compared with other provisions in Table 8.

## 4. Conclusions

Alkali-activated concrete is a sustainable construction material, and understanding its tension stiffening and crack spacing and width is of critical importance. These properties are essential indicators of the concrete suitability for structural applications, particularly at service limit states. Therefore, the current work aimed to investigate the tension stiffening and cracking performance of AA concrete by considering a wide range of compressive strengths and C_c_/d_b_ ratios. Following this, the applicability of OPC concrete approaches in various codes of practice to predict the crack spacing and width of this concrete has been examined. Based on the results of the present work, these conclusions were drawn.

1. Both AA and OPC concrete prisms developed slightly similar axial cracking force (N_cr_). Both concrete types also have a global response consisting of three stages: elastic, cracking, and stabilized. However, the OPC prisms experienced a brittle cracking mechanism, resulting in a sudden drop in the load–strain curves at the crack location.
2. AA concrete prisms developed more than one crack simultaneously, suggesting that the concrete tensile strength was more uniform in AA specimens than in OPC specimens. The tension-stiffening factor (β) of AA concrete exhibited better ductile behavior than OPC concrete due to the strain compatibility between concrete and steel even after the crack ignition. In contrast, OPC concrete experienced a gradual reduction in the tension-stiffening factor (β) after the crack formation.
3. Compressive strength was generally found to be an influencing parameter in the global response of tested prisms because it improves the cracking resistance and bond between concrete and reinforcement. In addition, increasing the confinement (C_c_/d_b_ ratio) around the steel bar improves the tensile capacity of the surrounding concrete, which delays the formation of internal cracks and, consequently, enhances the tension stiffening of AA concrete.
4. In contrast to AA concrete, the OPC control sample developed fewer cracks with a bigger opening, as the intact OPC concrete between cracks did not contribute significantly to the tensile capacity, resulting in a higher elongation of the steel bar at the crack location and.
5. The predicted crack spacing by EC2 was almost in line with that obtained experimentally. This agreement could be due to considering the actual concrete cover (c) and the high bond behavior considered. In contrast, CEB-FIP predictions of crack spacing are mostly on the top side of the equality line. In addition, code provisions tend to underestimate the maximum crack width, especially EC2 [62], and this was because the codes formula was intended for flexural members with narrower crack widths than tensile members.

## Figures and Tables

**Table 1:** Chemical properties of FA by mass percentage.

Compounds	Fe_2_O_3_	SiO_2_	AL_2_O_3_	CaO	MgO	SO_3_	CI	TiO_2_	MnO	K_2_O	LOI
Mass (%)	18.95	32.3	16.4	19.1	7.6	2	0.13	0.85	0.18	1.6	3.2

Note: LOI denotes loss of ignition of fly ash.

**Table 2:** Mixtures proportions of AA and OPC concrete.

Mix ID.		Quantity (kg/m^3^)							
	AA: FA	CA	FAG	OPC	FA	NaOH	Na_2_SiO_3_	Borax	Water
M5-FA	0.34	1060	707	-	473	64	96	2.37	74
M8-FA	0.37	1060	707	-	430	64	96	2.15	74
M9-FA	0.40	1060	707	-	398	64	96	1.99	74
M1-OPC	-	1375	550	325	-	-	-	-	188
M2-OPC	-	1350	528	375	-	-	-	-	188

Note: AA: FA = Alkaline Activator-to-Fly Ash Ratio, CA = Coarse Aggregate, FAG = Fine Aggregate.

**Table 3:** Mechanical properties of the steel bars.

𝜙 (mm)	A_s_ (mm^2^)	*F_y_* (MPa)	*ɛ*_y_	*F_u_* (MPa)	*ɛ_u_*
10	79	573	0.0037	622	0.040
12	113	502	0.0027	576	0.054
16	201	526	0.0025	618	0.052

𝜙 = Steel bar diameter, A_s_ = Steel bar cross-section area. *F_y_* = Yield strength, *ɛ_y_* = Yield strain, *F_u_* = Ultimate strength, *ɛ_u_* = Ultimate strain.

**Table 4:** Test results of tension stiffening for AA and OPC concrete specimens.

Specimen	*F_c_*	C_c_/d_b_	ρ	N_cr_	S.D	ε_cr_	ε_sb,cr_	N_sb,cr_	P_cr_	F_cr_
ID	(MPa)	ratio	(%)	(kN)	(kN)	(10^−6^)	(10^−6^)	(kN)	(kN)	(MPa)
M5–FA 10 (A)	41.6	3.25	1.42	9.56	1.18	273	865	4.29	5.27	0.95
M5–FA 10 (B)		3.25		8.65		189	866	2.97	5.68	1.03
M5–FA 10 (C)		3.25		11.31		310	725	4.88	6.43	1.16
M5–FA 10 (D)		3.25		10.65		239	682	3.79	6.9	1.24
M5–FA 12 (A)	42.4	2.63	2.05	11.69	1.68	205	508	4.64	7.05	1.28
M5–FA 12 (B)		2.63		13.37		261	624	5.9	7.47	1.36
M5–FA 12 (C)		2.63		15.56		352	768	7.96	7.6	1.38
M5–FA 12 (D)		2.63		12.41		282	602	6.38	6.03	1.1
M5–FA 16 (A)	43.2	1.84	3.71	15.69	2.07	241	474	9.69	6	1.11
M5–FA 16 (B)		1.84		19.84		329	502	13.23	6.61	1.22
M5–FA 16 (C)		1.84		16.26		264	489	10.62	5.64	1.04
M5–FA 16 (D)		1.84		19.19		245	506	9.85	9.34	1.72
M8–FA 10 (A)	33.6	3.25	1.42	9.055	0.15	164	577	2.59	6.49	1.17
M8–FA 10 (B)		3.25		8.79		113	731	1.8	7	1.26
M8–FA 10 (C)		3.25		9.07		199	594	3.13	5.94	1.07
M8–FA 10 (D)		3.25		8.81		198	601	3.11	5.7	1.03
M8–FA 12 (A)	38.4	2.63	2.05	9.87	1.80	232	578	5.25	4.62	0.84
M8–FA 12 (B)		2.63		8.3		200	425	4.52	3.78	0.69
M8–FA 12 (C)		2.63		12.34		261	574	5.9	6.44	1.17
M8–FA 12 (D)		2.63		11.53		250	597	5.65	5.88	1.07
M8–FA 16 (A)	39.2	1.84	3.71	10.58	3.52	123	253	4.95	5.63	1.04
M8–FA 16 (B)		1.84		17.73		298	714	11.98	5.75	1.06
M8–FA 16 (C)		1.84		16.46		171	452	6.88	9.58	1.77
M8–FA 16 (D)		1.84		18.2		235	473	9.45	8.75	1.61
M9–FA 10 (A)	26.4	3.25	1.42	9.25	0.80	345	619	5.42	3.83	0.69
M9–FA 10 (B)		3.25		9.03		292	666	4.59	4.44	0.8
M9–FA 10 (C)		3.25		9.19		353	707	5.54	3.65	0.66
M9–FA 10 (D)		3.25		7.57		166	558	2.61	4.96	0.89
M9–FA 12 (C)	24	2.63	2.05	11.75	0.76	270	551	6.11	5.64	1.02
M9–FA 12 (D)		2.63		12.83		343	622	7.76	5.07	0.92
M9–FA 16 (A)	32	1.84	3.71	12.35	5.13	164	307	6.59	5.76	1.06
M9–FA 16 (B)		1.84		24.87		320	722	12.9	11.97	2.2
M9–FA 16 (C)		1.84		18.66		315	516	12.67	5.99	1.1
M9–FA 16 (D)		1.84		17.79		203	535	8.16	9.63	1.77
M1-OPC-12 (A)	28	2.63	2.05	10.49	5.49	104	599	2.35	8.14	1.47
M1-OPC-12 (B)		2.63		18.26		141	808	2.86	15.4	2.76
M2-OPC-16 (A)	35	1.84	3.71	10.1	2.55	52	401	2.076	8	1.48
M2-OPC-16 (B)		1.84		13.7	63		348	2.53	11.18	2.06

Notation: S.D = standard deviation for the cracking load (N_cr_).

**Table 5:** Mean and maximum crack spacing provisions along with the proposed model.

Parameters	Approach	Expression
Average crack spacing (S_rm_)	EC2 [61]	$2c+0.25\kappa_{1}\kappa_{2}\frac{\phi }{\begin{array}{c} \rho_{eff} \end{array}}$
	CEB-FIB [66]	$\frac{2}{\;3}\times \frac{\phi }{3.6\begin{array}{c} \rho_{eff} \end{array}}$
	Proposed model	$2c+0.046\;\frac{\phi }{\begin{array}{c} \rho_{eff} \end{array}}$
Maximum crack spacing (S_max_)	EC2 [61]	$3.4c+0.425\kappa_{1}\kappa_{2}\frac{\phi }{\begin{array}{c} \rho_{eff} \end{array}}$
	CEB-FIB [66]	$\frac{\phi }{3.6\begin{array}{c} \rho_{eff} \end{array}}$
	Proposed model	$3.41c+0.036\;\frac{\phi }{\begin{array}{c} \rho_{eff} \end{array}}$

Notation: *c*: concrete cover; *κ*_1_: 0.8 for ribbed steel bar and or 1.6 for plain steel bar; *κ*_2_: 0.5 for flexural loading and 1 for pure tension loading; 𝜙: steel bar diameter; *ρ_eff_*_:_ steel area to the effective area of the concrete in tension.

**Table 6:** Comparison of experimental average and maximum crack spacing results with the predictive models.

Statistics	Average Crack Spacing (mm)			Maximum Crack Spacing (mm)		
	EC2 [61]	CEB-FIB [66]	P	EC2 [61]	CEB-FIB [66]	P
Mean	0.70	1.18	1.0	0.85	1.25	1.01
S.D	0.06	0.21	0.10	0.10	0.24	0.12
C.I	0.02	0.07	0.03	0.03	0.08	0.04
C.O.V	0.09	0.18	0.10	0.12	0.19	0.12

Notation: P = proposed model; S.D = standard deviation; C.I = confidence interval; C.O.V = coefficient of variation.

**Table 7:** Formulations for crack width considered in the present study.

Parameters	Approach	Formula
Maximum crack width	EC2 [61]	$w_{K}=S_{max}\left(\varepsilon_{sm}-\varepsilon_{cm}\right)$ $\varepsilon_{sm}-\varepsilon_{cm}=\varepsilon_{s}-\kappa_{t}\left[\frac{f_{ctm}A_{c,eff}}{E_{s}A_{s}}+\frac{f_{ctm}}{E_{c}}\;\;\right]$
	CEB-FIP [66]	$w_{k}={ᶩ}_{s,max}\left(\varepsilon_{sm}-\varepsilon_{cm}-\varepsilon_{cs}\right)$ $\varepsilon_{sm}-\varepsilon_{cm}=\varepsilon_{s}-k_{t}\left[\frac{f_{ctm}\left(1+\alpha_{e}\;\rho \right)}{E_{s}\;\rho }\;\;\right]$
	ACI 224R [67]	$w_{max}=0.0145\sigma_{s}\;{\left(d_{c}\;A\;\right)}^{0.33}\times 10^{-3}$
	Proposed Model	$w_{max}=0.0176\sigma_{s}\;{\left(d_{c}\;A\;\right)}^{0.33}\times 10^{-3}$

Notation: *W_κ_*: the characteristic crack width; ɛ*_sm_* − ɛ*_cm_*: the difference between steel mean strain and concrete mean strain; ɛ*_cs_*: the concrete shrinkage strain (neglected); *f_ctm_*; the mean tensile strength of concrete (0.3F_ck_^2/3^); *E_s_*: the modulus of elasticity of reinforcement; *E_c_*: the modulus of elasticity of concrete (22 (F_cm_/10)^0.3^); ᶩ*_s,max_*: the maximum crack spacing; *A_s_*: the area of steel bar; *A_c_,_eff_*: the effective area of concrete in tension; ɛ*_s_*: the strain in the reinforcing bar at the cracked section at the actual load (ɛ*_s_* = (P/*E*_s_*A_s_*)); *k_t_*: 0.6 for short-term loading and 0.4 for long-term loading; *σ_s_*: the stress in the steel bar at the cracked section; *d_c_*: the concrete cover to the center of the steel bar; *A*: the effective concrete cross-section; *α_e_*: the modular ratio (*E_c_*/*E_s_*).

**Table 8:** Comparison of experimental maximum crack width results with the predictive models.

Statistics	Maximum Crack Width (mm)			
	EC2 [61]	CEB-FIP [66]	ACI 224R [67]	P
Mean	0.65	0.66	0.86	1.05
S.D	0.11	0.09	0.14	0.17
C.I	0.04	0.03	0.05	0.06
C.O.V	0.17	0.14	0.16	0.16

Notation: P = proposed model; S.D = standard deviation; C.I = confidence interval; C.O.V = coefficient of variation.

## Supplementary Materials

The following supporting information can be downloaded at: https://www.mdpi.com/article/10.3390/ma16114120/s1, Supplementary S1: Final crack pattern of all specimens; Supplementary S2: Crack width vs. steel stress/yield stress; Supplementary S3: Cracking results.
