# Supplementary material for: Tension Stiffening and Cracking Behavior of Axially Loaded Alkali-Activated Concrete
Source: Materials (Basel). 2023 May 31;16(11):4120. doi: 10.3390/ma16114120 (PMC10254435; doi:10.3390/ma16114120)
Supplement: Supplementary file 1 [file materials-16-04120-s001.zip › materials-2382089-supplementary.pdf]

Supplementary S1 **Modes of failure of prisms**

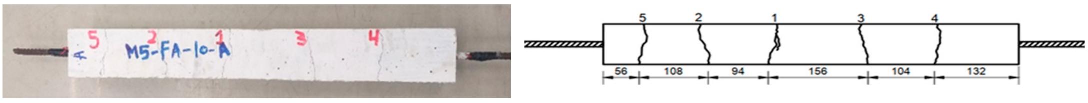

M5-FA-10-(A)

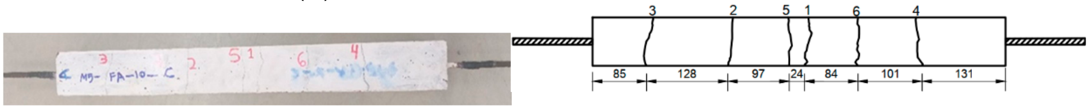

M5-FA-10-(B)

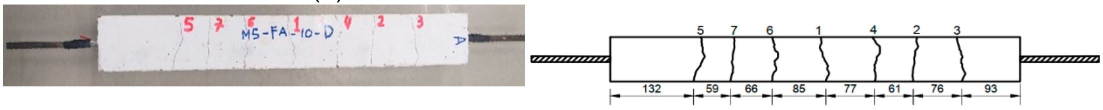

M5-FA-10-(C)

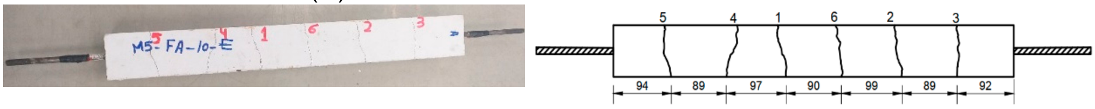

M5-FA-10-(D)

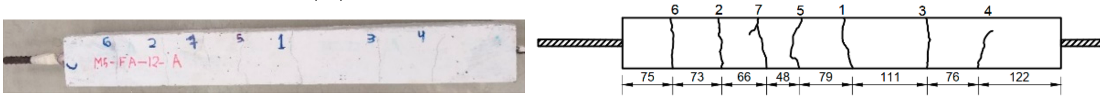

M5-FA-12-(A)

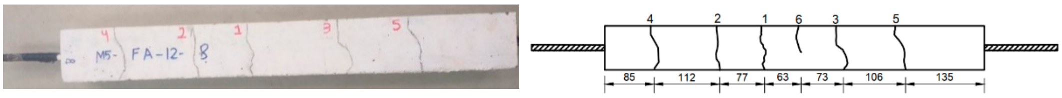

M5-FA-12-(B)

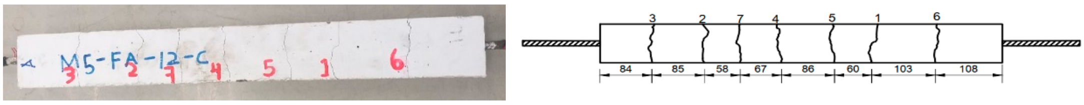

M5-FA-12-(C)

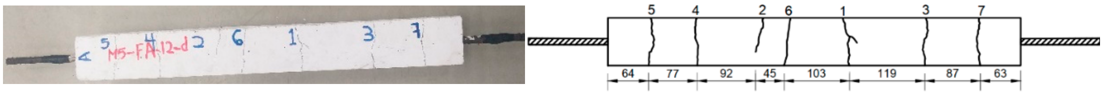

M5-FA-12-(D)

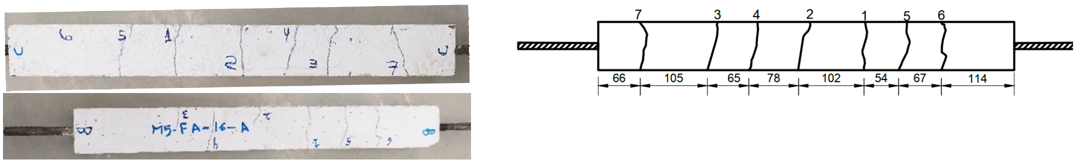

M5-FA-16-(A)

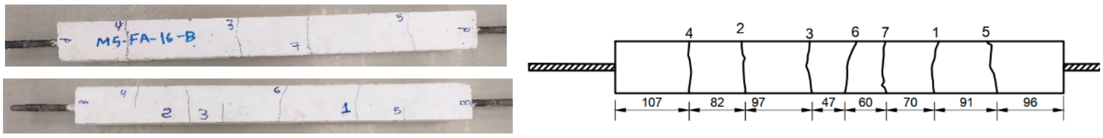

M5-FA-16-(B)

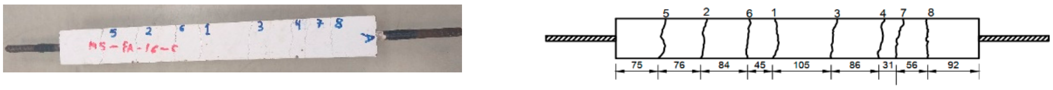

M5-FA-16-(C)

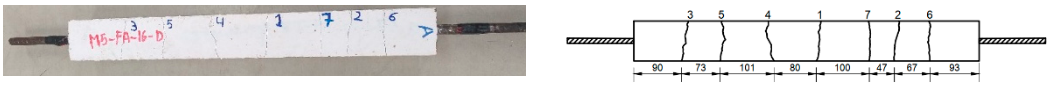

M5-FA-16-(D)

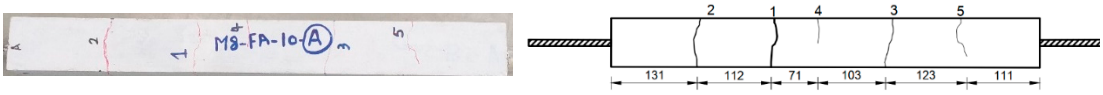

M8-FA-10-(A)

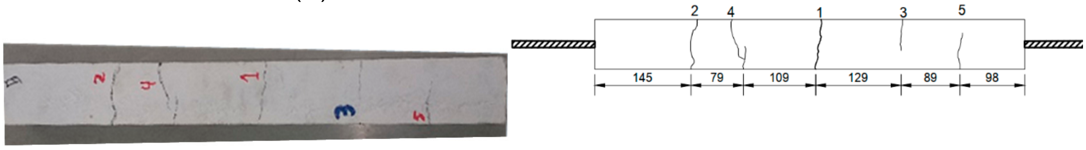

M8-FA-10-(B)

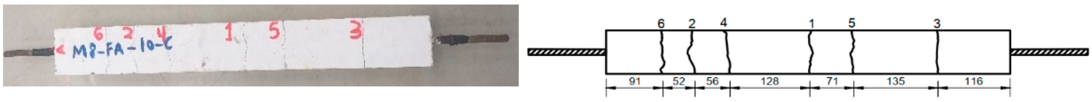

M8-FA-10-(C)

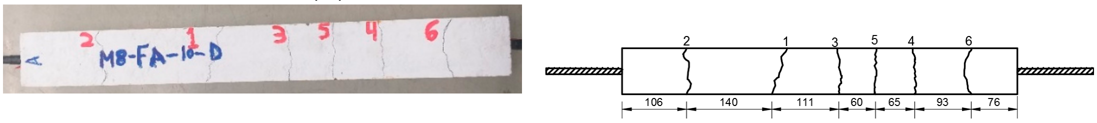

M8-FA-10-(D)

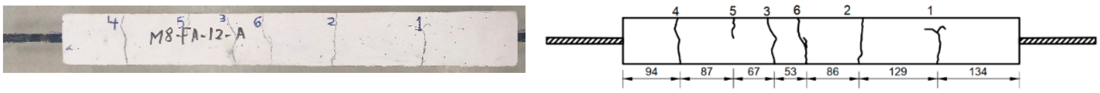

M8-FA-12-(A)

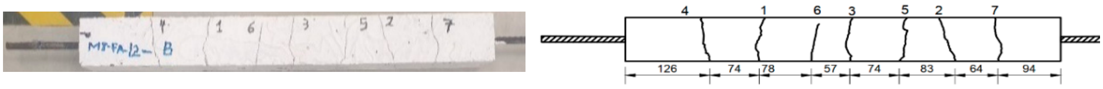

M8-FA-12-(B)

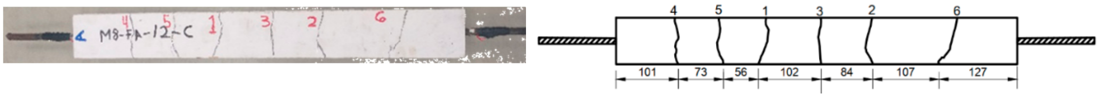

M8-FA-12-(C)

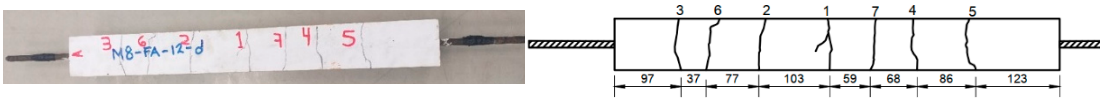

M8-FA-12-(D)

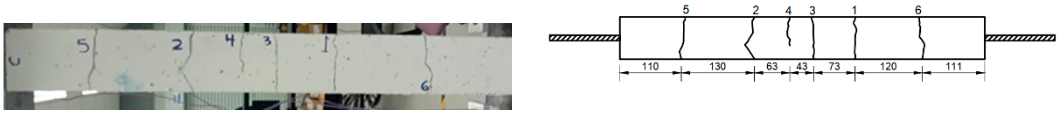

M8-FA-16-(A)

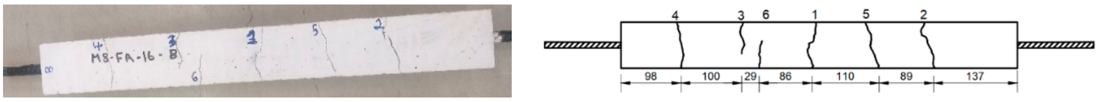

M8-FA-16-(B)

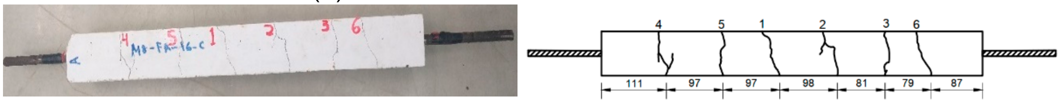

M8-FA-16-(C)

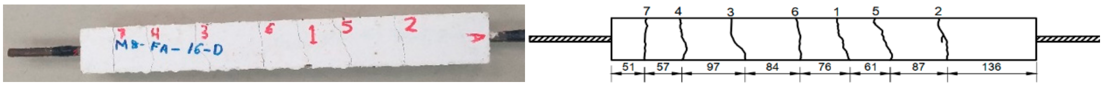

M8-FA-16-(D)

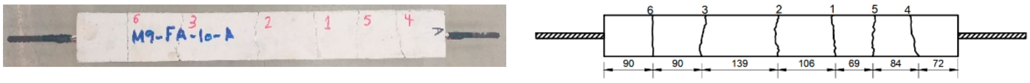

M9-FA-10-(A)

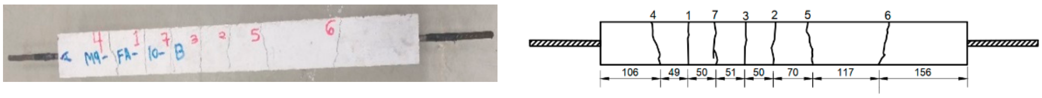

M9-FA-10-(B)

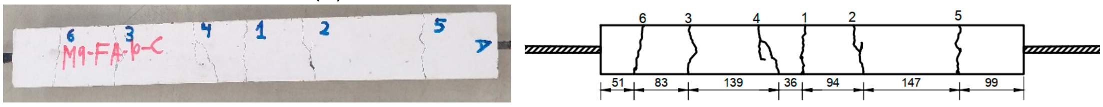

M9-FA-10-(C)

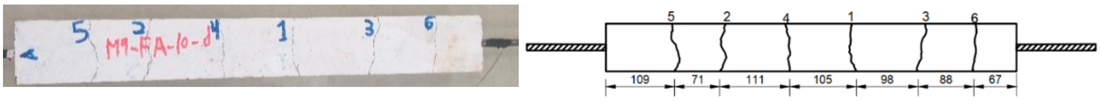

M9-FA-10-(D)

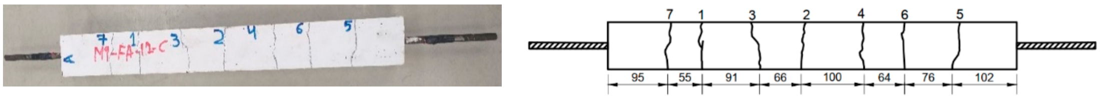

M9-FA-12-(A)

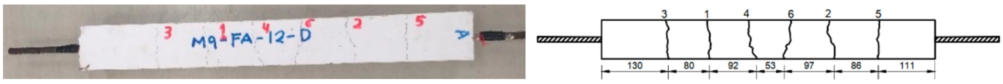

M9-FA-12-(B)

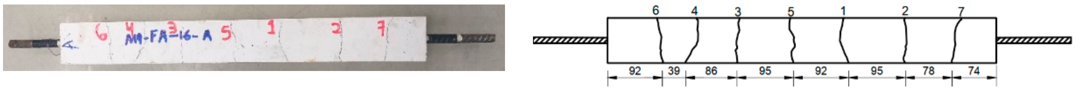

M9-FA-16-(A)

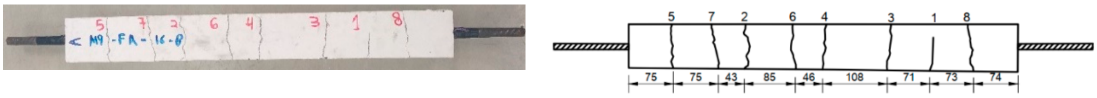

M9-FA-16-(B)

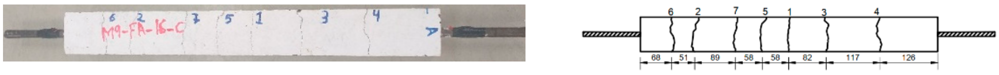

M9-FA-16-(C)

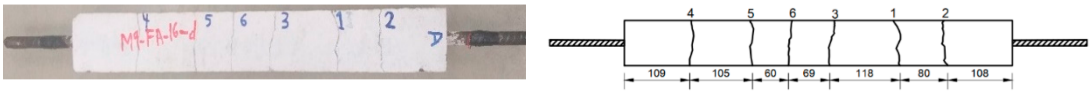

M9-FA-16-(D)

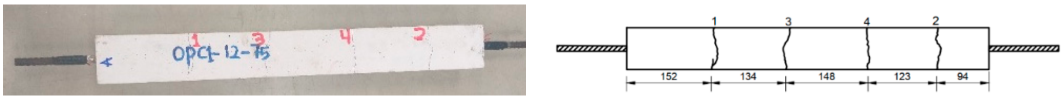

M1-OPC-12-(A)

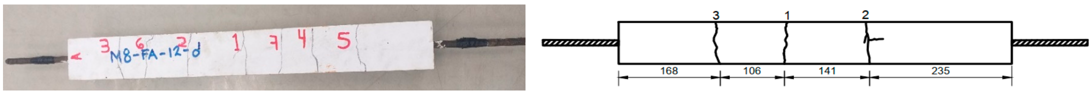

M1-OPC-12-(B)

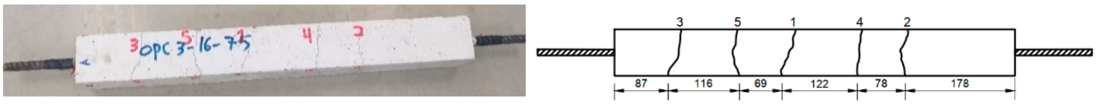

M2-OPC-16-(A)

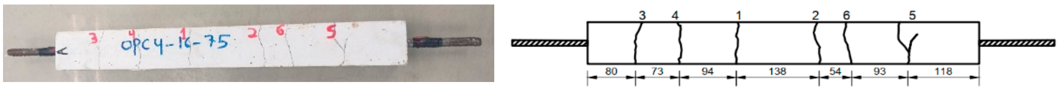

M2-OPC-16-(B)

Figure s1 Final crack pattern of all specimens.

Supplementary S2 Crack widths versus steel stress/yield stress.

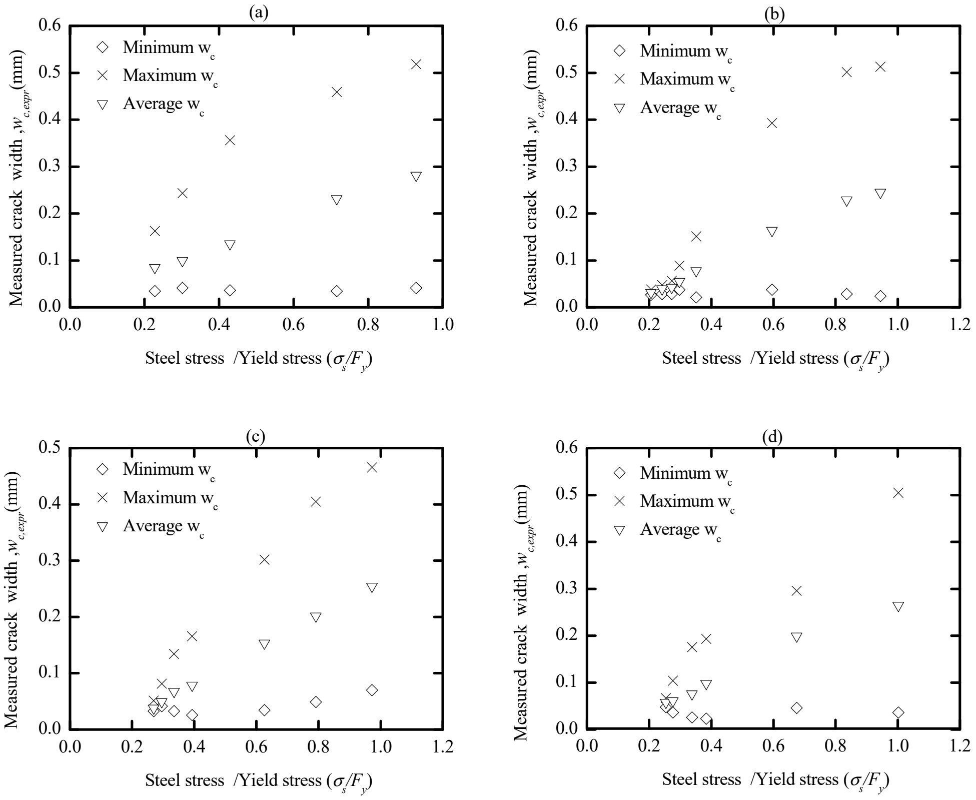

Figure S2 Crack width vs. steel stress/yield stress (a) M5-FA-10 (A); (b) M5-FA-10 (B); (c) M5-FA-10(C), (d) M5-FA-10(D).

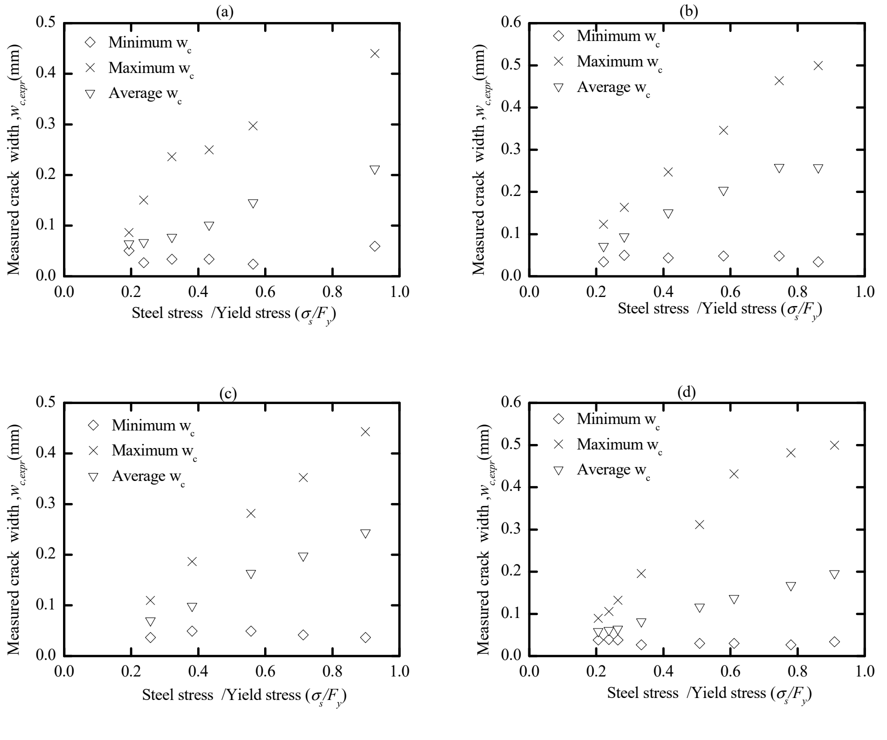

Figure S3 Crack width vs. steel stress/yield stress (a) M5-FA-12 (A); (b) M5-FA-12 (B); (c) M5-FA-12(C), (d) M5-FA-12(D).

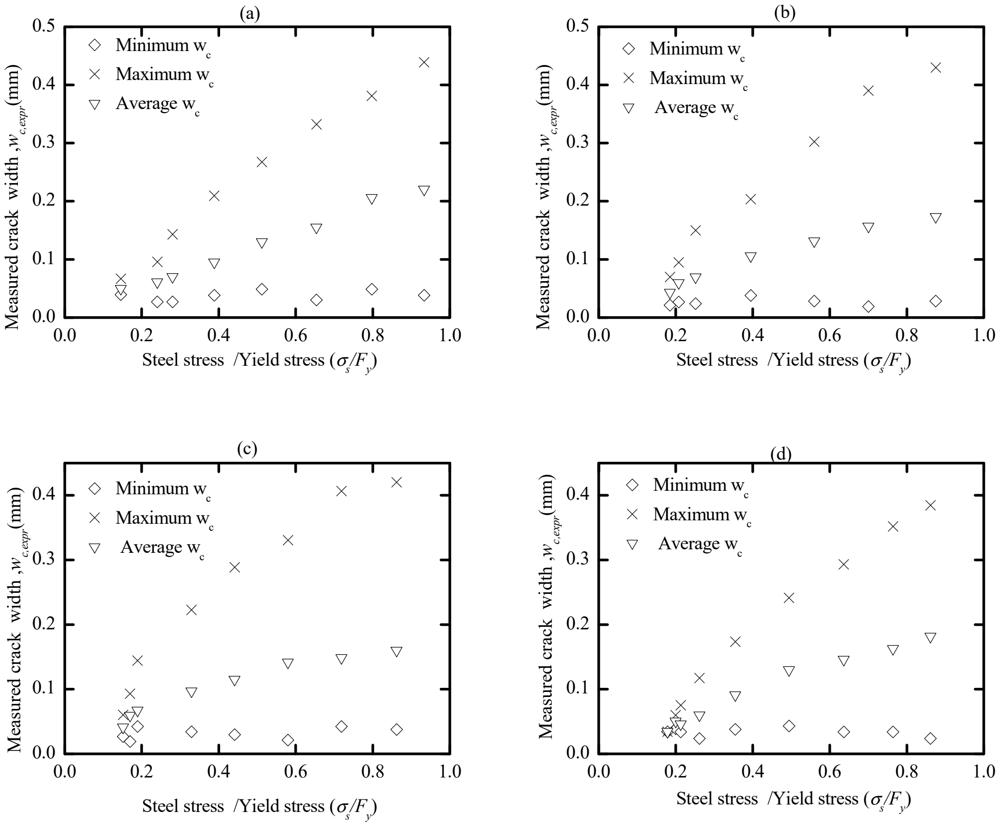

Figure S4 Crack width vs. steel stress/yield stress (a) M5-FA-16 (A); (b) M5-FA-16 (B); (c) M5-FA-16 (C), (d) M5-FA-16 (D).

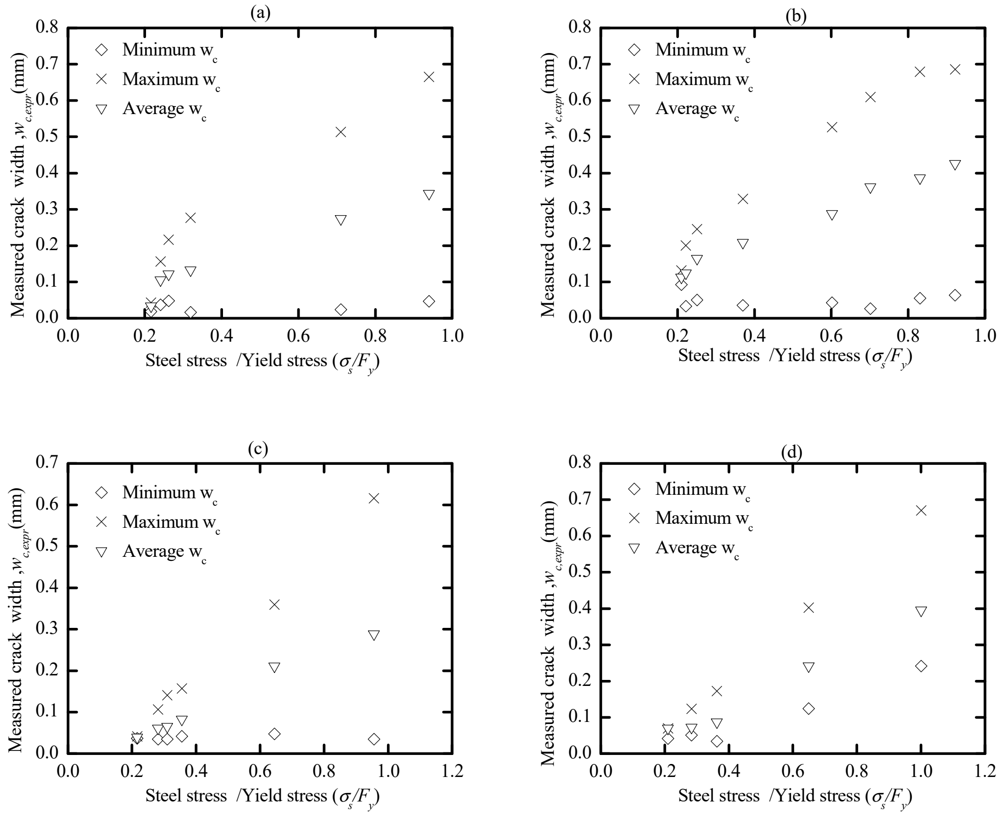

Figure S5 Crack width vs. steel stress/yield stress (a) M8-FA-10 (A); (b) M8-FA-10 (B); (c) M8-FA-10 (C), (d) M8-FA-10 (D).

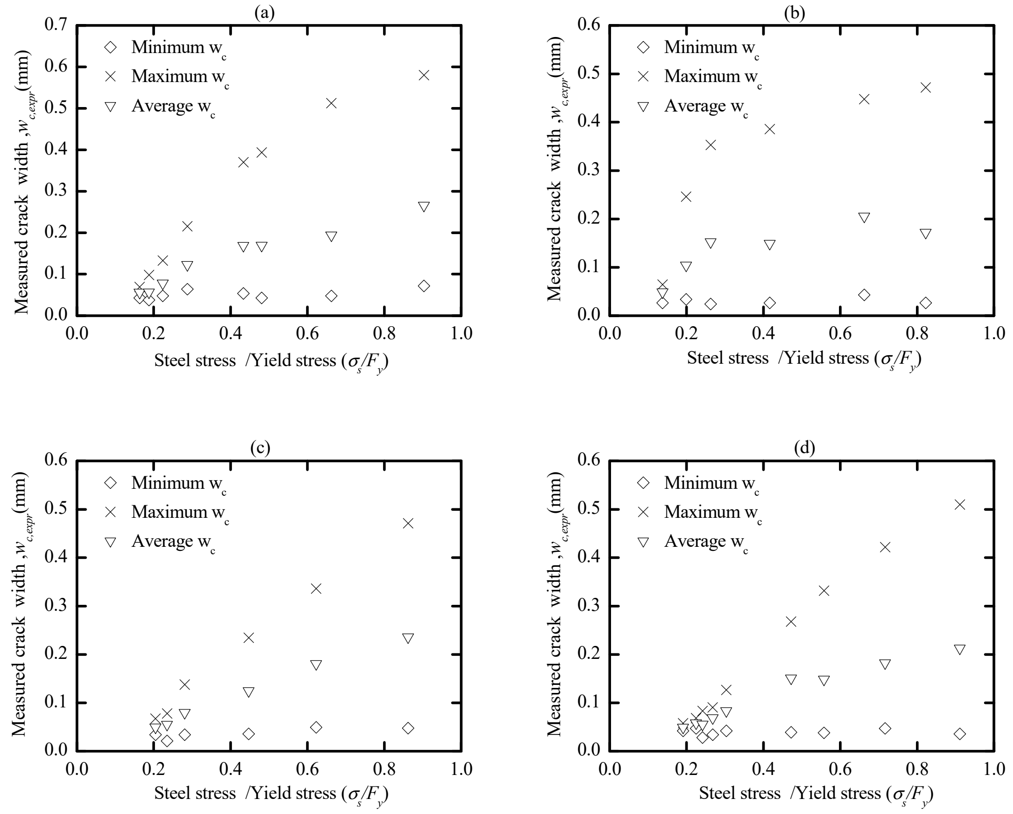

Figure S6 Crack width vs. steel stress/yield stress (a) M8-FA-12 (A); (b) M8-FA-12 (B); (c) M8-FA-12 (C), (d) M8-FA-12 (D).

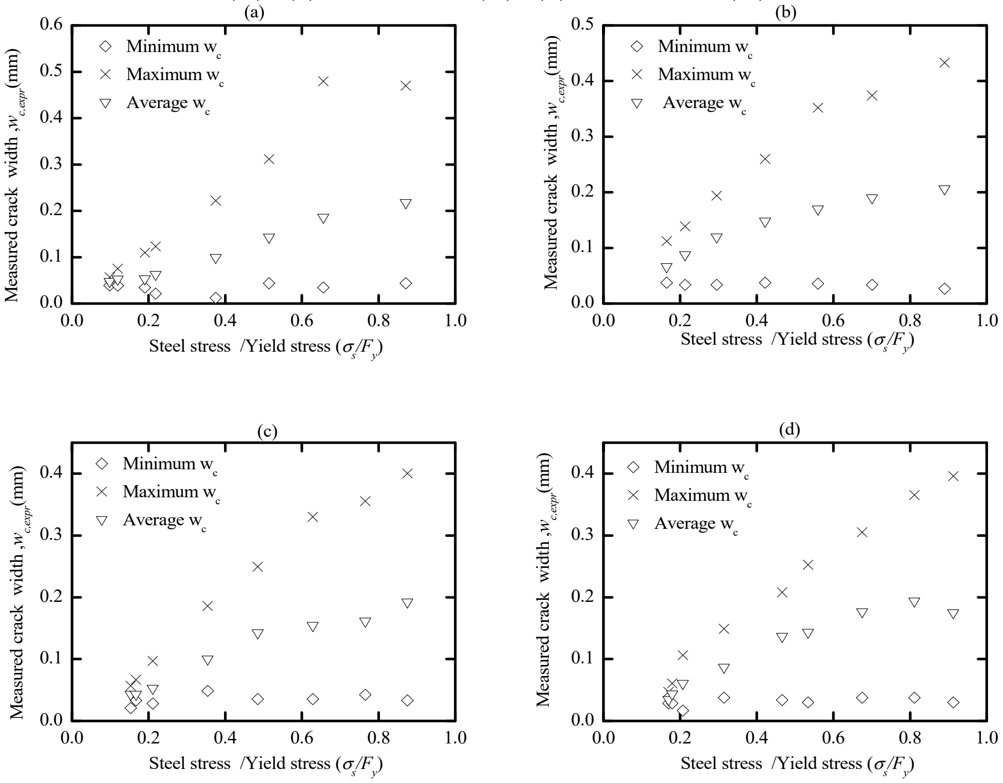

Figure S7 Crack width vs. steel stress/yield stress (a) M8-FA-16 (A); (b) M8-FA-16 (B); (c) M8-FA-16 (C), (d) M8-FA-16 (D).

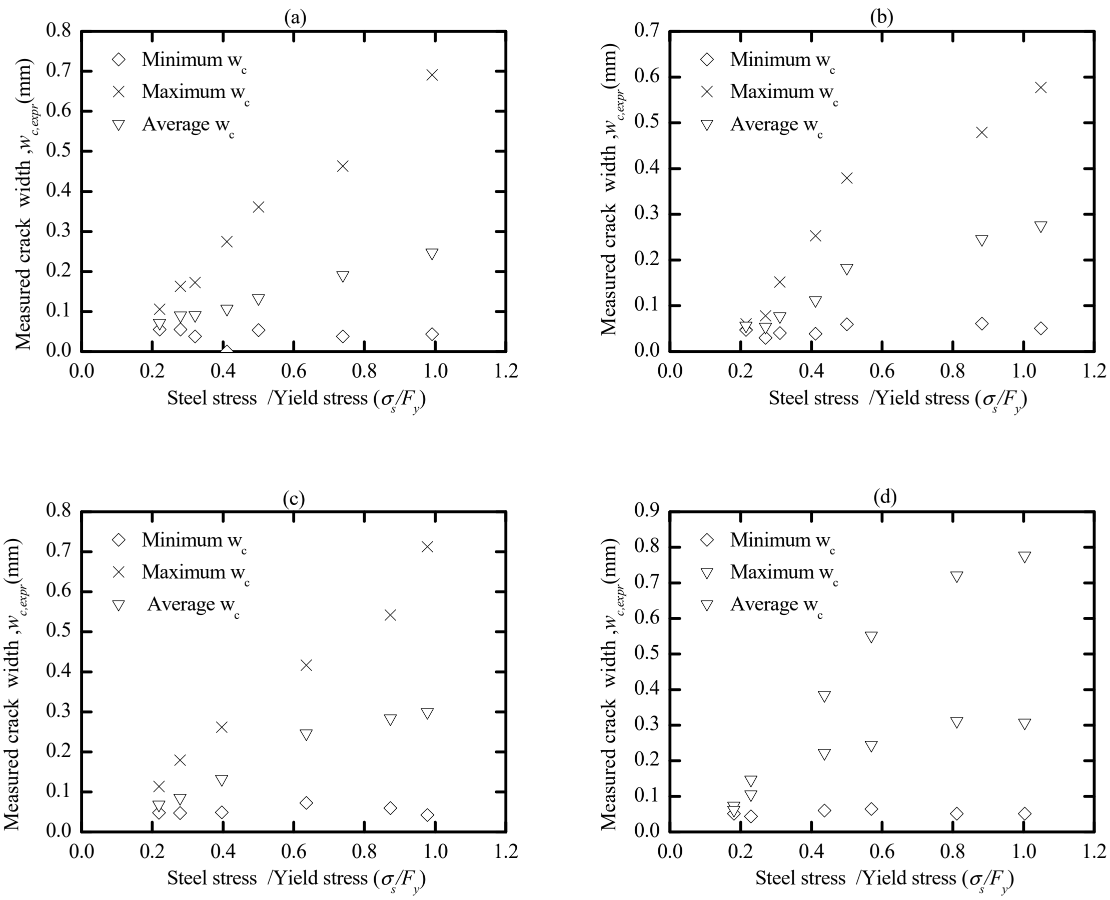

Figure S8 Crack width vs. steel stress/yield stress (a) M9-FA-10 (A); (b) M9-FA-10 (B); (c) M9-FA-10 (C), (d) M9-FA-10 (D).

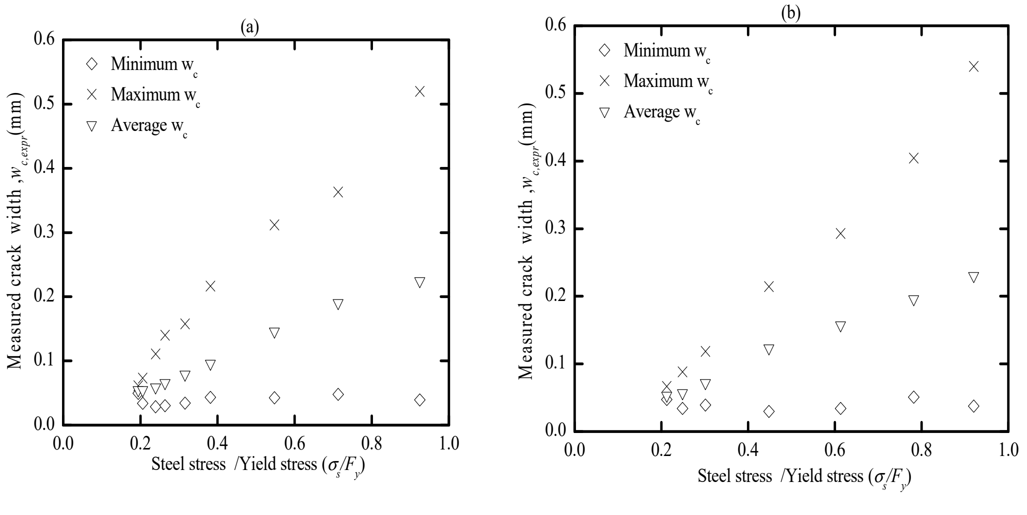

Figure S9 Crack width vs. steel stress/yield stress (a) M9-FA-12(A); (b) M9-FA-12 (B).

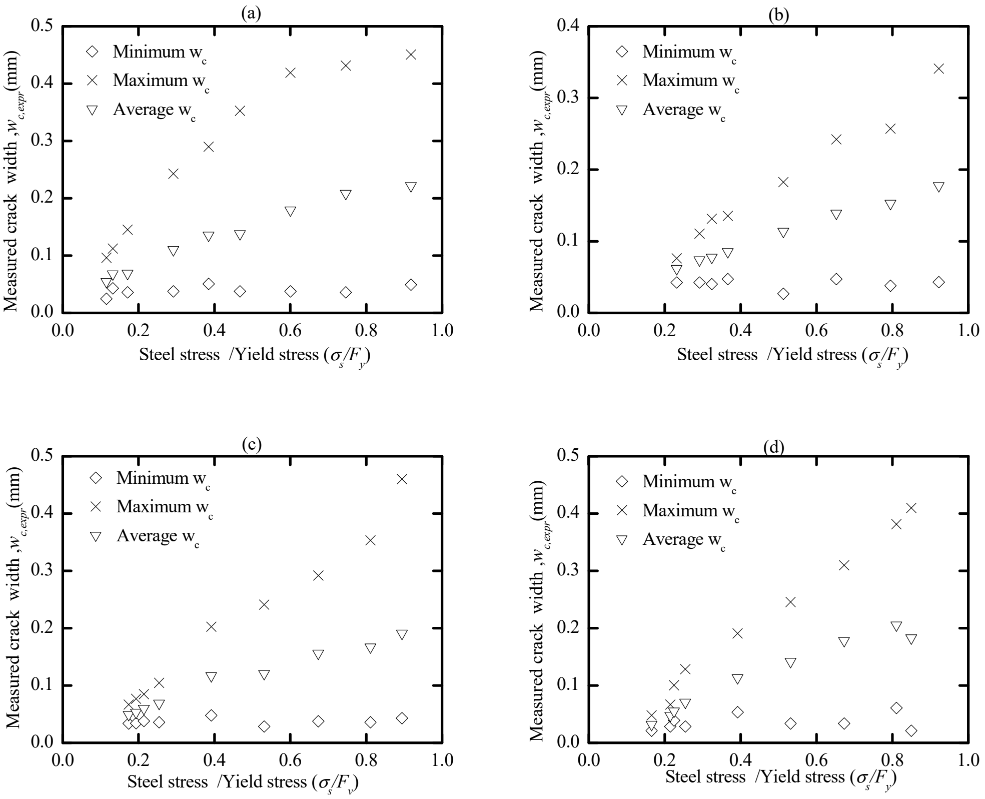

Figure S10 Crack width vs. steel stress/yield stress (a) M9-FA-16 (A); (b) M9-FA-16 (B); (c) M9-FA-16 (C), (d) M9-FA-16 (D).

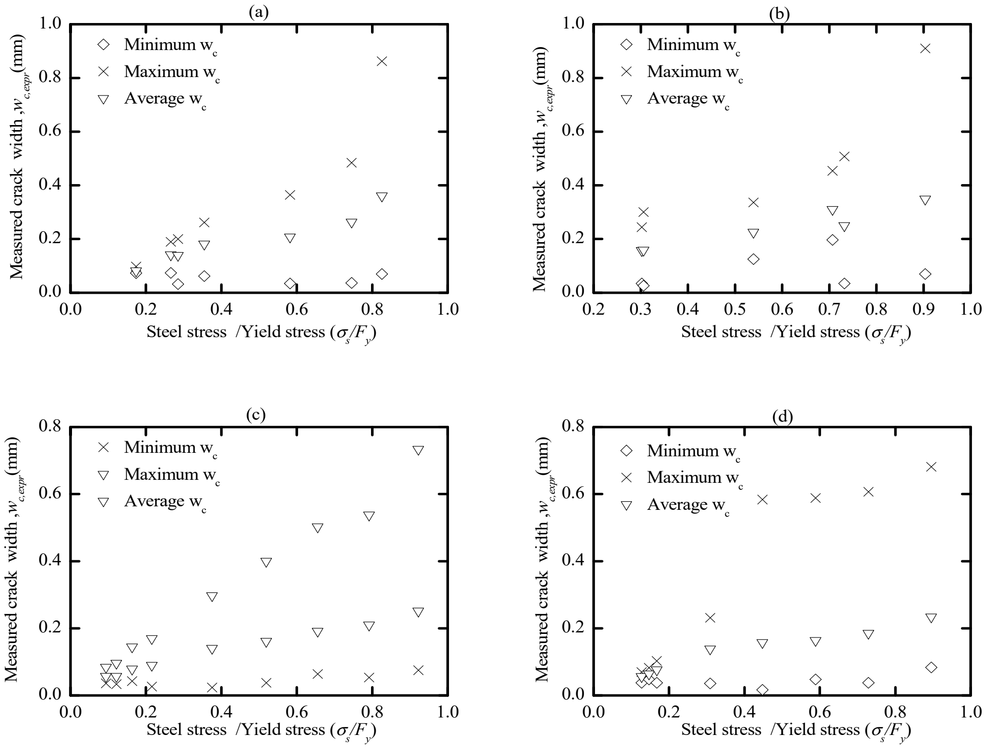

Figure S11 Crack width vs. steel stress/yield stress (a) M1-OPC-12 (A); (b) M1-OPC-12 (B); (c) M2-OPC-16 (A), (d) M2-OPC-16 (B) .

Supplementary S3 Cracking results

Table S1 Crack spacing evolution for all specimens up to the stabilized cracking stage

Specimen ID	Stage	F (kN)	ε_c (10^{-6})	$S_{min,expr}$ (mm)	$S_{av,expr}$ (mm)	$S_{max,expr}$ (mm)
M5-FA-10-(A)	1st	9.6	279	94	163	236
	2nd	12.7	437	94	130	164
	3rd	18.0	824	56	108	156
M5-FA-10-(B)	1st	8.7	189	316	325	334
	2nd	10.1	255	121	217	316
	3rd	11.4	343	85	163	316
	4th	12.5	426	24	108	185
	5th	14.7	605	24	93	131
M5-FA-10- (C)	1st	11.3	308	138	216	342
	2nd	12.4	395	76	162	342
	3rd	14.0	509	61	108	210
	4th	16.5	679	61	93	132
	5th	26.3	1312	61	93	132
	6th	33.2	1731	59	81	132
M5-FA-10-(D)	1st	10.7	239	280	325	370
	2nd	11.6	324	181	217	280
	3rd	14.2	561	89	108	189
	4th	16.1	712	89	93	99
M5-FA-12-(A)	1st	11.7	205	309	325	341
	2nd	14.3	309	76	130	193
	3rd	19.4	531	76	108	148
	4th	26.1	859	48	81	122
M5-FA-12-(B)	1st	13.37	261	77	163	241
	2nd	17.10	477	77	109	136
	3rd	25.03	882	77	109	136
	4th	35.00	1366	77	109	136
	5th	45.00	1830	77	109	136
	6th	52.02	2242	63	174	107
M5-FA-12-(C)	1st	15.6	352	60	109	211
	2nd	23.1	719	58	78	103
M5-FA-12-(D)	1st	12.4	282	119	163	233
	2nd	14.3	355	92	130	150
	3rd	16.0	429	64	108	150
	4th	20.2	624	45	93	150
	5th	30.7	1155	45	93	150
	6th	36.9	1428	45	81	119
M5-FA-16-(A)	1st	15.7	241	234	325	416
	2nd	25.8	494	102	163	234
	3rd	30.1	595	54	93	171
	4th	41.7	1118	54	81	114
M5-FA-16-(B)	1st	19.8	329	187	217	274
	2nd	22.3	396	82	130	187
	3rd	27.0	525	47	93	130
	4th	42.4	909	47	81	107
M5-FA-16-(C)	1st	16.3	264	105	163	265
	2nd	18.2	308	86	130	179
	3rd	20.3	356	75	108	179
	4th	35.3	716	45	93	179
	5th	47.4	1017	31	81	148
	6th	62.3	1350	31	81	148
	7th	77.2	1732	31	72	105
M5-FA-16-(D)	1st	19.2	245	306	325	344
	2nd	21.5	300	147	217	344
	3rd	22.9	339	80	130	174
	4th	28.1	469	73	108	159
	5th	38.1	715	66	93	147
	6th	53.1	1094	66	93	147
	7th	68.4	1474	54	81	114

Specimen ID	Stage	F (kN)	ε_c (10^{-6})	$S_{min,expr}$ (mm)	$S_{av,expr}$ (mm)	$S_{max,expr}$ (mm)
M8-FA-10-(A)	1st	9.1	70	243	325	407
	2nd	10.1	104	112	217	407
	3rd	11.0	364	112	163	233
	4th	13.4	979	71	109	131
M8-FA-10-(B)	1st	8.8	233	317	325	333
	2nd	9.3	365	145	217	317
	3rd	10.5	516	129	163	188
	4th	15.5	923	79	108	145
M8-FA-10-(C)	1st	9.1	199	323	325	327
	2nd	11.8	349	116	162	206
	3rd	13.0	436	56	125	184
	4th	14.9	609	52	93	135
M8-FA-10-(D)	1st	8.8	198	246	325	404
	2nd	11.9	419	76	109	140
	3rd	15.2	689	60	93	140
M8-FA-12-(A)	1st	9.9	232	134	325	516
	2nd	11.3	295	129	217	387
	3rd	13.5	420	129	163	248
	4th	17.4	634	94	130	154
	5th	26.2	1140	67	108	139
	6th	29.1	1298	53	93	134
M8-FA-12-(B)	1st	8.3	88	200	325	450
	2nd	12.0	292	135	163	200
	3rd	15.9	651	74	130	158
	4th	25.1	1169	57	81	126
M8-FA-12-(C)	1st	12.3	261	186	217	234
	2nd	14.2	329	84	130	234
	3rd	17.0	443	56	93	127
M8-FA-12-(D)	1st	11.5	250	314	325	336
	2nd	13.6	330	103	217	336
	3rd	14.6	381	97	163	336
	4th	16.2	451	97	130	209
	5th	18.3	553	86	108	127
	6th	28.5	1038	86	108	127
	7th	33.7	1322	37	81	123
M8-FA-16-(A)	1st	10.6	123	231	325	419
	2nd	12.8	168	179	217	240
	3rd	20.5	328	43	130	240
	4th	23.5	453	43	93	130
M8-FA-16-(B)	1st	17.7	298	115	162	199
	2nd	22.9	424	98	130	199
	3rd	31.7	665	89	108	137
	4th	45.3	988	29	93	137
M8-FA-16-(C)	1st	16.5	171	81	163	305
	2nd	17.9	189	81	130	208
	3rd	22.7	263	79	93	111
M8-FA-16-(D)	1st	18.2	235	285	325	365
	2nd	19.3	267	97	130	160
	3rd	22.3	328	61	108	160
	4th	33.8	575	61	93	136
	5th	50.1	942	61	93	136
	6th	57.3	1107	51	81	136
M9-FA-10-(A)	1st	9.3	345	106	226	319
	2nd	11.7	540	106	169	252
	3rd	13.5	697	72	130	180
	4th	17.2	1024	69	108	180
	5th	21.0	1331	69	93	139
M9-FA-10-(B)	1st	9.0	292	155	325	495
	2nd	11.3	425	151	217	344
	3rd	13.0	528	49	108	274
	4th	17.3	833	49	81	156
M9-FA-10-(C)	1st	9.2	353	94	163	247
	2nd	11.7	569	36	108	147
	3rd	16.6	1036	36	93	147

Specimen ID	Stage	F (kN)	ε_c (10^{-6})	$S_{min,expr}$ (mm)	$S_{av,expr}$ (mm)	$S_{max,expr}$ (mm)
M9-FA-10-(D)	1st	7.6	166	170	217	264
	2nd	9.6	287	98	163	216
	3rd	18.3	799	98	130	170
	4th	23.9	1162	67	93	111
M9-FA-12-(A)	1st	11.8	270	150	325	500
	2nd	12.4	309	150	217	343
	3rd	14.5	418	66	130	243
	4th	15.9	486	66	108	150
	5th	19.1	619	64	93	150
	6th	23.1	807	55	81	102
M9-FA-12-(B)	1st	12.8	343	80	162	242
	2nd	15.0	434	80	130	197
	3rd	18.2	572	53	93	130
M9-FA-16-(A)	1st	12.4	164	247	325	403
	2nd	14.2	169	95	217	403
	3rd	18.4	279	86	130	187
	4th	31.3	543	86	108	152
	5th	41.3	770	39	93	152
	6th	50.2	962	39	81	95
M9-FA-16-(B)	1st	24.9	505	147	325	503
	2nd	31.3	672	71	108	147
	3rd	34.8	756	46	93	147
	4th	39.3	868	43	81	147
	5th	55.0	1269	43	72	108
M9-FA-16-(C)	1st	18.7	315	119	217	326
	2nd	20.8	379	83	163	243
	3rd	23.0	426	83	130	205
	4th	27.3	519	58	108	147
	5th	42.1	843	58	108	147
	6th	57.0	1185	51	81	126
M9-FA-16-(C)	1st	17.8	203	80	217	462
	2nd	23.0	326	80	163	344
	3rd	24.1	316	80	108	130
	4th	27.3	445	60	93	118
M1-OPC-12 (A)	1st	10.5	104	152	325	498
	2nd	16.1	324	94	217	404
	3rd	17.2	522	94	163	270
	4th	21.4	870	94	130	152
M1-OPC-12 (B)	1st	18.3	292	274	325	376
	2nd	18.5	510	141	217	274
	3rd	32.6	905	141	217	274
	4th	42.7	1225	141	217	274
	5th	44.2	1469	106	163	235
M2-OPC-16 (A)	1st	10.1	174	178	217	272
	2nd	13.0	200	87	163	200
	3rd	17.5	315	78	130	185
	4th	23.2	462	69	108	178
M2-OPC-16 (B)	1st	13.7	155	138	217	265
	2nd	15.8	272	80	163	265
	3rd	18.0	372	73	108	147
	4th	33.2	716	73	108	147
	5th	48.1	1118	73	108	147
	6th	63.2	1515	54	93	138

Table S2 Comparison between the experimental and predicted average and maximum crack spacing of all specimens.

Reference	Specimen ID.	Average crack spacing (mm)				Maximum crack spacing (mm)			
		$S_{av,exp}$	S_{pred}^{a}	S_{pred}^{b}	S_{pred}^{c}	$S_{max,exp}$	$S_{max,pred}^{a}$	$S_{max,pred}^{b}$	$S_{max,pred}^{d}$
Present study	M5-FA-10-(A)	108	65	131	97	156	111	196	136
	M5-FA-10-(B)	93	65	131	97	131	111	196	136
	M5-FA-10-(C)	81	65	131	97	132	111	196	136
	M5-FA-10-(D)	93	65	131	97	99	111	196	136
	M5-FA-12-(A)	81	63	108	90	122	107	162	128
	M5-FA-12-(B)	93	63	108	90	135	107	162	128
	M5-FA-12-(C)	94	63	108	90	135	107	162	128
	M5-FA-12-(D)	81	63	108	90	119	107	162	128
	M5-FA-16-(A)	81	59	80	79	114	100	120	116
	M5-FA-16-(B)	81	59	80	79	107	100	120	116
	M5-FA-16-(C)	72	59	80	79	105	100	120	116
	M5-FA-16-(D)	81	59	80	79	101	100	120	116
	M8-FA-10-(A)	109	65	131	97	131	111	196	136
	M8-FA-10-(B)	108	65	131	97	145	111	196	136
	M8-FA-10-(C)	93	65	131	97	135	111	196	136
	M8-FA-10-(D)	93	65	131	97	140	111	196	136
	M8-FA-12-(A)	93	63	108	90	134	107	162	128
	M8-FA-12-(B)	81	63	108	90	126	107	162	128
	M8-FA-12-(C)	93	63	108	90	127	107	162	128
	M8-FA-12-(D)	81	63	108	90	123	107	162	128
	M8-FA-16-(A)	93	59	80	79	130	100	120	116
	M8-FA-16-(B)	93	59	80	79	137	100	120	116
	M8-FA-16-(C)	93	59	80	79	111	100	120	116
	M8-FA-16-(D)	81	59	80	79	136	100	120	116
	M9-FA-10-(A)	93	65	131	97	139	111	196	136
	M9-FA-10-(B)	81	65	131	97	156	111	196	136
	M9-FA-10-(C)	93	65	131	97	147	111	196	136
	M9-FA-10-(D)	93	65	131	97	111	111	196	136
	M9-FA-12-(A)	81	63	108	90	102	107	162	128
	M9-FA-12-(C)	93	63	108	90	130	107	162	128
	M9-FA-16-(A)	81	59	80	79	95	100	120	116
	M9-FA-16-(B)	72	59	80	79	108	100	120	116
	M9-FA-16-(C)	81	59	80	79	126	100	120	116
	M9-FA-16-(D)	93	59	80	79	118	100	120	116
	M1-OPC-12-(A)	130	63	108	-	152	107	162	-
	M1-OPC-12-(B)	163	63	108	-	235	107	162	-
	M1-OPC-16-(A)	108	59	80	-	178	100	120	-
	M1-OPC-16-(B)	93	59	80	-	138	100	120	-
Albitar	FA 1-12-75	108	63	108	90	140	107	162	128
	FA 2-12-75	93	63	108	90	135	107	162	128
	FA 3-16-75	81	59	80	79	140	100	120	116
	FA 4-16-75	93	59	80	79	130	100	120	116

Notation: [a] predicted value according to Eurocode 2, [b] predicted value according to CEB-FIP 1990, [c] predicted value according to proposed model (Table 7), [d] predicted value according to proposed model.

Table S3 Comparison between the experimental and predicted maximum crack width of all specimens.

Reference	Specimen ID	Maximum crack width				
		$W_{Av,exp}$	$W^{a}_{Av,predict}$	$W^{b}_{Av,predict}$	$W^{c}_{Max,predict}$	$W^{d}_{Max,predict}$
Present study	M5-FA-10-A	0.52	0.49	0.38	0.43	0.52
	M5-FA-10-B	0.51	0.41	0.42	0.46	0.56
	M5-FA-10-C	0.47	0.42	0.39	0.43	0.53
	M5-FA-10-D	0.50	0.31	0.40	0.45	0.54
	M5-FA-12-A	0.44	0.28	0.34	0.42	0.52
	M5-FA-12-B	0.50	0.31	0.33	0.41	0.50
	M5-FA-12-C	0.44	0.31	0.33	0.41	0.50
	M5-FA-12-D	0.50	0.27	0.33	0.41	0.50
	M5-FA-16-A	0.44	0.25	0.27	0.42	0.52
	M5-FA-16-B	0.43	0.24	0.24	0.38	0.46
	M5-FA-16-C	0.42	0.23	0.25	0.40	0.49
	M5-FA-16-D	0.38	0.22	0.25	0.39	0.48
	M8-FA-10-A	0.67	0.42	0.40	0.44	0.54
	M8-FA-10-B	0.69	0.46	0.38	0.42	0.52
	M8-FA-10-C	0.62	0.43	0.40	0.44	0.53
	M8-FA-10-D	0.67	0.45	0.42	0.46	0.56
	M8-FA-12-A	0.58	0.31	0.30	0.38	0.46
	M8-FA-12-B	0.47	0.29	0.33	0.41	0.50
	M8-FA-12-C	0.47	0.29	0.31	0.39	0.48
	M8-FA-12-D	0.51	0.29	0.34	0.42	0.51
	M8-FA-16-A	0.48	0.29	0.25	0.39	0.48
	M8-FA-16-B	0.43	0.31	0.26	0.40	0.48
	M8-FA-16-C	0.40	0.25	0.26	0.40	0.49
	M8-FA-16-D	0.40	0.31	0.27	0.41	0.50
	M9-FA-10-A	0.69	0.45	0.43	0.45	0.55
	M9-FA-10-B	0.58	0.51	0.44	0.48	0.58
	M9-FA-10-C	0.71	0.48	0.42	0.45	0.54
	M9-FA-10-D	0.78	0.36	0.44	0.46	0.56
	M9-FA-12-C	0.52	0.24	0.35	0.42	0.51
	M9-FA-12-D	0.54	0.31	0.35	0.42	0.51
	M9-FA-16-A	0.45	0.22	0.26	0.40	0.49
	M9-FA-16-B	0.34	0.25	0.27	0.42	0.51
	M9-FA-16-C	0.46	0.29	0.27	0.41	0.50
	M9-FA-16-D	0.41	0.27	0.26	0.39	0.48

Notation: [a] predicted value according to Eurocode 2, [b] predicted value according to CEB-FIP 1990, [c] predicted value according to ACI 224, [d] predicted value according to proposal model.

Table S4 Crack widths at different loading stages

Spec. ID.	Sta. NO.	P (kN)	(σ/Fy)	1st crack			2nd crack			3rd crack			4th crack			5th crack			6th crack			7th crack			8th crack			Min. crack width	Max. crack width	Ave. crack width
				Min.	Max.	Ave.	Min.	Max.	Ave.	Min.	Max.	Ave.	Min.	Max.	Ave.	Min.	Max.	Ave.	Min.	Max.	Ave.	Min.	Max.	Ave.	Min.	Max.	Ave.	Min.	Max.	Ave.
M5-FA-10-(A)	1st	9.4	0.22	0.06	0.16	0.12	0.05	0.08	0.07	0.03	0.09	0.07	-	-	-	-	-	-	-	-	-	-	-	-	-	-	-	0.03	0.16	0.08
	2nd	12.7	0.30	0.06	0.24	0.15	0.04	0.09	0.07	0.06	0.17	0.11	0.06	0.07	0.06	-	-	-	-	-	-	-	-	-	-	-	-	0.04	0.24	0.10
	3rd	18.0	0.43	0.07	0.36	0.16	0.10	0.16	0.13	0.11	0.22	0.17	0.06	0.22	0.15	0.04	0.10	0.07	-	-	-	-	-	-	-	-	-	0.04	0.36	0.14
	4th	30.0	0.72	0.04	0.44	0.21	0.03	0.35	0.18	0.16	0.39	0.29	0.11	0.46	0.31	0.09	0.26	0.18	-	-	-	-	-	-	-	-	-	0.03	0.46	0.23
	5th	38.9	0.93	0.06	0.45	0.24	0.04	0.44	0.24	0.12	0.48	0.35	0.06	0.52	0.35	0.07	0.38	0.22	-	-	-	-	-	-	-	-	-	0.04	0.52	0.28
M5-FA-10-(B)	1st	8.7	0.21	0.03	0.04	0.03	-	-	-	-	-	-	-	-	-	-	-	-	-	-	-	-	-	-	-	-	-	0.03	0.04	0.03
	2nd	10.1	0.24	0.03	0.05	0.04	0.04	0.04	0.04	-	-	-	-	-	-	-	-	-	-	-	-	-	-	-	-	-	-	0.03	0.05	0.04
	3rd	11.4	0.27	0.03	0.06	0.04	0.05	0.05	0.05	0.03	0.04	0.03	-	-	-	-	-	-	-	-	-	-	-	-	-	-	-	0.03	0.06	0.04
	4th	12.5	0.30	0.04	0.07	0.06	0.04	0.06	0.05	0.04	0.05	0.04	0.05	0.09	0.07	0.05	0.06	0.06	-	-	-	-	-	-	-	-	-	0.04	0.09	0.06
	5th	14.7	0.35	0.03	0.10	0.05	0.02	0.07	0.05	0.06	0.10	0.08	0.10	0.15	0.14	0.06	0.09	0.07	0.05	0.12	0.08	-	-	-	-	-	-	0.02	0.15	0.08
	6th	25.0	0.60	0.13	0.20	0.17	0.11	0.27	0.22	0.04	0.30	0.16	0.08	0.39	0.19	0.06	0.13	0.10	0.06	0.24	0.14	-	-	-	-	-	-	0.04	0.39	0.16
	7th	35.1	0.84	0.04	0.27	0.20	0.03	0.48	0.28	0.05	0.44	0.22	0.16	0.50	0.30	0.08	0.20	0.15	0.05	0.37	0.23	-	-	-	-	-	-	0.03	0.50	0.23
	8th	39.6	0.94	0.04	0.30	0.22	0.02	0.51	0.29	0.03	0.51	0.26	0.16	0.50	0.31	0.05	0.31	0.19	0.05	0.41	0.22	-	-	-	-	-	-	0.02	0.51	0.25
M5-FA-10-(C)	1st	11.3	0.27	0.03	0.05	0.04	0.04	0.04	0.04	-	-	-	-	-	-	-	-	-	-	-	-	-	-	-	-	-	-	0.03	0.05	0.04
	2nd	12.4	0.30	0.05	0.08	0.06	0.05	0.05	0.05	0.04	0.04	0.04	-	-	-	-	-	-	-	-	-	-	-	-	-	-	-	0.04	0.08	0.05
	3rd	14.0	0.33	0.09	0.13	0.11	0.06	0.07	0.06	0.06	0.07	0.06	0.03	0.04	0.03	0.05	0.10	0.07	-	-	-	-	-	-	-	-	-	0.03	0.13	0.07
	4th	16.5	0.39	0.08	0.17	0.12	0.04	0.08	0.06	0.04	0.10	0.07	0.03	0.12	0.07	0.05	0.16	0.11	0.04	0.05	0.04	-	-	-	-	-	-	0.03	0.17	0.08
	5th	26.3	0.63	0.05	0.30	0.17	0.06	0.14	0.11	0.10	0.27	0.19	0.03	0.28	0.16	0.08	0.26	0.19	0.04	0.15	0.11	-	-	-	-	-	-	0.03	0.30	0.15
	6th	33.2	0.79	0.06	0.40	0.23	0.11	0.24	0.16	0.11	0.31	0.23	0.12	0.37	0.28	0.11	0.28	0.20	0.08	0.20	0.15	0.05	0.23	0.16	-	-	-	0.05	0.40	0.20
	7th	40.8	0.97	0.07	0.47	0.24	0.15	0.47	0.24	0.16	0.40	0.29	0.16	0.45	0.31	0.13	0.36	0.26	0.08	0.25	0.19	0.11	0.35	0.24	-	-	-	0.07	0.47	0.25
M5-FA-10-(D)	1st	10.7	0.25	0.05	0.07	0.06	-	-	-	-	-	-	-	-	-	-	-	-	-	-	-	-	-	-	-	-	-	0.05	0.07	0.06
	2nd	11.6	0.28	0.04	0.10	0.07	0.05	0.06	0.06	-	-	-	-	-	-	-	-	-	-	-	-	-	-	-	-	-	-	0.04	0.10	0.06
	3rd	14.2	0.34	0.03	0.18	0.10	0.05	0.13	0.09	0.03	0.05	0.04	0.05	0.11	0.07	0.06	0.09	0.07	-	-	-	-	-	-	-	-	-	0.03	0.18	0.08
	4th	16.1	0.38	0.07	0.19	0.13	0.04	0.17	0.11	0.02	0.10	0.07	0.08	0.14	0.10	0.11	0.14	0.12	0.03	0.10	0.06	-	-	-	-	-	-	0.02	0.19	0.10
	5th	28.3	0.68	0.06	0.30	0.21	0.05	0.29	0.18	0.05	0.28	0.18	0.19	0.25	0.21	0.16	0.28	0.23	0.05	0.29	0.19	-	-	-	-	-	-	0.05	0.30	0.20
	6th	42.0	1.00	0.06	0.45	0.31	0.04	0.40	0.25	0.04	0.45	0.29	0.04	0.35	0.21	0.06	0.41	0.24	0.06	0.50	0.29	-	-	-	-	-	-	0.04	0.50	0.26
M5-FA-12-(A)	1st	11.7	0.19	0.05	0.09	0.06	-	-	-	-	-	-	-	-	-	-	-	-	-	-	-	-	-	-	-	-	-	0.05	0.09	0.06
	2nd	14.3	0.24	0.05	0.10	0.07	0.03	0.11	0.06	0.05	0.15	0.08	0.05	0.05	0.05	-	-	-	-	-	-	-	-	-	-	-	-	0.03	0.15	0.07
	3rd	19.4	0.32	0.08	0.13	0.10	0.04	0.24	0.08	0.04	0.17	0.09	0.05	0.09	0.07	0.03	0.05	0.05	-	-	-	-	-	-	-	-	-	0.03	0.24	0.08
	4th	26.1	0.43	0.11	0.17	0.14	0.04	0.25	0.11	0.05	0.20	0.12	0.07	0.14	0.11	0.03	0.08	0.05	0.05	0.13	0.10	0.04	0.11	0.07	-	-	-	0.03	0.25	0.10
	5th	34.0	0.56	0.04	0.29	0.18	0.04	0.26	0.12	0.06	0.25	0.14	0.06	0.28	0.16	0.05	0.09	0.07	0.07	0.28	0.17	0.02	0.30	0.17	-	-	-	0.02	0.30	0.15
	6th	56.0	0.93	0.06	0.39	0.22	0.06	0.27	0.14	0.09	0.40	0.21	0.06	0.38	0.24	0.07	0.18	0.12	0.12	0.44	0.32	0.07	0.43	0.23	-	-	-	0.06	0.44	0.21
M5-FA-12-(B)	1st	13.4	0.22	0.06	0.11	0.08	0.05	0.12	0.08	0.03	0.07	0.05	-	-	-	-	-	-	-	-	-	-	-	-	-	-	-	0.03	0.12	0.07
	2nd	17.1	0.28	0.10	0.16	0.13	0.06	0.14	0.10	0.08	0.12	0.10	0.06	0.09	0.07	0.05	0.10	0.07	-	-	-	-	-	-	-	-	-	0.05	0.16	0.09

Spec. ID.	Sta. NO.	P (kN)	(σ/Fy)	1st crack			2nd crack			3rd crack			4th crack			5th crack			6th crack			7th crack			8th crack			Min. crack width	Max. crack width	Ave. crack width
				Min.	Max.	Ave.	Min.	Max.	Ave.	Min.	Max.	Ave.	Min.	Max.	Ave.	Min.	Max.	Ave.	Min.	Max.	Ave.	Min.	Max.	Ave.	Min.	Max.	Ave.	Min.	Max.	Ave.
	3rd	25.0	0.41	0.05	0.21	0.12	0.15	0.20	0.17	0.04	0.21	0.13	0.13	0.25	0.19	0.08	0.19	0.14	-	-	-	-	-	-	-	-	-	0.04	0.25	0.15
	4th	35.0	0.58	0.05	0.25	0.17	0.05	0.30	0.21	0.05	0.35	0.20	0.06	0.35	0.23	0.05	0.32	0.20	-	-	-	-	-	-	-	-	-	0.05	0.35	0.20
	5th	45.0	0.75	0.06	0.31	0.19	0.08	0.38	0.28	0.05	0.36	0.22	0.06	0.46	0.32	0.12	0.37	0.28	-	-	-	-	-	-	-	-	-	0.05	0.46	0.26
	6th	52.0	0.86	0.09	0.40	0.22	0.11	0.44	0.28	0.04	0.49	0.27	0.03	0.49	0.32	0.04	0.50	0.31	0.05	0.17	0.13	-	-	-	-	-	-	0.03	0.50	0.26
M5-FA-12-(C)	1st	15.6	0.26	0.07	0.11	0.09	0.06	0.07	0.06	0.07	0.08	0.07	0.08	0.08	0.08	0.04	0.04	0.04	-	-	-	-	-	-	-	-	-	0.04	0.11	0.07
	2nd	23.1	0.38	0.07	0.19	0.15	0.06	0.12	0.10	0.08	0.13	0.12	0.07	0.14	0.10	0.08	0.13	0.11	0.05	0.07	0.06	0.06	0.06	0.06	-	-	-	0.05	0.19	0.10
	3rd	33.7	0.56	0.07	0.28	0.20	0.08	0.22	0.15	0.11	0.23	0.18	0.12	0.28	0.19	0.05	0.23	0.15	0.15	0.22	0.18	0.06	0.13	0.10	-	-	-	0.05	0.28	0.16
	4th	43.1	0.71	0.10	0.35	0.24	0.04	0.28	0.18	0.05	0.31	0.22	0.05	0.33	0.19	0.07	0.34	0.20	0.19	0.29	0.23	0.06	0.26	0.13	-	-	-	0.04	0.35	0.20
	5th	54.3	0.90	0.07	0.44	0.27	0.07	0.36	0.23	0.09	0.39	0.25	0.04	0.36	0.21	0.09	0.35	0.25	0.21	0.38	0.30	0.04	0.36	0.20	-	-	-	0.04	0.44	0.24
M5-FA-12-(D)	1st	12.4	0.21	0.04	0.08	0.06	0.04	0.05	0.04	0.06	0.09	0.07	-	-	-	-	-	-	-	-	-	-	-	-	-	-	-	0.04	0.09	0.06
	2nd	14.3	0.24	0.05	0.09	0.07	0.06	0.06	0.06	0.04	0.11	0.07	0.04	0.05	0.05	-	-	-	-	-	-	-	-	-	-	-	-	0.04	0.11	0.06
	3rd	16.0	0.26	0.04	0.11	0.06	0.06	0.07	0.07	0.04	0.13	0.09	0.04	0.06	0.05	0.05	0.06	0.05	-	-	-	-	-	-	-	-	-	0.04	0.13	0.06
	4th	20.2	0.33	0.06	0.20	0.13	0.03	0.09	0.06	0.05	0.16	0.11	0.05	0.09	0.07	0.05	0.10	0.07	0.04	0.06	0.05	-	-	-	-	-	-	0.03	0.20	0.08
	5th	30.7	0.51	0.03	0.31	0.19	0.04	0.19	0.10	0.05	0.22	0.14	0.03	0.19	0.11	0.06	0.16	0.10	0.06	0.07	0.07	-	-	-	-	-	-	0.03	0.31	0.12
	6th	36.9	0.61	0.06	0.43	0.23	0.03	0.25	0.14	0.05	0.25	0.15	0.05	0.22	0.14	0.06	0.22	0.13	0.04	0.08	0.05	0.07	0.11	0.10	-	-	-	0.03	0.43	0.14
	7th	47.1	0.78	0.06	0.48	0.32	0.04	0.33	0.18	0.06	0.33	0.18	0.03	0.28	0.17	0.03	0.30	0.10	0.04	0.11	0.07	0.11	0.19	0.16	-	-	-	0.03	0.48	0.17
	8th	55.0	0.91	0.07	0.50	0.32	0.05	0.34	0.19	0.09	0.39	0.20	0.03	0.32	0.19	0.06	0.36	0.17	0.06	0.15	0.10	0.11	0.27	0.19	-	-	-	0.03	0.50	0.20
M5-FA-16-(A)	1st	15.7	0.15	0.04	0.07	0.05	-	-	-	-	-	-	-	-	-	-	-	-	-	-	-	-	-	-	-	-	-	0.04	0.07	0.05
	2nd	25.8	0.24	0.04	0.10	0.06	0.03	0.09	0.06	0.05	0.07	0.06	-	-	-	-	-	-	-	-	-	-	-	-	-	-	-	0.03	0.10	0.06
	3rd	30.1	0.28	0.05	0.12	0.07	0.04	0.14	0.09	0.05	0.08	0.07	0.05	0.10	0.08	0.03	0.07	0.05	0.05	0.08	0.06	-	-	-	-	-	-	0.03	0.14	0.07
	4th	41.7	0.39	0.04	0.14	0.07	0.05	0.21	0.14	0.05	0.12	0.08	0.06	0.14	0.09	0.06	0.15	0.10	0.04	0.13	0.08	0.07	0.15	0.10	-	-	-	0.04	0.21	0.10
	5th	55.0	0.51	0.05	0.18	0.11	0.07	0.27	0.18	0.09	0.18	0.13	0.06	0.17	0.10	0.09	0.18	0.12	0.06	0.15	0.11	0.07	0.22	0.16	-	-	-	0.05	0.27	0.13
	6th	70.2	0.65	0.03	0.22	0.13	0.06	0.33	0.22	0.08	0.21	0.16	0.08	0.22	0.15	0.07	0.22	0.14	0.04	0.18	0.12	0.09	0.26	0.17	-	-	-	0.03	0.33	0.16
	7th	85.7	0.80	0.05	0.29	0.17	0.16	0.38	0.29	0.15	0.25	0.20	0.12	0.26	0.17	0.10	0.29	0.20	0.07	0.22	0.15	0.17	0.35	0.26	-	-	-	0.05	0.38	0.21
	8th	####	0.93	0.04	0.32	0.19	0.06	0.44	0.28	0.12	0.38	0.22	0.10	0.33	0.21	0.09	0.31	0.21	0.13	0.24	0.19	0.10	0.38	0.24	-	-	-	0.04	0.44	0.22
M5-FA-16-(B)	1st	19.8	0.18	0.04	0.07	0.05	0.02	0.05	0.04	-	-	-	-	-	-	-	-	-	-	-	-	-	-	-	-	-	-	0.02	0.07	0.04
	2nd	22.3	0.21	0.04	0.09	0.06	0.06	0.07	0.06	0.04	0.08	0.05	0.03	0.09	0.06	-	-	-	-	-	-	-	-	-	-	-	-	0.03	0.09	0.06
	3rd	27.0	0.25	0.04	0.10	0.08	0.03	0.08	0.06	0.03	0.09	0.06	0.06	0.15	0.10	0.04	0.09	0.07	0.02	0.09	0.05	-	-	-	-	-	-	0.02	0.15	0.07
	4th	42.4	0.39	0.05	0.17	0.11	0.04	0.14	0.09	0.04	0.17	0.10	0.12	0.20	0.17	0.05	0.18	0.11	0.05	0.11	0.08	0.06	0.09	0.07	-	-	-	0.04	0.20	0.11
	5th	60.1	0.56	0.05	0.23	0.13	0.04	0.20	0.12	0.03	0.26	0.14	0.09	0.30	0.19	0.09	0.26	0.17	0.04	0.10	0.07	0.05	0.15	0.09	-	-	-	0.03	0.30	0.13
	6th	75.2	0.70	0.06	0.26	0.14	0.06	0.23	0.15	0.02	0.29	0.14	0.07	0.39	0.23	0.11	0.29	0.20	0.04	0.17	0.10	0.04	0.23	0.14	-	-	-	0.02	0.39	0.16
	7th	94.0	0.88	0.06	0.30	0.18	0.05	0.28	0.15	0.03	0.35	0.20	0.09	0.43	0.25	0.06	0.30	0.17	0.05	0.20	0.12	0.04	0.26	0.13	-	-	-	0.03	0.43	0.17
M5-FA-16-(C)	1st	16.3	0.15	0.03	0.04	0.04	0.04	0.06	0.05	0.03	0.03	0.03	-	-	-	-	-	-	-	-	-	-	-	-	-	-	-	0.03	0.06	0.04
	2nd	18.2	0.17	0.04	0.09	0.05	0.05	0.09	0.07	0.02	0.08	0.05	0.05	0.07	0.06	-	-	-	-	-	-	-	-	-	-	-	-	0.02	0.09	0.06
	3rd	20.3	0.19	0.05	0.10	0.06	0.06	0.14	0.10	0.06	0.09	0.07	0.05	0.08	0.06	0.04	0.05	0.04	-	-	-	-	-	-	-	-	-	0.04	0.14	0.07

Spec. ID.	Sta. NO.	P (kN)	(σ/Fy)	1st crack			2nd crack			3rd crack			4th crack			5th crack			6th crack			7th crack			8th crack			Min. crack width	Max. crack width	Ave. crack width
				Min.	Max.	Ave.	Min.	Max.	Ave.	Min.	Max.	Ave.	Min.	Max.	Ave.	Min.	Max.	Ave.	Min.	Max.	Ave.	Min.	Max.	Ave.	Min.	Max.	Ave.	Min.	Max.	Ave.
	4th	35.3	0.33	0.04	0.19	0.12	0.07	0.22	0.14	0.03	0.18	0.11	0.05	0.14	0.08	0.07	0.13	0.10	0.03	0.06	0.04	-	-	-	-	-	-	0.03	0.22	0.10
	5th	47.4	0.44	0.05	0.26	0.15	0.06	0.29	0.16	0.08	0.25	0.14	0.05	0.18	0.11	0.11	0.19	0.15	0.03	0.07	0.05	0.05	0.05	0.05	-	-	-	0.03	0.29	0.11
	6th	62.3	0.58	0.05	0.29	0.18	0.08	0.33	0.20	0.06	0.27	0.15	0.07	0.27	0.16	0.06	0.26	0.17	0.02	0.11	0.08	0.06	0.06	0.06	-	-	-	0.02	0.33	0.14
	7th	77.2	0.72	0.04	0.33	0.19	0.09	0.41	0.22	0.07	0.33	0.19	0.04	0.29	0.14	0.08	0.29	0.16	0.05	0.20	0.12	0.06	0.08	0.07	0.09	0.10	0.10	0.04	0.41	0.15
	8th	92.6	0.86	0.05	0.34	0.18	0.04	0.38	0.21	0.06	0.42	0.21	0.04	0.34	0.18	0.06	0.30	0.17	0.04	0.21	0.13	0.06	0.09	0.07	0.11	0.13	0.12	0.04	0.42	0.16
M5-FA-16-(D)	1st	19.2	0.18	0.03	0.03	0.03	-	-	-	-	-	-	-	-	-	-	-	-	-	-	-	-	-	-	-	-	-	0.03	0.03	0.03
	2nd	21.5	0.20	0.04	0.04	0.04	0.06	0.06	0.06	-	-	-	-	-	-	-	-	-	-	-	-	-	-	-	-	-	-	0.04	0.06	0.05
	3rd	22.9	0.21	0.04	0.05	0.05	0.03	0.08	0.05	0.05	0.05	0.05	0.04	0.04	0.04	-	-	-	-	-	-	-	-	-	-	-	-	0.03	0.08	0.05
	4th	28.1	0.26	0.03	0.08	0.06	0.05	0.12	0.08	0.04	0.10	0.07	0.04	0.07	0.06	0.02	0.03	0.03	-	-	-	-	-	-	-	-	-	0.02	0.12	0.06
	5th	38.1	0.35	0.04	0.13	0.07	0.08	0.17	0.12	0.08	0.15	0.11	0.05	0.15	0.09	0.05	0.13	0.09	0.05	0.08	0.06	-	-	-	-	-	-	0.04	0.17	0.09
	6th	53.1	0.49	0.05	0.22	0.12	0.13	0.20	0.17	0.06	0.24	0.14	0.05	0.20	0.13	0.11	0.18	0.14	0.04	0.10	0.08	-	-	-	-	-	-	0.04	0.24	0.13
	7th	68.4	0.64	0.06	0.29	0.16	0.13	0.29	0.22	0.06	0.29	0.15	0.03	0.25	0.17	0.06	0.22	0.15	0.07	0.17	0.11	0.04	0.07	0.06	-	-	-	0.03	0.29	0.15
	8th	82.0	0.76	0.07	0.35	0.21	0.03	0.32	0.18	0.09	0.30	0.18	0.04	0.30	0.17	0.05	0.28	0.17	0.06	0.20	0.13	0.07	0.14	0.10	-	-	-	0.03	0.35	0.16
	9th	92.5	0.86	0.06	0.38	0.22	0.06	0.33	0.19	0.02	0.38	0.20	0.06	0.31	0.18	0.06	0.31	0.19	0.09	0.25	0.14	0.10	0.17	0.14	-	-	-	0.02	0.38	0.18
M8-FA-10-(A)	1st	3.3	0.08	0.02	0.04	0.03	-	-	-	-	-	-	-	-	-	-	-	-	-	-	-	-	-	-	-	-	-	0.02	0.04	0.03
	2nd	3.6	0.09	0.13	0.16	0.15	0.04	0.14	0.06	-	-	-	-	-	-	-	-	-	-	-	-	-	-	-	-	-	-	0.04	0.16	0.11
	3rd	11.0	0.26	0.08	0.22	0.16	0.05	0.16	0.09	0.06	0.14	0.10	-	-	-	-	-	-	-	-	-	-	-	-	-	-	-	0.05	0.22	0.12
	4th	13.4	0.32	0.04	0.28	0.18	0.06	0.19	0.12	0.02	0.24	0.17	0.04	0.09	0.08	0.04	0.18	0.11	-	-	-	-	-	-	-	-	-	0.02	0.28	0.13
	5th	29.8	0.71	0.08	0.48	0.34	0.11	0.48	0.28	0.03	0.51	0.28	0.02	0.32	0.19	0.05	0.51	0.27	-	-	-	-	-	-	-	-	-	0.02	0.51	0.27
	6th	39.4	0.94	0.05	0.52	0.37	0.11	0.56	0.30	0.08	0.63	0.42	0.05	0.49	0.29	0.06	0.67	0.34	-	-	-	-	-	-	-	-	-	0.05	0.67	0.34
M8-FA-10-(B)	1st	8.1	0.19	0.09	0.13	0.11	-	-	-	-	-	-	-	-	-	-	-	-	-	-	-	-	-	-	-	-	-	0.09	0.13	0.11
	2nd	9.3	0.22	0.09	0.20	0.15	0.03	0.14	0.09	-	-	-	-	-	-	-	-	-	-	-	-	-	-	-	-	-	-	0.03	0.20	0.12
	3rd	10.5	0.25	0.15	0.25	0.19	0.05	0.19	0.13	0.0746	0.1088	0.093	-	-	-	-	-	-	-	-	-	-	-	-	-	-	-	0.05	0.25	0.16
	4th	15.5	0.37	0.05	0.33	0.23	0.09	0.28	0.18	0.12	0.22	0.19	0.04	0.21	0.13	0.08	0.11	0.10	-	-	-	-	-	-	-	-	-	0.04	0.33	0.21
	5th	25.2	0.60	0.04	0.49	0.29	0.13	0.53	0.31	0.12	0.32	0.23	0.10	0.40	0.25	0.16	0.25	0.20	-	-	-	-	-	-	-	-	-	0.04	0.53	0.29
	6th	29.4	0.70	0.03	0.56	0.33	0.15	0.61	0.38	0.15	0.40	0.30	0.14	0.47	0.35	0.22	0.39	0.31	-	-	-	-	-	-	-	-	-	0.03	0.61	0.36
	7th	34.9	0.83	0.06	0.62	0.37	0.16	0.68	0.47	0.14	0.55	0.31	0.15	0.56	0.40	0.25	0.54	0.39	-	-	-	-	-	-	-	-	-	0.06	0.68	0.39
	8th	38.7	0.92	0.06	0.65	0.43	0.18	0.69	0.50	0.20	0.57	0.40	0.17	0.60	0.48	0.18	0.54	0.38	-	-	-	-	-	-	-	-	-	0.06	0.69	0.43
M8-FA-10-(C)	1st	9.1	0.22	0.04	0.04	0.04	-	-	-	-	-	-	-	-	-	-	-	-	-	-	-	-	-	-	-	-	-	0.04	0.04	0.04
	2nd	11.8	0.28	0.06	0.11	0.09	0.03	0.03	0.03	0.05	0.07	0.05	-	-	-	-	-	-	-	-	-	-	-	-	-	-	-	0.03	0.11	0.06
	3rd	13.0	0.31	0.06	0.14	0.10	0.04	0.06	0.05	0.05	0.10	0.08	0.03	0.05	0.04	-	-	-	-	-	-	-	-	-	-	-	-	0.03	0.14	0.07
	4th	14.9	0.36	0.06	0.16	0.11	0.06	0.08	0.07	0.09	0.12	0.10	0.09	0.11	0.10	0.05	0.10	0.07	0.04	0.05	0.04	-	-	-	-	-	-	0.04	0.16	0.08
	5th	27.1	0.65	0.05	0.34	0.19	0.09	0.26	0.18	0.27	0.36	0.31	0.08	0.23	0.15	0.21	0.32	0.26	0.09	0.22	0.16	-	-	-	-	-	-	0.05	0.36	0.21
	6th	40.1	0.96	0.03	0.45	0.25	0.09	0.40	0.21	0.37	0.62	0.47	0.11	0.28	0.19	0.34	0.50	0.42	0.05	0.30	0.20	-	-	-	-	-	-	0.03	0.62	0.29
M8-FA-10-(D)	1st	8.8	0.21	0.04	0.06	0.07	-	-	-	-	-	-	-	-	-	-	-	-	-	-	-	-	-	-	-	-	-	0.04	0.07	0.07
	2nd	11.9	0.28	0.07	0.12	0.10	0.05	0.05	0.05	0.06	0.06	0.06	0.07	0.07	0.07	-	-	-	-	-	-	-	-	-	-	-	-	0.05	0.12	0.07

Spec. ID.	Sta. NO.	P (kN)	(σ/Fy)	1st crack			2nd crack			3rd crack			4th crack			5th crack			6th crack			7th crack			8th crack			Min. crack width	Max. crack width	Ave. crack width
				Min.	Max.	Ave.	Min.	Max.	Ave.	Min.	Max.	Ave.	Min.	Max.	Ave.	Min.	Max.	Ave.	Min.	Max.	Ave.	Min.	Max.	Ave.	Min.	Max.	Ave.	Min.	Max.	Ave.
	3rd	15.2	0.36	0.14	0.17	0.15	0.11	0.15	0.13	0.05	0.13	0.09	0.03	0.10	0.06	0.03	0.03	0.03	0.05	0.08	0.06	-	-	-	-	-	-	0.03	0.17	0.09
	4th	27.2	0.65	0.31	0.40	0.37	0.30	0.35	0.33	0.17	0.23	0.21	0.13	0.23	0.18	0.12	0.18	0.16	0.17	0.24	0.21	-	-	-	-	-	-	0.12	0.40	0.24
	5th	42.0	1.00	0.50	0.67	0.59	0.40	0.58	0.49	0.25	0.32	0.29	0.24	0.31	0.28	0.33	0.43	0.37	0.30	0.42	0.36	-	-	-	-	-	-	0.24	0.67	0.40
M8-FA-12-(A)	1st	9.9	0.16	0.04	0.07	0.06	-	-	-	-	-	-	-	-	-	-	-	-	-	-	-	-	-	-	-	-	-	0.04	0.07	0.06
	2nd	11.3	0.19	0.04	0.10	0.06	0.04	0.07	0.05	-	-	-	-	-	-	-	-	-	-	-	-	-	-	-	-	-	-	0.04	0.10	0.06
	3rd	13.5	0.22	0.05	0.13	0.08	0.06	0.11	0.09	0.05	0.08	0.07	-	-	-	-	-	-	-	-	-	-	-	-	-	-	-	0.05	0.13	0.08
	4th	17.4	0.29	0.07	0.22	0.16	0.09	0.17	0.14	0.06	0.15	0.12	0.06	0.10	0.08	-	-	-	-	-	-	-	-	-	-	-	-	0.06	0.22	0.12
	5th	26.2	0.43	0.13	0.37	0.26	0.08	0.26	0.20	0.09	0.24	0.19	0.06	0.17	0.12	0.05	0.14	0.08	-	-	-	-	-	-	-	-	-	0.05	0.37	0.17
	6th	29.1	0.48	0.05	0.39	0.25	0.10	0.35	0.24	0.07	0.25	0.19	0.07	0.21	0.15	0.06	0.16	0.09	0.04	0.15	0.10	-	-	-	-	-	-	0.04	0.39	0.17
	7th	40.0	0.66	0.05	0.51	0.29	0.06	0.42	0.24	0.05	0.32	0.20	0.05	0.29	0.14	0.07	0.17	0.12	0.07	0.24	0.16	-	-	-	-	-	-	0.05	0.51	0.19
	8th	54.6	0.90	0.13	0.58	0.42	0.12	0.50	0.27	0.10	0.38	0.27	0.12	0.43	0.25	0.07	0.28	0.20	0.08	0.35	0.18	-	-	-	-	-	-	0.07	0.58	0.27
M8-FA-12-(B)	1st	3.0	0.05	0.03	0.06	0.05	-	-	-	-	-	-	-	-	-	-	-	-	-	-	-	-	-	-	-	-	-	0.03	0.06	0.05
	2nd	8.3	0.14	0.04	0.17	0.10	0.03	0.25	0.13	0.03	0.12	0.09	-	-	-	-	-	-	-	-	-	-	-	-	-	-	-	0.03	0.25	0.10
	3rd	15.9	0.26	0.04	0.23	0.14	0.02	0.35	0.18	0.04	0.26	0.17	0.03	0.20	0.12	-	-	-	-	-	-	-	-	-	-	-	-	0.02	0.35	0.15
	4th	25.1	0.42	0.06	0.30	0.20	0.03	0.39	0.22	0.09	0.37	0.25	0.03	0.22	0.14	0.06	0.10	0.07	0.04	0.08	0.06	0.06	0.14	0.10	-	-	-	0.03	0.39	0.15
	5th	40.0	0.66	0.06	0.35	0.24	0.06	0.45	0.29	0.06	0.41	0.24	0.06	0.40	0.22	0.08	0.22	0.14	0.04	0.17	0.10	0.09	0.34	0.21	-	-	-	0.04	0.45	0.21
	6th	49.7	0.82	0.05	0.40	0.24	0.06	0.47	0.24	0.05	0.43	0.20	0.04	0.43	0.17	0.04	0.25	0.12	0.03	0.20	0.07	0.04	0.37	0.17	-	-	-	0.03	0.47	0.17
M8-FA-12-(C)	1st	12.3	0.20	0.03	0.07	0.05	0.04	0.05	0.04	-	-	-	-	-	-	-	-	-	-	-	-	-	-	-	-	-	-	0.03	0.07	0.05
	2nd	14.2	0.23	0.05	0.08	0.06	0.05	0.07	0.06	0.02	0.06	0.04	0.06	0.07	0.06	-	-	-	-	-	-	-	-	-	-	-	-	0.02	0.08	0.06
	3rd	17.0	0.28	0.05	0.10	0.08	0.06	0.10	0.09	0.03	0.11	0.08	0.07	0.12	0.09	0.05	0.14	0.08	0.05	0.10	0.07	-	-	-	-	-	-	0.03	0.14	0.08
	4th	27.0	0.45	0.04	0.17	0.10	0.14	0.19	0.17	0.04	0.23	0.12	0.04	0.20	0.10	0.10	0.15	0.12	0.06	0.21	0.14	-	-	-	-	-	-	0.04	0.23	0.12
	5th	37.6	0.62	0.06	0.23	0.15	0.22	0.30	0.26	0.05	0.32	0.19	0.06	0.31	0.19	0.05	0.18	0.11	0.09	0.34	0.19	-	-	-	-	-	-	0.05	0.34	0.18
	6th	52.1	0.86	0.06	0.31	0.20	0.10	0.40	0.31	0.05	0.39	0.26	0.09	0.32	0.22	0.05	0.27	0.18	0.10	0.47	0.25	-	-	-	-	-	-	0.05	0.47	0.24
M8-FA-12-(D)	1st	11.5	0.19	0.04	0.06	0.05	-	-	-	-	-	-	-	-	-	-	-	-	-	-	-	-	-	-	-	-	-	0.04	0.06	0.05
	2nd	13.6	0.22	0.05	0.07	0.06	0.06	0.06	0.06	-	-	-	-	-	-	-	-	-	-	-	-	-	-	-	-	-	-	0.05	0.07	0.06
	3rd	14.6	0.24	0.03	0.08	0.06	0.06	0.07	0.06	0.04	0.05	0.05	-	-	-	-	-	-	-	-	-	-	-	-	-	-	-	0.03	0.08	0.06
	4th	16.2	0.27	0.03	0.09	0.07	0.05	0.09	0.07	0.05	0.09	0.07	0.06	0.09	0.07	-	-	-	-	-	-	-	-	-	-	-	-	0.03	0.09	0.07
	5th	18.3	0.30	0.06	0.10	0.08	0.09	0.11	0.10	0.04	0.13	0.09	0.06	0.10	0.08	0.05	0.09	0.06	-	-	-	-	-	-	-	-	-	0.04	0.13	0.08
	6th	28.5	0.47	0.05	0.22	0.14	0.05	0.27	0.17	0.04	0.19	0.13	0.16	0.19	0.18	0.12	0.17	0.14	-	-	-	-	-	-	-	-	-	0.04	0.27	0.15
	7th	33.7	0.56	0.05	0.29	0.17	0.09	0.33	0.22	0.05	0.25	0.17	0.17	0.22	0.19	0.15	0.20	0.17	0.04	0.05	0.04	0.05	0.10	0.07	-	-	-	0.04	0.33	0.15
	8th	43.3	0.72	0.06	0.30	0.17	0.05	0.42	0.25	0.05	0.33	0.21	0.22	0.23	0.23	0.23	0.25	0.24	0.05	0.08	0.06	0.07	0.21	0.12	-	-	-	0.05	0.42	0.18
	9th	55.0	0.91	0.05	0.34	0.17	0.04	0.51	0.28	0.07	0.37	0.25	0.05	0.31	0.24	0.06	0.35	0.27	0.10	0.14	0.11	0.04	0.33	0.18	-	-	-	0.04	0.51	0.21
M8-FA-16-(A)	1st	10.6	0.10	0.04	0.06	0.05	-	-	-	-	-	-	-	-	-	-	-	-	-	-	-	-	-	-	-	-	-	0.04	0.06	0.05
	2nd	12.8	0.12	0.04	0.07	0.05	0.05	0.08	0.06	-	-	-	-	-	-	-	-	-	-	-	-	-	-	-	-	-	-	0.04	0.08	0.05
	3rd	20.5	0.19	0.04	0.11	0.07	0.04	0.09	0.06	0.03	0.04	0.04	0.04	0.05	0.04	-	-	-	-	-	-	-	-	-	-	-	-	0.03	0.11	0.05
	4th	23.5	0.22	0.03	0.12	0.09	0.04	0.10	0.08	0.03	0.05	0.04	0.02	0.06	0.04	0.05	0.06	0.05	0.05	0.11	0.07	-	-	-	-	-	-	0.02	0.12	0.06
	5th	40.3	0.38	0.04	0.16	0.10	0.05	0.11	0.08	0.01	0.11	0.09	0.04	0.10	0.07	0.06	0.22	0.14	0.06	0.17	0.12	-	-	-	-	-	-	0.01	0.22	0.10

Spec. ID.	Sta. NO.	P (kN)	(σ/Fy)	1st crack			2nd crack			3rd crack			4th crack			5th crack			6th crack			7th crack			8th crack			Min. crack width	Max. crack width	Ave. crack width
				Min.	Max.	Ave.	Min.	Max.	Ave.	Min.	Max.	Ave.	Min.	Max.	Ave.	Min.	Max.	Ave.	Min.	Max.	Ave.	Min.	Max.	Ave.	Min.	Max.	Ave.	Min.	Max.	Ave.
	6th	55.2	0.51	0.07	0.21	0.14	0.06	0.20	0.13	0.12	0.17	0.15	0.07	0.13	0.10	0.04	0.31	0.16	0.10	0.27	0.19	-	-	-	-	-	-	0.04	0.31	0.14
	7th	70.4	0.66	0.11	0.27	0.19	0.03	0.32	0.21	0.05	0.24	0.14	0.05	0.17	0.11	0.07	0.48	0.25	0.04	0.34	0.22	-	-	-	-	-	-	0.03	0.48	0.19
	8th	93.5	0.87	0.15	0.33	0.24	0.09	0.45	0.25	0.04	0.31	0.17	0.05	0.19	0.13	0.07	0.47	0.29	0.13	0.38	0.23	-	-	-	-	-	-	0.04	0.47	0.22
M8-FA-16-(B)	1st	17.7	0.17	0.06	0.10	0.08	0.04	0.11	0.07	0.04	0.06	0.05	-	-	-	-	-	-	-	-	-	-	-	-	-	-	-	0.04	0.11	0.07
	2nd	22.9	0.21	0.10	0.14	0.12	0.09	0.12	0.10	0.08	0.08	0.08	0.03	0.06	0.05	-	-	-	-	-	-	-	-	-	-	-	-	0.03	0.14	0.09
	3rd	31.7	0.30	0.12	0.19	0.16	0.14	0.19	0.16	0.10	0.16	0.12	0.08	0.12	0.10	0.03	0.08	0.05	-	-	-	-	-	-	-	-	-	0.03	0.19	0.12
	4th	45.3	0.42	0.04	0.26	0.19	0.16	0.26	0.21	0.14	0.20	0.17	0.13	0.19	0.16	0.05	0.18	0.11	0.04	0.06	0.05	-	-	-	-	-	-	0.04	0.26	0.15
	5th	60.1	0.56	0.04	0.31	0.17	0.06	0.35	0.23	0.13	0.23	0.17	0.15	0.22	0.20	0.07	0.24	0.16	0.08	0.10	0.09	-	-	-	-	-	-	0.04	0.35	0.17
	6th	75.3	0.70	0.04	0.37	0.20	0.10	0.37	0.26	0.13	0.27	0.22	0.03	0.26	0.15	0.08	0.30	0.19	0.10	0.13	0.11	-	-	-	-	-	-	0.03	0.37	0.19
	7th	95.6	0.89	0.09	0.43	0.25	0.10	0.39	0.23	0.20	0.34	0.27	0.12	0.28	0.18	0.03	0.36	0.18	0.07	0.15	0.12	-	-	-	-	-	-	0.03	0.43	0.21
M8-FA-16-(C)	1st	16.5	0.15	0.02	0.06	0.04	0.04	0.04	0.04	0.05	0.05	0.05	-	-	-	-	-	-	-	-	-	-	-	-	-	-	-	0.02	0.06	0.04
	2nd	17.9	0.17	0.03	0.07	0.05	0.04	0.05	0.05	0.04	0.06	0.05	0.04	0.04	0.04	0.04	0.04	0.04	-	-	-	-	-	-	-	-	-	0.03	0.07	0.04
	3rd	22.7	0.21	0.05	0.10	0.07	0.05	0.08	0.06	0.05	0.07	0.06	0.04	0.08	0.05	0.03	0.05	0.04	0.03	0.04	0.04	-	-	-	-	-	-	0.03	0.10	0.05
	4th	38.0	0.35	0.09	0.14	0.12	0.09	0.15	0.13	0.06	0.12	0.10	0.05	0.19	0.10	0.05	0.12	0.08	0.05	0.09	0.07	-	-	-	-	-	-	0.05	0.19	0.10
	5th	52.0	0.48	0.14	0.19	0.17	0.14	0.21	0.18	0.09	0.17	0.14	0.05	0.25	0.14	0.04	0.21	0.11	0.08	0.15	0.12	-	-	-	-	-	-	0.04	0.25	0.14
	6th	67.5	0.63	0.13	0.30	0.20	0.06	0.25	0.16	0.04	0.18	0.11	0.05	0.33	0.17	0.04	0.26	0.16	0.07	0.22	0.13	-	-	-	-	-	-	0.04	0.33	0.15
	7th	82.2	0.77	0.05	0.36	0.19	0.04	0.29	0.15	0.05	0.19	0.12	0.06	0.34	0.19	0.05	0.33	0.17	0.08	0.24	0.15	-	-	-	-	-	-	0.04	0.36	0.16
	8th	94.0	0.88	0.06	0.37	0.22	0.06	0.30	0.18	0.06	0.20	0.15	0.11	0.40	0.24	0.03	0.35	0.19	0.07	0.30	0.18	-	-	-	-	-	-	0.03	0.40	0.19
M8-FA-16-(D)	1st	18.2	0.17	0.03	0.03	0.03	0.03	0.05	0.04	-	-	-	-	-	-	-	-	-	-	-	-	-	-	-	-	-	-	0.03	0.05	0.03
	2nd	19.3	0.18	0.03	0.04	0.04	0.04	0.06	0.05	0.03	0.05	0.04	0.04	0.05	0.04	-	-	-	-	-	-	-	-	-	-	-	-	0.03	0.06	0.04
	3rd	22.3	0.21	0.05	0.06	0.06	0.05	0.07	0.06	0.05	0.07	0.06	0.02	0.11	0.06	0.04	0.09	0.07	-	-	-	-	-	-	-	-	-	0.02	0.11	0.06
	4th	33.8	0.31	0.07	0.09	0.08	0.04	0.12	0.07	0.10	0.12	0.11	0.04	0.13	0.10	0.08	0.15	0.10	0.04	0.06	0.05	-	-	-	-	-	-	0.04	0.15	0.09
	5th	50.1	0.47	0.11	0.14	0.12	0.03	0.19	0.11	0.18	0.21	0.20	0.08	0.20	0.16	0.09	0.19	0.15	0.07	0.13	0.10	-	-	-	-	-	-	0.03	0.21	0.14
	6th	57.3	0.53	0.12	0.17	0.15	0.06	0.21	0.14	0.21	0.25	0.24	0.13	0.21	0.17	0.09	0.22	0.14	0.10	0.14	0.12	0.03	0.06	0.05	-	-	-	0.03	0.25	0.14
	7th	72.4	0.67	0.15	0.22	0.18	0.06	0.25	0.16	0.25	0.31	0.27	0.04	0.24	0.19	0.16	0.26	0.21	0.16	0.24	0.19	0.05	0.07	0.06	-	-	-	0.04	0.31	0.18
	8th	87.1	0.81	0.08	0.27	0.21	0.07	0.31	0.18	0.30	0.37	0.32	0.10	0.25	0.19	0.04	0.27	0.19	0.18	0.28	0.24	0.06	0.08	0.08	-	-	-	0.04	0.37	0.19
	9th	98.0	0.91	0.04	0.28	0.19	0.11	0.33	0.22	0.04	0.40	0.27	0.06	0.26	0.17	0.05	0.29	0.22	0.03	0.32	0.19	0.06	0.09	0.07	-	-	-	0.03	0.40	0.17
M9-FA-10-(A)	1st	9.3	0.22	0.06	0.08	0.07	0.06	0.11	0.07	-	-	-	-	-	-	-	-	-	-	-	-	-	-	-	-	-	-	0.06	0.11	0.07
	2nd	11.7	0.28	0.07	0.12	0.10	0.06	0.16	0.11	0.06	0.08	0.07	-	-	-	-	-	-	-	-	-	-	-	-	-	-	-	0.06	0.16	0.09
	3rd	13.5	0.32	0.08	0.16	0.11	0.05	0.17	0.11	0.06	0.13	0.09	0.04	0.07	0.05	-	-	-	-	-	-	-	-	-	-	-	-	0.04	0.17	0.09
	4th	17.2	0.41	0.05	0.21	0.13	0.05	0.27	0.14	0.06	0.19	0.13	0.00	0.10	0.05	0.06	0.09	0.08	-	-	-	-	-	-	-	-	-	0.00	0.27	0.11
	5th	21.0	0.50	0.06	0.24	0.14	0.05	0.36	0.18	0.10	0.22	0.17	0.07	0.14	0.11	0.07	0.14	0.11	0.08	0.12	0.10	-	-	-	-	-	-	0.05	0.36	0.13
	6th	31.0	0.74	0.08	0.36	0.22	0.06	0.46	0.22	0.09	0.36	0.22	0.08	0.26	0.18	0.04	0.24	0.14	0.14	0.22	0.17	-	-	-	-	-	-	0.04	0.46	0.19
	7th	41.6	0.99	0.08	0.46	0.27	0.10	0.69	0.30	0.09	0.47	0.27	0.08	0.32	0.21	0.04	0.34	0.19	0.22	0.30	0.25	-	-	-	-	-	-	0.04	0.69	0.25
M9-FA-10-(B)	1st	9.0	0.22	0.05	0.06	0.06	-	-	-	-	-	-	-	-	-	-	-	-	-	-	-	-	-	-	-	-	-	0.05	0.06	0.06
	2nd	11.3	0.27	0.06	0.08	0.07	0.03	0.05	0.04	-	-	-	-	-	-	-	-	-	-	-	-	-	-	-	-	-	-	0.03	0.08	0.05
	3rd	13.0	0.31	0.05	0.09	0.07	0.04	0.06	0.05	0.04	0.08	0.06	0.05	0.10	0.07	0.08	0.14	0.11	0.05	0.15	0.09	-	-	-	-	-	-	0.04	0.15	0.08
	4th	17.3	0.41	0.05	0.10	0.07	0.04	0.13	0.06	0.05	0.11	0.08	0.05	0.19	0.09	0.19	0.25	0.23	0.07	0.21	0.14	0.10	0.12	0.11	-	-	-	0.04	0.25	0.11

Spec. ID.	Sta. NO.	P (kN)	(σ/Fy)	1st crack			2nd crack			3rd crack			4th crack			5th crack			6th crack			7th crack			8th crack			Min. crack width	Max. crack width	Ave. crack width
				Min.	Max.	Ave.	Min.	Max.	Ave.	Min.	Max.	Ave.	Min.	Max.	Ave.	Min.	Max.	Ave.	Min.	Max.	Ave.	Min.	Max.	Ave.	Min.	Max.	Ave.	Min.	Max.	Ave.
	5th	27.1	0.64	0.06	0.11	0.07	0.08	0.26	0.15	0.09	0.12	0.10	0.06	0.34	0.20	0.30	0.38	0.35	0.11	0.26	0.18	0.16	0.29	0.22	-	-	-	0.06	0.38	0.18
	6th	37.0	0.88	0.07	0.14	0.09	0.25	0.30	0.27	0.11	0.16	0.14	0.15	0.43	0.26	0.41	0.48	0.44	0.06	0.35	0.18	0.30	0.37	0.33	-	-	-	0.06	0.48	0.25
	7th	44.0	1.05	0.08	0.15	0.11	0.05	0.36	0.27	0.16	0.20	0.18	0.23	0.51	0.33	0.05	0.58	0.41	0.15	0.36	0.24	0.33	0.42	0.38	-	-	-	0.05	0.58	0.28
M9-FA-10-(C)	1st	9.2	0.22	0.05	0.05	0.05	0.06	0.07	0.07	0.06	0.11	0.09	-	-	-	-	-	-	-	-	-	-	-	-	-	-	-	0.05	0.11	0.07
	2nd	11.7	0.28	0.07	0.12	0.10	0.06	0.11	0.08	0.11	0.18	0.14	0.05	0.05	0.05	0.05	0.06	0.05	-	-	-	-	-	-	-	-	-	0.05	0.18	0.08
	3rd	16.6	0.40	0.12	0.19	0.16	0.13	0.23	0.16	0.13	0.26	0.18	0.09	0.12	0.10	0.11	0.17	0.13	0.05	0.05	0.05	-	-	-	-	-	-	0.05	0.26	0.13
	4th	26.7	0.64	0.19	0.26	0.23	0.30	0.37	0.33	0.16	0.42	0.26	0.19	0.26	0.22	0.17	0.35	0.26	0.07	0.25	0.17	-	-	-	-	-	-	0.07	0.42	0.25
	5th	36.6	0.87	0.17	0.33	0.28	0.06	0.50	0.33	0.08	0.54	0.31	0.06	0.36	0.28	0.08	0.47	0.27	0.12	0.35	0.23	-	-	-	-	-	-	0.06	0.54	0.28
	6th	41.0	0.98	0.23	0.37	0.32	0.04	0.51	0.27	0.09	0.71	0.35	0.09	0.39	0.29	0.08	0.52	0.29	0.12	0.41	0.27	-	-	-	-	-	-	0.04	0.71	0.30
M9-FA-10-(D)	1st	7.6	0.18	0.05	0.07	0.06	0.06	0.07	0.07	-	-	-	-	-	-	-	-	-	-	-	-	-	-	-	-	-	-	0.05	0.07	0.06
	2nd	9.6	0.23	0.10	0.15	0.13	0.10	0.15	0.13	0.04	0.08	0.06	-	-	-	-	-	-	-	-	-	-	-	-	-	-	-	0.04	0.15	0.11
	3rd	18.3	0.44	0.27	0.38	0.34	0.23	0.38	0.29	0.06	0.28	0.18	0.06	0.11	0.08	-	-	-	-	-	-	-	-	-	-	-	-	0.06	0.38	0.22
	4th	23.9	0.57	0.43	0.49	0.46	0.37	0.55	0.46	0.06	0.39	0.28	0.07	0.12	0.09	0.08	0.09	0.08	0.06	0.11	0.09	-	-	-	-	-	-	0.06	0.55	0.24
	5th	34.0	0.81	0.12	0.72	0.58	0.09	0.66	0.47	0.05	0.50	0.39	0.13	0.19	0.11	0.06	0.22	0.14	0.15	0.20	0.18	-	-	-	-	-	-	0.05	0.72	0.31
	6th	42.1	1.00	0.13	0.77	0.63	0.09	0.78	0.53	0.09	0.63	0.09	0.05	0.30	0.17	0.07	0.34	0.19	0.22	0.26	0.24	-	-	-	-	-	-	0.05	0.78	0.31
M9-FA-12-(A)	1st	11.8	0.19	0.05	0.06	0.05	-	-	-	-	-	-	-	-	-	-	-	-	-	-	-	-	-	-	-	-	-	0.05	0.06	0.05
	2nd	12.4	0.21	0.03	0.07	0.06	0.05	0.05	0.05	-	-	-	-	-	-	-	-	-	-	-	-	-	-	-	-	-	-	0.03	0.07	0.05
	3rd	14.5	0.24	0.06	0.11	0.08	0.06	0.07	0.06	0.03	0.04	0.04	0.04	0.06	0.05	-	-	-	-	-	-	-	-	-	-	-	-	0.03	0.11	0.06
	4th	15.9	0.26	0.07	0.14	0.10	0.06	0.08	0.07	0.03	0.05	0.04	0.04	0.07	0.06	0.05	0.07	0.06	-	-	-	-	-	-	-	-	-	0.03	0.14	0.07
	5th	19.1	0.32	0.09	0.16	0.12	0.03	0.12	0.07	0.05	0.09	0.06	0.05	0.10	0.07	0.07	0.11	0.09	0.06	0.06	0.06	-	-	-	-	-	-	0.03	0.16	0.08
	6th	23.1	0.38	0.05	0.22	0.13	0.04	0.16	0.11	0.07	0.12	0.09	0.05	0.14	0.09	0.10	0.17	0.13	0.06	0.07	0.06	0.05	0.07	0.06	-	-	-	0.04	0.22	0.10
	7th	33.1	0.55	0.04	0.31	0.18	0.07	0.25	0.15	0.11	0.20	0.16	0.05	0.21	0.13	0.17	0.23	0.19	0.08	0.09	0.09	0.06	0.17	0.12	-	-	-	0.04	0.31	0.15
	8th	43.1	0.71	0.05	0.36	0.24	0.05	0.28	0.18	0.05	0.26	0.20	0.06	0.32	0.20	0.20	0.34	0.26	0.10	0.16	0.13	0.07	0.24	0.13	-	-	-	0.05	0.36	0.19
	9th	55.9	0.92	0.04	0.41	0.29	0.04	0.35	0.22	0.09	0.36	0.22	0.08	0.36	0.24	0.05	0.52	0.24	0.05	0.23	0.13	0.06	0.34	0.23	-	-	-	0.04	0.52	0.22
M9-FA-12-(B)	1st	12.8	0.21	0.05	0.05	0.05	0.05	0.07	0.06	0.05	0.06	0.05	-	-	-	-	-	-	-	-	-	-	-	-	-	-	-	0.05	0.07	0.05
	2nd	15.0	0.25	0.03	0.07	0.05	0.06	0.09	0.07	0.05	0.08	0.06	0.03	0.06	0.04	-	-	-	-	-	-	-	-	-	-	-	-	0.03	0.09	0.06
	3rd	18.2	0.30	0.04	0.09	0.07	0.07	0.12	0.09	0.06	0.11	0.08	0.04	0.09	0.06	0.04	0.06	0.05	0.07	0.08	0.07	-	-	-	-	-	-	0.04	0.12	0.07
	4th	27.1	0.45	0.06	0.12	0.08	0.06	0.18	0.14	0.10	0.21	0.16	0.03	0.19	0.11	0.12	0.17	0.14	0.08	0.15	0.11	-	-	-	-	-	-	0.03	0.21	0.12
	5th	37.0	0.61	0.06	0.19	0.13	0.14	0.27	0.20	0.07	0.29	0.18	0.03	0.23	0.12	0.15	0.21	0.18	0.10	0.18	0.14	-	-	-	-	-	-	0.03	0.29	0.16
	6th	47.2	0.78	0.05	0.27	0.14	0.08	0.32	0.19	0.08	0.40	0.24	0.05	0.27	0.15	0.17	0.29	0.23	0.14	0.27	0.23	-	-	-	-	-	-	0.05	0.40	0.20
	7th	55.6	0.92	0.06	0.35	0.20	0.06	0.35	0.20	0.05	0.54	0.29	0.04	0.31	0.14	0.21	0.35	0.27	0.15	0.33	0.29	-	-	-	-	-	-	0.04	0.54	0.23
M9-FA-16-(A)	1st	12.4	0.12	0.02	0.10	0.05	-	-	-	-	-	-	-	-	-	-	-	-	-	-	-	-	-	-	-	-	-	0.02	0.10	0.05
	2nd	14.2	0.13	0.06	0.11	0.08	0.04	0.08	0.06	-	-	-	-	-	-	-	-	-	-	-	-	-	-	-	-	-	-	0.04	0.11	0.07
	3rd	18.4	0.17	0.05	0.15	0.09	0.05	0.12	0.07	0.04	0.06	0.05	0.06	0.07	0.07	-	-	-	-	-	-	-	-	-	-	-	-	0.04	0.15	0.07
	4th	31.3	0.29	0.09	0.20	0.13	0.04	0.24	0.15	0.05	0.15	0.10	0.06	0.14	0.09	0.08	0.08	0.08	-	-	-	-	-	-	-	-	-	0.04	0.24	0.11
	5th	41.3	0.38	0.09	0.26	0.18	0.09	0.29	0.19	0.06	0.21	0.14	0.05	0.19	0.11	0.08	0.13	0.11	0.08	0.11	0.09	-	-	-	-	-	-	0.05	0.29	0.14
	6th	50.2	0.47	0.10	0.30	0.19	0.06	0.35	0.21	0.08	0.23	0.14	0.04	0.20	0.11	0.10	0.17	0.13	0.10	0.13	0.11	0.04	0.10	0.06	-	-	-	0.04	0.35	0.14
	7th	64.5	0.60	0.12	0.34	0.20	0.13	0.42	0.26	0.07	0.32	0.21	0.04	0.22	0.17	0.14	0.21	0.18	0.07	0.14	0.11	0.10	0.17	0.13	-	-	-	0.04	0.42	0.18

Spec. ID.	Sta. NO.	P (kN)	(σ/Fy)	1st crack			2nd crack			3rd crack			4th crack			5th crack			6th crack			7th crack			8th crack			Min. crack width	Max. crack width	Ave. crack width
				Min.	Max.	Ave.	Min.	Max.	Ave.	Min.	Max.	Ave.	Min.	Max.	Ave.	Min.	Max.	Ave.	Min.	Max.	Ave.	Min.	Max.	Ave.	Min.	Max.	Ave.	Min.	Max.	Ave.
	8th	80.1	0.75	0.12	0.40	0.26	0.14	0.43	0.29	0.10	0.35	0.23	0.04	0.29	0.19	0.14	0.22	0.19	0.04	0.23	0.13	0.09	0.25	0.17	-	-	-	0.04	0.43	0.21
	9th	98.6	0.92	0.05	0.42	0.25	0.06	0.45	0.31	0.05	0.39	0.23	0.05	0.30	0.21	0.06	0.23	0.18	0.07	0.24	0.15	0.10	0.34	0.23	-	-	-	0.05	0.45	0.22
M9-FA-16-(B)	1st	24.9	0.23	0.04	0.08	0.06	-	-	-	-	-	-	-	-	-	-	-	-	-	-	-	-	-	-	-	-	-	0.04	0.08	0.06
	2nd	31.3	0.29	0.04	0.11	0.08	0.06	0.07	0.07	0.07	0.09	0.08	0.05	0.10	0.08	0.05	0.08	0.07	-	-	-	-	-	-	-	-	-	0.04	0.11	0.07
	3rd	34.8	0.32	0.09	0.13	0.10	0.04	0.08	0.06	0.07	0.10	0.09	0.05	0.12	0.09	0.06	0.09	0.07	0.05	0.06	0.05	-	-	-	-	-	-	0.04	0.13	0.08
	4th	39.3	0.37	0.11	0.14	0.12	0.06	0.09	0.07	0.05	0.12	0.09	0.08	0.13	0.11	0.06	0.11	0.08	0.05	0.08	0.06	0.07	0.07	0.07	-	-	-	0.05	0.14	0.09
	5th	55.0	0.51	0.13	0.18	0.16	0.06	0.11	0.09	0.10	0.18	0.14	0.10	0.16	0.13	0.03	0.18	0.11	0.08	0.15	0.10	0.06	0.12	0.10	0.07	0.11	0.09	0.03	0.18	0.11
	6th	70.0	0.65	0.15	0.24	0.19	0.06	0.15	0.11	0.10	0.20	0.14	0.08	0.18	0.16	0.05	0.21	0.15	0.05	0.18	0.13	0.08	0.17	0.13	0.05	0.14	0.11	0.05	0.24	0.14
	7th	85.4	0.80	0.07	0.26	0.19	0.09	0.16	0.13	0.04	0.22	0.13	0.06	0.24	0.18	0.05	0.22	0.17	0.08	0.21	0.15	0.06	0.22	0.14	0.05	0.20	0.13	0.04	0.26	0.15
	8th	99.0	0.92	0.09	0.34	0.27	0.08	0.18	0.14	0.07	0.33	0.19	0.06	0.32	0.18	0.06	0.25	0.17	0.13	0.23	0.17	0.06	0.27	0.15	0.04	0.24	0.15	0.04	0.34	0.18
M9-FA-16-(C)	1st	18.7	0.17	0.03	0.07	0.05	0.04	0.05	0.05	-	-	-	-	-	-	-	-	-	-	-	-	-	-	-	-	-	-	0.03	0.07	0.05
	2nd	20.8	0.19	0.04	0.08	0.06	0.05	0.06	0.05	0.03	0.06	0.05	-	-	-	-	-	-	-	-	-	-	-	-	-	-	-	0.03	0.08	0.05
	3rd	23.0	0.21	0.06	0.09	0.07	0.04	0.07	0.06	0.04	0.07	0.06	0.04	0.06	0.05	-	-	-	-	-		-	-	-	-	-	-	0.04	0.09	0.06
	4th	27.3	0.25	0.09	0.10	0.09	0.04	0.10	0.08	0.04	0.08	0.07	0.05	0.08	0.06	0.04	0.05	0.05	-	-		-	-		-	-	-	0.04	0.10	0.07
	5th	42.1	0.39	0.15	0.18	0.16	0.07	0.20	0.13	0.09	0.17	0.13	0.05	0.14	0.09	0.05	0.07	0.06	-	-		-	-		-	-	-	0.05	0.20	0.12
	6th	57.0	0.53	0.18	0.23	0.21	0.08	0.23	0.16	0.03	0.24	0.14	0.09	0.21	0.13	0.04	0.08	0.06	0.03	0.09	0.07	0.04	0.12	0.08	-	-	-	0.03	0.24	0.12
	7th	72.3	0.67	0.20	0.29	0.26	0.07	0.27	0.18	0.18	0.29	0.23	0.05	0.25	0.15	0.04	0.11	0.07	0.05	0.14	0.09	0.05	0.19	0.11	-	-	-	0.04	0.29	0.16
	8th	87.1	0.81	0.28	0.35	0.30	0.11	0.31	0.19	0.04	0.30	0.16	0.06	0.26	0.17	0.05	0.16	0.10	0.04	0.18	0.11	0.07	0.22	0.14	-	-	-	0.04	0.35	0.17
	9th	96.0	0.89	0.26	0.46	0.36	0.04	0.35	0.20	0.06	0.33	0.18	0.09	0.28	0.20	0.06	0.17	0.10	0.06	0.20	0.12	0.05	0.28	0.17	-	-	-	0.04	0.46	0.19
M9-FA-16-(D)	1st	17.8	0.17	0.02	0.02	0.02	0.04	0.05	0.04	-	-	-	-	-	-	-	-	-	-	-	-	-	-	-	-	-	-	0.02	0.05	0.03
	2nd	23.0	0.21	0.03	0.07	0.05	0.06	0.06	0.06	0.03	0.04	0.03	-	-	-	-	-	-	-	-	-	-	-	-	-	-	-	0.03	0.07	0.05
	3rd	24.1	0.22	0.04	0.08	0.06	0.04	0.09	0.06	0.04	0.05	0.04	0.04	0.10	0.07	0.04	0.05	0.04	-	-	-	-	-	-	-	-	-	0.04	0.10	0.06
	4th	27.3	0.25	0.03	0.09	0.06	0.07	0.08	0.07	0.06	0.07	0.06	0.06	0.13	0.09	0.05	0.08	0.06	0.05	0.09	0.07	-	-	-	-	-	-	0.03	0.13	0.07
	5th	42.1	0.39	0.10	0.17	0.12	0.05	0.17	0.11	0.06	0.14	0.10	0.10	0.19	0.15	0.05	0.13	0.10	0.07	0.13	0.10	-	-	-	-	-	-	0.05	0.19	0.11
	6th	57.1	0.53	0.06	0.23	0.13	0.05	0.22	0.15	0.05	0.15	0.10	0.18	0.25	0.21	0.03	0.20	0.12	0.05	0.18	0.14	-	-	-	-	-	-	0.03	0.25	0.14
	7th	72.2	0.67	0.04	0.24	0.13	0.06	0.29	0.19	0.05	0.24	0.15	0.26	0.31	0.28	0.03	0.26	0.14	0.13	0.21	0.18	-	-	-	-	-	-	0.03	0.31	0.18
	8th	87.1	0.81	0.07	0.26	0.16	0.10	0.32	0.21	0.07	0.27	0.16	0.27	0.38	0.33	0.07	0.29	0.17	0.06	0.24	0.21	-	-	-	-	-	-	0.06	0.38	0.21
	9th	91.3	0.85	0.10	0.27	0.16	0.02	0.33	0.17	0.04	0.28	0.15	0.05	0.41	0.23	0.05	0.31	0.17	0.05	0.28	0.21	-	-	-	-	-	-	0.02	0.41	0.18
M1-OPC -12 (A)	1st	10.5	0.17	0.07	0.10	0.08	-	-	-	-	-	-	-	-	-	-	-	-	-	-	-	-	-	-	-	-	-	0.07	0.10	0.08
	2nd	16.1	0.27	0.15	0.19	0.17	0.07	0.15	0.11	-	-	-	-	-	-	-	-	-	-	-	-	-	-	-	-	-	-	0.07	0.19	0.14
	3rd	17.2	0.28	0.13	0.20	0.17	0.10	0.17	0.14	0.03	0.19	0.12	-	-	-	-	-	-	-	-	-	-	-	-	-	-	-	0.03	0.20	0.14
	4th	21.4	0.35	0.16	0.24	0.21	0.15	0.24	0.20	0.06	0.26	0.16	0.12	0.20	0.16	-	-	-	-	-	-	-	-	-	-	-	-	0.06	0.26	0.18
	5th	35.1	0.58	0.05	0.36	0.26	0.03	0.36	0.24	0.04	0.36	0.18	0.04	0.34	0.23	-	-	-	-	-	-	-	-	-	-	-	-	0.03	0.36	0.21
	6th	45.0	0.75	0.18	0.43	0.26	0.04	0.48	0.32	0.11	0.37	0.25	0.10	0.39	0.23	-	-	-	-	-	-	-	-	-	-	-	-	0.04	0.48	0.26
	7th	49.9	0.83	0.22	0.62	0.31	0.07	0.51	0.37	0.18	0.41	0.28	0.12	0.86	0.38	-	-	-	-	-	-	-	-	-	-	-	-	0.07	0.86	0.36
M1-OPC -12(B)	1st	15.1	0.25	0.03	0.24	0.16	-	-	-	-	-	-	-	-	-	-	-	-	-	-	-	-	-	-	-	-	-	0.03	0.24	0.16
	2nd	18.5	0.31	0.03	0.30	0.18	0.04	0.22	0.14	-	-	-	-	-	-	-	-	-	-	-	-	-	-	-	-	-	-	0.03	0.30	0.16
	3rd	32.6	0.54	0.13	0.34	0.23	0.19	0.28	0.22	-	-	-	-	-	-	-	-	-	-	-	-	-	-	-	-	-	-	0.13	0.34	0.23

Spec. ID.	Sta. NO.	P (kN)	(σ/Fy)	1st crack			2nd crack			3rd crack			4th crack			5th crack			6th crack			7th crack			8th crack			Min. crack width	Max. crack width	Ave. crack width
				Min.	Max.	Ave.	Min.	Max.	Ave.	Min.	Max.	Ave.	Min.	Max.	Ave.	Min.	Max.	Ave.	Min.	Max.	Ave.	Min.	Max.	Ave.	Min.	Max.	Ave.	Min.	Max.	Ave.
	4th	42.7	0.71	0.25	0.39	0.31	0.20	0.45	0.31	-	-	-	-	-	-	-	-	-	-	-	-	-	-	-	-	-	-	0.20	0.45	0.31
	5th	44.2	0.73	0.21	0.38	0.29	0.20	0.51	0.33	0.03	0.24	0.14	-	-	-	-	-	-	-	-	-	-	-	-	-	-	-	0.03	0.51	0.25
	6th	54.6	0.90	0.24	0.91	0.38	0.23	0.62	0.44	0.07	0.37	0.22	-	-	-	-	-	-	-	-	-	-	-	-	-	-	-	0.07	0.91	0.35
M2-OPC -16(A)	1st	11.7	0.11	0.04	0.06	0.05	0.04	0.08	0.06	-	-	-	-	-	-	-	-	-	-	-	-	-	-	-	-	-	-	0.04	0.08	0.06
	2nd	13.0	0.12	0.05	0.08	0.06	0.05	0.10	0.07	0.03	0.04	0.04	-	-	-	-	-	-	-	-	-	-	-	-	-	-	-	0.03	0.10	0.06
	3rd	17.5	0.16	0.07	0.11	0.09	0.04	0.14	0.09	0.07	0.08	0.07	0.04	0.08	0.06	-	-	-	-	-	-	-	-	-	-	-	-	0.04	0.14	0.08
	4th	23.2	0.22	0.10	0.14	0.12	0.03	0.17	0.10	0.06	0.10	0.09	0.05	0.11	0.08	0.04	0.08	0.07	-	-	-	-	-	-	-	-	-	0.03	0.17	0.09
	5th	40.3	0.38	0.14	0.20	0.17	0.02	0.30	0.18	0.05	0.17	0.12	0.05	0.18	0.13	0.04	0.17	0.11	-	-	-	-	-	-	-	-	-	0.02	0.30	0.14
	6th	55.7	0.52	0.04	0.23	0.14	0.04	0.40	0.23	0.08	0.26	0.16	0.04	0.24	0.12	0.08	0.22	0.16	-	-	-	-	-	-	-	-	-	0.04	0.40	0.16
	7th	70.4	0.66	0.14	0.25	0.17	0.08	0.50	0.27	0.07	0.28	0.17	0.07	0.28	0.17	0.06	0.27	0.18	-	-	-	-	-	-	-	-	-	0.06	0.50	0.19
	8th	85.0	0.79	0.17	0.26	0.20	0.05	0.54	0.28	0.06	0.34	0.20	0.09	0.31	0.19	0.07	0.29	0.18	-	-	-	-	-	-	-	-	-	0.05	0.54	0.21
	9th	99.1	0.92	0.19	0.30	0.24	0.09	0.55	0.32	0.08	0.73	0.27	0.08	0.32	0.21	0.11	0.34	0.21	-	-	-	-	-	-	-	-	-	0.08	0.73	0.25
M2-OPC -16(B)	1st	13.4	0.13	0.06	0.07	0.06	0.04	0.07	0.05	-	-	-	-	-	-	-	-	-	-	-	-	-	-	-	-	-	-	0.04	0.07	0.06
	2nd	15.8	0.15	0.05	0.08	0.07	0.05	0.08	0.07	0.05	0.07	0.06	-	-	-	-	-	-	-	-	-	-	-	-	-	-	-	0.05	0.08	0.06
	3rd	18.0	0.17	0.08	0.10	0.09	0.06	0.09	0.08	0.06	0.08	0.07	0.04	0.08	0.06	0.07	0.10	0.09	-	-	-	-	-	-	-	-	-	0.04	0.10	0.08
	4th	33.2	0.31	0.11	0.20	0.15	0.04	0.20	0.11	0.12	0.15	0.13	0.05	0.18	0.11	0.17	0.23	0.19	-	-	-	-	-	-	-	-	-	0.04	0.23	0.14
	5th	48.1	0.45	0.02	0.25	0.12	0.05	0.26	0.13	0.11	0.21	0.15	0.05	0.27	0.15	0.04	0.58	0.23	-	-	-	-	-	-	-	-	-	0.02	0.58	0.16
	6th	63.2	0.59	0.11	0.33	0.17	0.06	0.27	0.16	0.09	0.22	0.14	0.07	0.33	0.19	0.05	0.59	0.23	0.05	0.11	0.09	-	-	-	-	-	-	0.05	0.59	0.16
	7th	78.2	0.73	0.16	0.37	0.22	0.10	0.28	0.18	0.11	0.23	0.17	0.04	0.36	0.18	0.06	0.61	0.24	0.08	0.16	0.13	-	-	-	-	-	-	0.04	0.61	0.19
	8th	96.2	0.90	0.17	0.56	0.28	0.10	0.32	0.22	0.13	0.26	0.19	0.11	0.48	0.25	0.08	0.68	0.31	0.14	0.17	0.16	-	-	-	-	-	-	0.08	0.68	0.23

*All crack width measurements are in mm.

Table S5 Crack width regression analysis for each specimen.

Specimen	Crack widths (mm)		
	Maximum	Average	Minimum
M5-FA-10-(A)	w_c =0.25ln(σ_s/F_y)+ 0.54<br>R^2 = 0.99	w_c =0.14ln(σ_s/F_y)+ 0.28<br>R^2 = 0.96	w_c =0.002ln(σ_s/F_y)+ 0.03877<br>R^2 = 0.05882
M5-FA-10-(B)	w_c =0.35ln(σ_s/F_y)+ 0.55<br>R^2 = 0.98	w_c =0.15ln(σ_s/F_y) + 0.25<br>R^2 = 0.98	w_c =-0.0004ln(σ_s/F_y) + 0.02860<br>R^2 = 0.00150
M5-FA-10-(C)	w_c =0.32ln(σ_s/F_y) + 0.47<br>R^2 = 1.00	w_c =0.16ln(σ_s/F_y)+ 0.24<br>R^2 = 0.98	w_c =0.02ln(σ_s/F_y) + 0.06<br>R^2 = 0.54
M5-FA-10-(D)	w_c =0.29ln(σ_s/F_y)+ 0.47<br>R^2 = 0.96	w_c =0.16ln(σ_s/F_y) + 0.26<br>R^2 = 0.99	w_c =0.005ln(σ_s/F_y)+ 0.04<br>R^2 = 0.01
M5-FA-12-(A)	w_c =0.21ln(σ_s/F_y) + 0.44<br>R^2 = 0.97	w_c =0.10ln(σ_s/F_y) + 0.20<br>R^2 = 0.92	w_c =0.01ln(σ_s/F_y)+ 0.04<br>R^2 = 0.07
M5-FA-12-(B)	w_c =0.28ln(σ_s/F_y) + 0.53<br>R^2 = 0.98	w_c =0.15ln(σ_s/F_y)+ 0.29<br>R^2 = 0.99	w_c =0.0009ln(σ_s/F_y)+ 0.0435<br>R^2 = 0.0047
M5-FA-12-(C)	w_c =0.26ln(σ_s/F_y)+ 0.45<br>R^2 = 0.98	w_c =0.14ln(σ_s/F_y)+ 0.25<br>R^2 = 0.98	w_c =-0.001ln(σ_s/F_y)+ 0.0420<br>R^2 = 0.0053
M5-FA-12-(D)	w_c =0.30ln(σ_s/F_y) + 0.54<br>R^2 = 0.98	w_c =0.09ln(σ_s/F_y) + 0.19<br>R^2 = 0.97	w_c =-0.006ln(σ_s/F_y) + 0.0277<br>R^2 = 0.3997
M5-FA-16-(A)	w_c =0.21ln(σ_s/F_y)+ 0.42<br>R^2 = 0.96	w_c =0.10ln(σ_s/F_y)+ 0.21<br>R^2 = 0.91	w_c =0.0051ln(σ_s/F_y)+ 0.0416<br>R^2 = 0.1443
M5-FA-16-(B)	w_c =0.23ln(σ_s/F_y) + 0.45<br>R^2 = 0.98	w_c =0.08ln(σ_s/F_y)+ 0.18<br>R^2 = 1.00	w_c =0.001ln(σ_s/F_y)+ 0.0278<br>R^2 = 0.0172
M5-FA-16-(C)	w_c =0.20ln(σ_s/F_y)+ 0.45<br>R^2 = 0.99	w_c =0.07ln(σ_s/F_y)+ 0.17<br>R^2 = 0.99	w_c =0.004ln(σ_s/F_y)+ 0.0361<br>R^2 = 0.1085
M5-FA-16-(D)	w_c =0.22ln(σ_s/F_y)+ 0.40<br>R^2 = 1.00	w_c =0.09ln(σ_s/F_y)+ 0.19<br>R^2 = 0.99	w_c =-0.002ln(σ_s/F_y)+ 0.032<br>R^2 = 0.037
M8-FA-10-(A)	w_c =0.21ln(σ_s/F_y)+ 0.59<br>R^2 = 0.89	w_c =0.10ln(σ_s/F_y)+ 0.30<br>R^2 = 0.85	w_c =0.003ln(σ_s/F_y)+ 0.036<br>R^2 = 0.061
M8-FA-10-(B)	w_c =0.36ln(σ_s/F_y)+ 0.72<br>R^2 = 0.99	w_c =0.19ln(σ_s/F_y)+ 0.42<br>R^2 = 0.98	w_c =-0.03ln(σ_s/F_y)+ 0.02<br>R^2 = 0.37
M8-FA-10-(C)	w_c =0.38ln(σ_s/F_y)+ 0.58<br>R^2 = 0.96	w_c =0.18ln(σ_s/F_y)+ 0.29<br>R^2 = 0.90	w_c =0.003ln(σ_s/F_y)+ 0.041<br>R^2 = 0.079
M8-FA-10-(D)	w_c =0.38ln(σ_s/F_y)+ 0.62<br>R^2 = 0.96	w_c =0.22ln(σ_s/F_y)+ 0.36<br>R^2 = 0.91	w_c =0.13ln(σ_s/F_y)+ 0.21<br>R^2 = 0.84
M8-FA-12-(A)	w_c =0.32ln(σ_s/F_y)+ 0.62<br>R^2 = 0.99	w_c =0.12ln(σ_s/F_y)+ 0.26<br>R^2 = 0.98	w_c =0.01ln(σ_s/F_y)+ 0.06<br>R^2 = 0.33
M8-FA-12-(B)	w_c =0.23ln(σ_s/F_y)+ 0.08<br>R^2 = 0.99	w_c =0.08ln(σ_s/F_y)+ 0.05<br>R^2 = 0.90	w_c =0.003ln(σ_s/F_y)+ 0.027<br>R^2 = 0.055
M8-FA-12-(C)	w_c =0.27ln(σ_s/F_y)+ 0.48<br>R^2 = 0.98	w_c =0.13ln(σ_s/F_y)+ 0.24<br>R^2 = 0.98	w_c =0.02ln(σ_s/F_y)+ 0.05<br>R^2 = 0.70
M8-FA-12-(D)	w_c =0.30ln(σ_s/F_y)+ 0.52<br>R^2 = 0.98	w_c =0.11ln(σ_s/F_y)+ 0.22<br>R^2 = 0.98	w_c =0.0004ln(σ_s/F_y)+ 0.0397<br>R^2 = 0.0016
M8-FA-16-(A)	w_c =0.20ln(σ_s/F_y)+ 0.48<br>R^2 = 0.91	w_c =0.08ln(σ_s/F_y)+ 0.20<br>R^2 = 0.89	w_c =0.001ln(σ_s/F_y)+ 0.035<br>R^2 = 0.006
M8-FA-16-(B)	w_c =0.20ln(σ_s/F_y)+ 0.45<br>R^2 = 0.99	w_c =0.08ln(σ_s/F_y)+ 0.22<br>R^2 = 1.00	w_c =-0.004ln(σ_s/F_y)+ 0.031<br>R^2 = 0.346
M8-FA-16-(C)	w_c =0.20ln(σ_s/F_y)+ 0.41<br>R^2 = 0.99	w_c =0.09ln(σ_s/F_y)+ 0.19<br>R^2 = 0.98	w_c =0.01ln(σ_s/F_y)+ 0.04<br>R^2 = 0.28
M8-FA-16-(D)	w_c =0.19ln(σ_s/F_y)+ 0.39<br>R^2 = 0.98	w_c =0.09ln(σ_s/F_y)+ 0.20<br>R^2 = 0.98	w_c =0.01ln(σ_s/F_y)+ 0.04<br>R^2 = 0.31
M9-FA-10-(A)	w_c =0.38ln(σ_s/F_y)+ 0.63	w_c =0.12ln(σ_s/F_y)+ 0.23	w_c =-0.01ln(σ_s/F_y)+ 0.04

Specimen	Crack widths (mm)		
	Maximum	Average	Minimum
	$R^2 = 0.96$	$R^2 = 0.94$	$R^2 = 0.03$
M9-FA-10-(B)	$w_c = 0.33\ln(\sigma_s/F_y) + 0.54$	$w_c = 0.15\ln(\sigma_s/F_y) + 0.26$	$w_c = 0.01\ln(\sigma_s/F_y) + 0.06$
	$R^2 = 0.99$	$R^2 = 0.97$	$R^2 = 0.48$
M9-FA-10-(C)	$w_c = 0.36\ln(\sigma_s/F_y) + 0.63$	$w_c = 0.17\ln(\sigma_s/F_y) + 0.31$	$w_c = 0.01\ln(\sigma_s/F_y) + 0.06$
	$R^2 = 0.94$	$R^2 = 0.98$	$R^2 = 0.09$
M9-FA-10-(D)	$w_c = 0.43\ln(\sigma_s/F_y) + 0.78$	$w_c = 0.15\ln(\sigma_s/F_y) + 0.33$	$w_c = 0.004\ln(\sigma_s/F_y) + 0.056$
	$R^2 = 0.99$	$R^2 = 0.98$	$R^2 = 0.098$
M9-FA-12-(A)	$w_c = 0.27\ln(\sigma_s/F_y) + 0.49$	$w_c = 0.11\ln(\sigma_s/F_y) + 0.22$	$w_c = 0.005\ln(\sigma_s/F_y) + 0.044$
	$R^2 = 0.97$	$R^2 = 0.96$	$R^2 = 0.128$
M9-FA-12-(B)	$w_c = 0.30\ln(\sigma_s/F_y) + 0.49$	$w_c = 0.12\ln(\sigma_s/F_y) + 0.23$	$w_c = 0.0004\ln(\sigma_s/F_y) + 0.0392$
	$R^2 = 0.94$	$R^2 = 0.98$	$R^2 = 0.0009$
M9-FA-16-(A)	$w_c = 0.18\ln(\sigma_s/F_y) + 0.48$	$w_c = 0.08\ln(\sigma_s/F_y) + 0.22$	$w_c = 0.005\ln(\sigma_s/F_y) + 0.044$
	$R^2 = 0.99$	$R^2 = 0.96$	$R^2 = 0.219$
M9-FA-16-(B)	$w_c = 0.17\ln(\sigma_s/F_y) + 0.32$	$w_c = 0.08\ln(\sigma_s/F_y) + 0.18$	$w_c = -0.001\ln(\sigma_s/F_y) + 0.040$
	$R^2 = 0.95$	$R^2 = 0.98$	$R^2 = 0.011$
M9-FA-16-(C)	$w_c = 0.21\ln(\sigma_s/F_y) + 0.41$	$w_c = 0.08\ln(\sigma_s/F_y) + 0.19$	$w_c = 0.002\ln(\sigma_s/F_y) + 0.039$
	$R^2 = 0.94$	$R^2 = 0.98$	$R^2 = 0.051$
M9-FA-16-(D)	$w_c = 0.21\ln(\sigma_s/F_y) + 0.41$	$w_c = 0.10\ln(\sigma_s/F_y) + 0.21$	$w_c = 0.008\ln(\sigma_s/F_y) + 0.043$
	$R^2 = 0.97$	$R^2 = 0.99$	$R^2 = 0.120$
M1-OPC-12-(A)	$w_c = 0.39\ln(\sigma_s/F_y) + 0.71$	$w_c = 0.15\ln(\sigma_s/F_y) + 0.33$	$w_c = -0.01\ln(\sigma_s/F_y) + 0.04$
	$R^2 = 0.78$	$R^2 = 0.88$	$R^2 = 0.13$
M1-OPC-12-(B)	$w_c = 0.21\ln(\sigma_s/F_y) + 0.53$	$w_c = 0.14\ln(\sigma_s/F_y) + 0.33$	$w_c = 0.06\ln(\sigma_s/F_y) + 0.12$
	$R^2 = 0.87$	$R^2 = 0.87$	$R^2 = 0.20$
M2-OPC-16-(A)	$w_c = 0.27\ln(\sigma_s/F_y) + 0.63$	$w_c = 0.09\ln(\sigma_s/F_y) + 0.23$	$w_c = 0.01\ln(\sigma_s/F_y) + 0.06$
	$R^2 = 0.93$	$R^2 = 0.97$	$R^2 = 0.45$
M2-OPC-16-(B)	$w_c = 0.34\ln(\sigma_s/F_y) + 0.74$	$w_c = 0.08\ln(\sigma_s/F_y) + 0.22$	$w_c = 0.01\ln(\sigma_s/F_y) + 0.05$
	$R^2 = 0.94$	$R^2 = 0.97$	$R^2 = 0.13$
